# The Effects of Charged Amino Acid Side-Chain Length on Diagonal Cross-Strand Interactions between Carboxylate- and Ammonium-Containing Residues in a β-Hairpin

**DOI:** 10.3390/molecules27134172

**Published:** 2022-06-29

**Authors:** Jing-Yuan Chang, Yen-Jin Pan, Pei-Yu Huang, Yi-Ting Sun, Chen-Hsu Yu, Zhi-Jun Ning, Shou-Ling Huang, Shing-Jong Huang, Richard P. Cheng

**Affiliations:** 1 Department of Chemistry, National Taiwan University, Taipei 10617, Taiwan; r08223149@ntu.edu.tw (J.-Y.C.); r03223211@ntu.edu.tw (Y.-J.P.); r03223210@ntu.edu.tw (P.-Y.H.); b99203034@ntu.edu.tw (Y.-T.S.); r05223205@ntu.edu.tw (C.-H.Y.); r09223205@ntu.edu.tw (Z.-J.N.); 2 Instrumentation Center, National Taiwan University, Taipei 10617, Taiwan; shouling@g.ntu.edu.tw (S.-L.H.); shingjonghuang@ntu.edu.tw (S.-J.H.)

**Keywords:** peptide, β-hairpin, diagonal interaction, ion-pairing interaction, charged amino acid, side-chain length

## Abstract

The β-sheet is one of the common protein secondary structures, and the aberrant aggregation of β-sheets is implicated in various neurodegenerative diseases. Cross-strand interactions are an important determinant of β-sheet stability. Accordingly, both diagonal and lateral cross-strand interactions have been studied. Surprisingly, diagonal cross-strand ion-pairing interactions have yet to be investigated. Herein, we present a systematic study on the effects of charged amino acid side-chain length on a diagonal ion-pairing interaction between carboxylate- and ammonium-containing residues in a β-hairpin. To this end, 2D-NMR was used to investigate the conformation of the peptides. The fraction folded population and the folding free energy were derived from the chemical shift data. The fraction folded population for these peptides with potential diagonal ion pairs was mostly lower compared to the corresponding peptide with a potential lateral ion pair. The diagonal ion-pairing interaction energy was derived using double mutant cycle analysis. The Asp2-Dab9 (Asp: one methylene; Dab: two methylenes) interaction was the most stabilizing (−0.79 ± 0.14 kcal/mol), most likely representing an optimal balance between the entropic penalty to enable the ion-pairing interaction and the number of side-chain conformations that can accommodate the interaction. These results should be useful for designing β-sheet containing molecular entities for various applications.

## 1. Introduction

The β-sheet is one of the common protein secondary structures, with 23% of the protein residues adopting a β-sheet conformation [1,2,3]. Aberrant aggregation of β-sheets form amyloid fibrils involved in a number of neurodegenerative diseases including Alzheimer’s disease [4,5], Huntington’s disease [6,7], and Parkinson’s disease [8]. As such, studies on β-sheet stability and formation may provide insight into the emergence and treatment of these neurodegenerative diseases.

β-Sheet stability is determined by both the thermodynamic sheet propensity of the constituting residues [9,10] and cross-strand interactions [11,12,13]. A survey focusing on antiparallel β-sheets highlighted the importance of lateral cross-strand interactions between oppositely charged residues [11]. Accordingly, lateral cross-strand interactions have been studied in various sheet-containing host systems, including the protein G B1 domain [14,15], a zinc finger domain [16], and β-hairpins [17,18,19,20,21,22,23,24,25,26,27]. In the protein G B1 domain system, both lateral Glu-Lys and the Glu-Arg interactions provided approximately 1.0 kcal/mol stabilization based on thermal denaturation experiments [14]. In the zinc-finger system, the lateral cross-strand interaction between negatively charged residues (Asp and Glu) and positively charged residues (Arg and Lys) were 0.02–0.48 kcal/mol based on competitive metal ion binding studies [16]. In β-hairpins, lateral cross-strand Glu–Lys ion-pairing interactions provided 0.1–0.3 kcal/mol stabilization based on NMR studies [20,22,23]. Furthermore, various types of lateral cross-strand interactions have been investigated in β-hairpins, including hydrophobics [17,24], electrostatics [17,20,22,25,26,27], and aromatic π–π interactions [21,22]. Nonetheless, statistical studies showed that diagonal cross-strand interactions are much more significant, compared to lateral cross-strand interactions across antiparallel β-strands [12].

Diagonal cross-strand interactions are enabled by the inherent right-handed twist of β-sheet structures [19,27,28]. Accordingly, a number of diagonal cross-strand interactions have been investigated in β-hairpins, including hydrophobics [24], cation–π [29,30,31,32], carbohydrate–π [33,34], and amide–π interactions [35]. These diagonal cross-strand interactions stabilized the β-hairpin structure by 0.1–1.0 kcal/mol [24,29,30,31,32,33,34,35]. Surprisingly, the diagonal cross-strand interactions between oppositely charged residues (i.e., ion-pairing interactions) have yet to be investigated. Herein, we present a systematic study on the effects of charged amino acid side-chain length on diagonal cross-strand interactions between carboxylate- and ammonium-containing residues in a β-hairpin.

## 2. Results

### 2.1. Peptide Design and Synthesis

**Figure 1:**
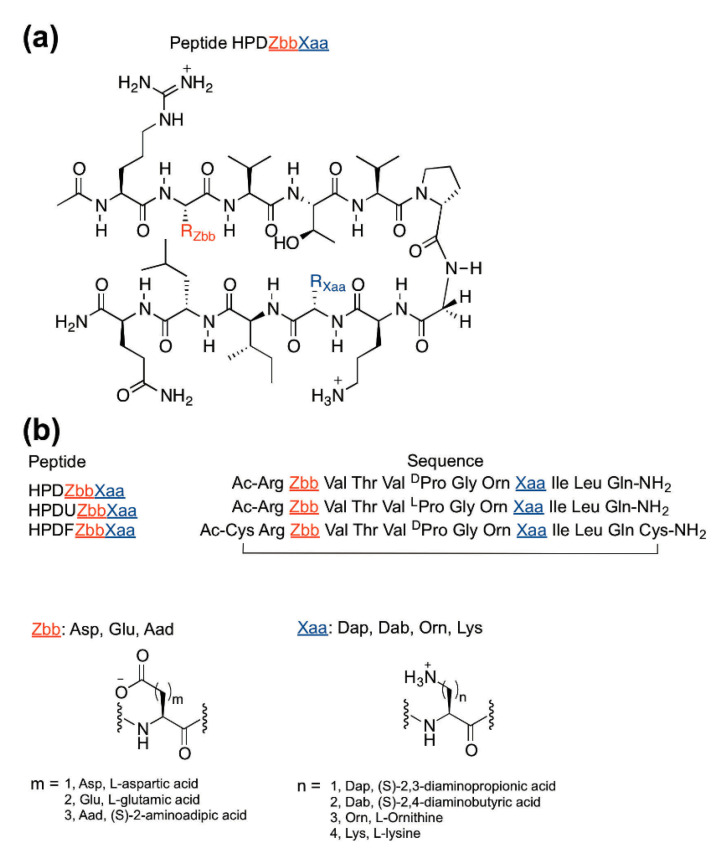
Design of peptides to study the effects of charged amino acid side-chain length on diagonal cross-strand ion-pairing interactions: (**a**) the chemical structure of the experimental HPDZbbXaa peptides; (**b**) the sequences of the experimental HPDZbbXaa peptides, the fully unfolded reference HPDUZbbXaa peptides, and the fully folded reference HPDFZbbXaa peptides.

The experimental HPDZbbXaa peptides were designed based on Gellman’s YKL peptide [19] and the HPTZbbXaa hairpin peptides described in our previous studies (Figure 1) [3,25,26]. A diagonal cross-strand cation–π interaction was observed in the parent YKL β-hairpin peptide [19]. Furthermore, diagonal cross-strand NOEs were observed between positions 2 and 9 in the HPTZbbXaa peptides [3,25,26,27]. Therefore, the Tyr2 (in peptide YKL) was replaced with negatively charged carboxylate-containing residues to interact with the positively charged ammonium-containing residues at position 9. The Glu4 (in peptide YKL) was replaced with Thr to maintain the β-structure based on the high thermodynamic sheet propensity of Thr [9,10] and to remove the potential lateral ion-pairing interaction with position 9. An acetyl group and a carboxamide group were incorporated at the N- and C-termini, respectively, to minimize unexpected interactions involving the charged termini [17]. To investigate the effects of charged amino acid side-chain length on diagonal cross-strand ion-pairing interactions, the negatively charged carboxylate-containing residues (Zbb = Asp, Glu, Aad) and positively charged ammonium-containing residues (Xaa = Dap, Dab, Orn, Lys) were incorporated at positions 2 and 9, respectively, to give the experimental HPDZbbXaa peptides (Figure 1). The experimental peptides were named HPDZbbXaa, representing a hairpin peptide to study the diagonal interactions, followed by the negatively charged Zbb residue at position 2 and the positively charged Xaa residue at position 9.

The fully folded reference peptides HPDFZbbXaa and the fully unfolded reference peptides HPDUZbbXaa were required to determine the fraction folded population of the experimental HPDZbbXaa peptides (Figure 1b) [3,19,25,26,27,36,37,38]. The fully folded reference HPDFZbbXaa peptides were designed by introducing cysteines at both the N- and C-termini of experimental HPDZbbXaa peptides to form intramolecular disulfide bonds [3,19,25,26,27,36,37,38]. The fully unfolded reference HPDUZbbXaa peptides were designed by replacing DPro with LPro at position 6 of the experimental HPDZbbXaa peptides, to disrupt the β-hairpin conformation [3,19,25,26,27,36,37,38].

The peptides were synthesized by solid-phase peptide synthesis using Fmoc-based chemistry [39,40]. The intramolecular disulfide bond in the fully folded reference HPDFZbbXaa peptides was formed via charcoal-mediated air oxidation [41]. All peptides were purified by reverse-phase high-performance liquid chromatography (RP–HPLC) to higher than 95% purity (except for HPDAspDap, HPDAspDab, HPDGluDap, HPDGluDab, and HPDAadDab, which were purified to higher than 90% purity), and confirmed by matrix-assisted laser desorption ionization time-of-flight (MALDI–TOF) mass spectrometry.

### 2.2. Peptide Structure Characterization by NMR Spectroscopy

The peptides were analyzed by two-dimensional NMR spectroscopy including total correlation spectroscopy (TOCSY), double-quantum filtered-correlated spectroscopy (DQF-COSY), and rotating-frame nuclear Overhauser spectroscopy (ROESY) at 298 K. Sequence-specific assignments of the chemical shifts were performed based on the TOCSY and ROESY spectra (Tables S1–S36) [42]. The number of major spin systems was consistent with the number of residues in each peptide. Weak cross-peaks corresponding to minor conformations were observed in the TOCSY spectra for many of the HPDZbbXaa and HPDUZbbXaa peptides. Since the NMR spectra (chemical shift and peak width) of analogous hairpin peptides did not change with concentration (20 μM to 10 mM) [3,19,43,44], the peptides in this study (0.9–10.1 mM) should not aggregate in solution. Accordingly, the experimental data should reflect the intramolecular interactions with minimal interference from intermolecular interactions.

The chemical shift dispersion of the peptides was evaluated using the chemical shift ranges of the HN and Hα protons, and the standard deviations of the average chemical shifts of the HN and Hα protons (Table 1). The broader the chemical shift range, the higher the chemical shift dispersion. Additionally, the higher the standard deviation of the average chemical shift, the higher the chemical shift dispersion. For a given peptide, the chemical shift range was broader for the Hα signals than for the HN signals (Table 1). Similarly, the standard deviation of the average chemical shift was higher for the Hα signals than for the HN signals. The HN and Hα chemical shift ranges followed the trend HPDFZbbXaa > HPDZbbXaa > HPDUZbbXaa, except for the HN chemical shift range for HPDAspDap versus HPDUAspDap (Table 1). Nonetheless, the Hα chemical shift range for this exception followed the aforementioned general trend. The standard deviation of the average chemical shifts of the HN and Hα protons followed the trend HPDFZbbXaa > HPDZbbXaa > HPDUZbbXaa, except for the standard deviation of the average HN chemical shifts for HPDAspDap versus HPDUAspDap (Table 1). Nevertheless, the standard deviation of the average Hα chemical shift for this exception followed the aforementioned general trend. The exception in the HN chemical shift dispersion for HPDAspDap versus HPDUAspDap was most likely because the HN chemical shift is more sensitive to the neighboring residues in the primary sequence, whereas the Hα chemical shift is more sensitive to the structural environment [45]. Accordingly, the HN chemical shift dispersion could be either higher than or similar to the Hα chemical shift dispersion in the unfolded form, whereas the HN chemical shift dispersion could be lower than the Hα chemical shift dispersion in the folded form [45], thereby resulting in the exception in the HN chemical shift dispersion for HPDAspDap versus HPDUAspDap. Overall, the chemical shift dispersion of peptides with a given Zbb2-Xaa9 pair followed the trend HPDFZbbXaa > HPDZbbXaa > HPDUZbbXaa (Table 1 and Tables S1–S36). Since the higher the chemical shift dispersion, the higher the folded population [45], these results were consistent with the intended designs.

**Figure 2:**
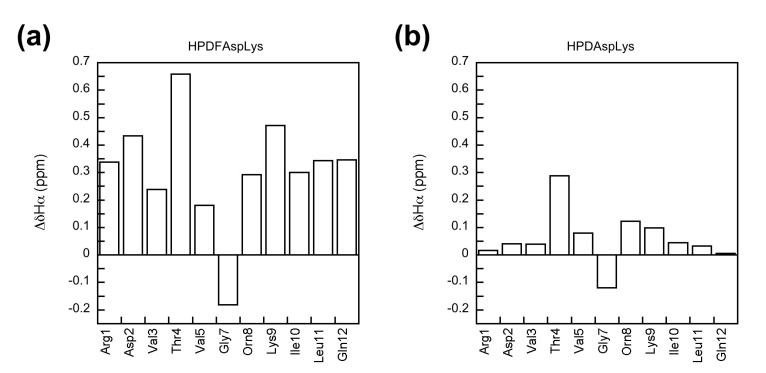
The chemical shift deviation (ΔδHα) for the residues in peptides HPDFAspLys (**a**) and HPDAspLys (**b**).

The β-hairpin structure of the peptides was confirmed by the chemical shift deviation of the Hα signals, *^3^J_NHα_* spin–spin coupling constants, and cross-strand NOE signals. The Hα chemical shift deviation (ΔδHα) is the difference between the Hα signal of the residue of interest and the corresponding Hα signal of the residue in the random coil conformation. The fully unfolded reference peptides were assumed to be random coil in this study [3,19,25,26,27,37]. A positive ΔδHα value represents an extended β-sheet conformation [46,47]. Residues Zbb2 through Val5 and Orn8 through Leu11 showed positive ΔδHα values for all of the experimental HPDZbbXaa peptides and all the fully folded reference HPDFZbbXaa peptides (Figure 2, Figures S1 and S2), consistent with an extended β-strand conformation for these strand residues. The ΔδHα values of terminal residues Arg1 and Gln12 for the experimental peptides were close to zero (Figure 2b and Figure S1), most likely resulting from the end fraying effect [3,23,25,26,27]. The ΔδHα values of Gly7 were negative or close to zero for all peptides, consistent with a turn conformation for Gly7 [43]. In general, residues in the strand regions (residues 2–5 and 8–11) showed more positive ΔδHα values for the fully folded reference peptides, compared to those in the corresponding experimental peptides, suggesting that the fully folded reference peptides exhibited a higher β-hairpin population than the corresponding experimental peptides. This was consistent with the intended designs.

The *^3^J_NHα_* spin–spin coupling constants for each residue in the peptides were determined using the DQF-COSY spectra (Tables S37–S45) [48]. The *^3^J_NHα_* values for the strand residues in the experimental HPDZbbXaa peptides were higher than 7 Hz (Tables S37–S39), suggesting a β-conformation [42,49]. The *^3^J_NHα_* values for the strand residues in the fully folded reference HPDFZbbXaa peptides were also higher than 7 Hz, and most of the values were higher than those for the corresponding residues in the experimental HPDZbbXaa peptides (Table 2 and Tables S37–S42). This suggested that the fully folded reference HPDFZbbXaa peptides were more folded than the corresponding experimental HPDZbbXaa peptides. If one disregards the standard deviations, most of the average *^3^J_NHα_* values followed the trend HPDFZbbXaa > HPDZbbXaa > HPDUZbbXaa (Table 2). Overall, these trends were consistent with the intended designs.

All cross-peaks in the ROESY spectra were assigned. Intra-residue, sequential, medium-range, and long-range NOEs (Figures S3–S50) with a number of cross-strand Hα-to-Hα, Hα-to-NH, NH-to-NH correlations were observed in the ROESY spectra (Figures S39–S50). Strong sequential Hα*_i_*-NH*_i_*_+1_ correlations were observed for every strand on all peptides, suggesting an extended β-strand conformation (Figures S39–S50). The cross-strand NOE connectivities obtained from the ROESY spectra further supported the intended β-hairpin conformation for the fully folded reference HPDFZbbXaa peptides and the experimental HPDZbbXaa peptides (Figures S3–S38). For a given Zbb2-Xaa9 combination, the number of cross-strand NOE connectivities (including both side-chain and backbone) followed the trend HPDFZbbXaa > HPDZbbXaa > HPDUZbbXaa (Figures S3–S50). Since more cross-strand NOE connectivities should correspond to a higher fraction β-hairpin structure, these trends are consistent with the intended designs.

### 2.3. Fraction Folded Population and Folding Free Energy

The fraction folded population and folding free energy (ΔG_fold_) for each residue on the experimental HPDZbbXaa peptides were derived from the Hα chemical shift data (Figures S51 and S52). The ratio of the chemical shift deviation (ΔδHα) for a given residue on the experimental HPDZbbXaa peptide to the corresponding value on the fully folded reference HPDFZbbXaa peptide gave the fraction folded population for the residue of interest in the experimental peptide (Figure S51) [3,19,25,26,27,37]. The average of the fraction folded population for residues at positions 2, 3, 9, and 10 was used to represent the fraction folded population of the corresponding peptide (Table 3) [3,25,26,27]. These residues were not at the termini of the peptide, at which residues suffered from end fraying effects, nor were these residues adjacent to the turn, at which residues exhibited intrinsically higher fraction folded population [3,19,23,25,26,27]. Furthermore, both strands, and both hydrogen and non-hydrogen bonded positions, were equally represented in the chosen residues [3,25,26,27]. Similarly, the average of the ΔG_fold_ for residues at positions 2, 3, 9, and 10 was used to represent the ΔG_fold_ of the corresponding peptide (Table 4) [3,25,26,27]. Since the ΔG_fold_ exhibited the same trends as the fraction folded population (i.e., the higher the fraction folded population, the less positive the ΔG_fold_), only the fraction folded population data are discussed in detail.

The fraction folded population for HPDZbbXaa peptides was between 15% and 47% with standard deviations within 5% (Table 3). HPDAadDap exhibited the highest fraction folded population among all HPDZbbXaa peptides. HPDAspLys exhibited the least fraction folded population among the peptides. If one disregards HPDAspDap, the fraction folded population of HPDZbbXaa peptides with a given negatively charged residue Zbb2 generally decreased as the side-chain length of the positively charged residue Xaa9 increased. Furthermore, the fraction folded population of HPDZbbXaa peptides with a given positively charged residue Xaa9 generally increased as the side-chain length of the negatively charged residues Zbb2 increased from Asp to Glu but mostly remained similar upon increasing Glu to Aad.

### 2.4. Diagonal Cross-Strand Zbb-Xaa Interactions

The interaction free energy (ΔG_int_) for each potential diagonal Zbb2-Xaa9 interaction was derived by double mutant cycle analysis (Table 5) [50]. The double mutant cycle was used to remove the inherent effect of individually incorporating the charged residues Zbb2 and Xaa9 from the effect of simultaneously incorporating both Zbb2 and Xaa9 [25,26,27]. The energy difference between the ΔG_fold_ of HPDZbbXaa and HPDAlaAla represented the effect of simultaneously incorporating both Zbb2 and Xaa9. The energy difference between the ΔG_fold_ of HPDZbbAla (and HPDAlaXaa) and HPDAlaAla represented the effect of individually incorporating Zbb2 (and Xaa9) on β-hairpin stability. The Zbb2-Xaa9 interaction energy (ΔG_int_) was derived from the folding free energy for the peptides HPDZbbXaa, HPDZbbAla [38], HPDAlaXaa [38], and HPDAlaAla (Table 5).

Most of the cross-strand diagonal Zbb2-Xaa9 interactions were apparently stabilizing (Table 5). However, there was no apparent Aad2-Orn9 or Aad2-Lys9 interaction. Nonetheless, the Asp2-Dab9 interaction was the most stabilizing, providing up to −0.794 kcal/mol stabilization. The Zbb2-Dap9 interactions were similar in energy regardless of the negatively charged Zbb2 residue. For the HPDZbbDab and HPDZbbOrn peptides, the ΔG_int_ for the Zbb2-Dab9 and Zbb2-Orn9 interactions followed the trend Asp < Glu < Aad. For the HPDZbbLys peptides, the ΔG_int_ for the Zbb2-Lys9 interaction followed the trend Asp > Glu < Aad. If one disregards the Asp2-Dap9 and Glu2-Dap9 interactions, the ΔG_int_ for the Zbb2-Xaa9 interaction was less stabilizing upon increasing the Xaa9 side-chain length for a given negatively charged Zbb2 residue.

## 3. Discussion

The effects of side-chain length on diagonal ion-pairing interactions between carboxylate- and ammonium-containing residues in a β-hairpin was investigated. The fraction folded population for the experimental HPDZbbXaa peptides was 15–47% (Table 3). The variation in the fraction folded population of HPDZbbXaa peptides can be rationalized by the intrinsic sheet propensity of the carboxylate- and ammonium-containing amino acids with different side-chain lengths at positions 2 and 9, respectively, and the potential cross-strand diagonal Zbb2-Xaa9 ion-pairing interaction. In general, the fraction folded population for the HPDZbbXaa peptides with a given positively charged residue Xaa9 increased as the side-chain length of the negatively charged residues Zbb2 increased from Asp to Glu but mostly remained similar upon increasing Glu to Aad (Table 3). This trend is similar to the fraction folded population trend for the the HPDZbbAla peptides (with the negatively charged residue Zbb at position 2 and Ala at position 9) [38], suggesting that there may be small variations in the diagonal Zbb2-Xaa9 interactions in the HPDZbbXaa peptides. However, the fraction folded population for the HPDZbbXaa peptides with a given negatively charged residue Zbb2 generally decreased as the side-chain length of the positively charged residue Xaa9 increased (Table 3). This trend is opposite of the fraction folded population trend for the HPDAlaXaa peptides (with Ala at position 2 and the positively charged ammonium-containing residue Xaa at position 9) [38], suggesting variation in the diagonal Zbb2-Xaa9 interaction in the HPDZbbXaa peptides. Indeed, the diagonal Zbb2-Xaa9 interaction varied with side-chain length combination (Table 5).

How do the HP**D**ZbbXaa peptides with a diagonal Zbb2-Xaa9 interaction measure up to the HP**T**ZbbXaa peptides with a lateral Zbb4-Xaa9 interaction [25]? The constituting amino acids for the two sets of peptides were the same, but Thr and Zbb were incorporated at positions 2 and 4, respectively, in the HPTZbbXaa peptides [25]. The fraction folded population of the HPDZbbXaa peptides was mostly lower than that of the corresponding HPTZbbXaa peptides [25], except for HPDGluDab, HPDAadDap, and HPDAadDab. Furthermore, the range for the fraction folded population of the HPDZbbXaa peptides was narrower than that of the HPTZbbXaa peptides (24–63%) [25]. The HPDAadDap (47 ± 4%) and HPDGluDab (42 ± 3%) peptides exhibited the highest fraction folded populations for the HPDZbbXaa peptides in this study. Surprisingly, HPTAadDap (25 ± 1%) and HPTGluDab (24 ± 2%) exhibited the lowest fraction folded populations for the HPTZbbXaa peptides [25]. These differences may be due to the effect of incorporating the negatively charged residue at position 2 versus 4, the effect of incorporating Thr at position 4 versus 2, and the difference in the diagonal Zbb2-Xaa9 interaction and the lateral Zbb4-Xaa9 interaction. The fraction folded populations of HPDAspAla (8 ± 5%) and HPDGluAla (19 ± 5%) were lower than those of HPTAspAla (37 ± 1%)) and HPTGluAla (29 ± 2%) [3,38], respectively, whereas the fraction folded populations of HPDAadAla (26 ± 4%) and HPTAadAla (25 ± 2%) were essentially the same [3,38]. Furthermore, the fraction folded populations for HPDAlaDab (21 ± 3%) and HPDAlaLys (27 ± 3%) were higher than those for HPTAlaDab (14 ± 3%) and HPTAlaLys (22 ± 3%) [3,38], respectively, whereas the fraction folded populations of HPDAlaDap (19 ± 6%) and HPDAlaOrn (24 ± 3%) were similar to those of HPTAlaDap (16 ± 1%) and HPTAlaOrn (21 ± 3%) [3,38], respectively. Both HPDAadDap and HPDGluDab exhibited relatively high fraction folded populations, most likely because the relatively longer Aad and Glu side chains favor strand formation [38], coupled with reasonably stabilizing diagonal Aad2-Dap9 and Glu2-Dab9 ion-pairing interaction (Table 5), respectively.

**Figure 3:**
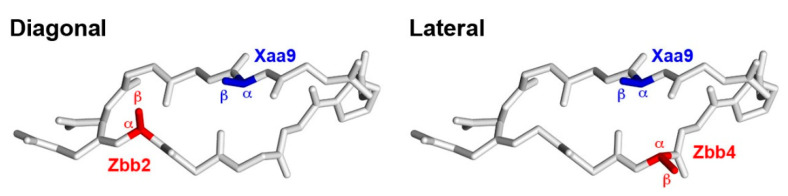
The backbone of the solution structure of an analogue of the parent YKL peptide (pdb code 1JY9 [51]) along with the intended diagonal (**left**) and lateral (**right**) interacting residues. The backbone and DPro side chains are shown in white. The Cα and Cβ of the negatively charged residues are shown in red, and Cα and Cβ of the positively charged residues are shown in blue.

The diagonal ion-pairing interaction between carboxylate- and ammonium-containing residues with varying side-chain lengths provided up to 0.79 kcal/mol stabilization in β-hairpins (Table 5). This is on par with the diagonal hydrophobics/π-related interactions, which provided up to 1.0 kcal/mol stabilization in β-hairpins [24,29,30,31,32,33,34,35]. Furthermore, the diagonal ion-pairing interaction in this study also matched the analogous lateral ion-pairing interaction, which provided up to 0.83 kcal/mol stabilization in β-hairpins [25]. Focusing on the encoded amino acids, the diagonal Asp2-Lys9 (−0.21 kcal/mol) and Glu2-Lys9 (−0.26 kcal/mol) interactions were more stabilizing than the lateral Asp4-Lys9 (−0.11 kcal/mol), Glu4-Lys9 (−0.02 kcal/mol), Lys4-Asp9 (destabilizing), and Lys4-Glu9 (destabilizing) interactions [3,27]. The diagonal Asp2-Dab9 and Glu2-Dab9 interactions were the most stabilizing among the diagonal Zbb2-Xaa9 pairs. However, the lateral Asp4-Dab9 and Glu4-Dab9 pairs showed no apparent interaction in our previous study [25]. This may be due to the difference in the relative positioning of the potentially interacting residues for the diagonal and lateral interactions. Based on the solution structure of an analogue of the parent YKL peptide [51], the Cα-Cα distance for the diagonal Zbb2-Xaa9 pair is 5.91 Å (591 pm), whereas the Cα-Cα distance for the lateral Zbb4-Xaa9 pair is 3.96 Å (396 pm). Furthermore, the Cβ-Cβ distance for the diagonal Zbb2-Xaa9 pair is 4.85 Å (485 pm), whereas the Cβ-Cβ distance for the lateral Zbb4-Xaa9 pair is 5.41 Å (541 pm). According to this solution structure [51], diagonal pairs point toward each other, whereas lateral pairs point away from each other (Figure 3).

The ΔG_int_ for the diagonal Zbb2-Dap9 interactions were similar regardless of the negatively charged Zbb2 side-chain length. The Asp2-Dap9 ion pair should suffer the least side-chain conformational entropic penalty for ion pair formation, compared to the other Zbb2-Dap9 ion-pairs, because the Asp side-chain length is the shortest among the carboxylate-containing residues. Increasing the carboxylate-containing Zbb2 side-chain length would increase the side-chain conformational entropic penalty for the Zbb2-Dap9 ion-pair formation but would also increase the number of side-chain conformations that could accommodate a Zbb2-Dap9 ion-pair to compensate for the increased entropic penalty for ion-pair formation, resulting in similar ΔG_int_ values. Increasing the Dap side-chain length in the Asp2-Dap9 ion-pair to the Asp2-Dab9 ion pair resulted in the most stabilizing diagonal ion-pairing interaction, suggesting that the increase in the number of side-chain conformations that could accommodate an Asp2-Dab9 ion-pair outweighed the increase in the side-chain conformational entropic penalty for ion-pair formation upon lengthening Dap9 to Dab9. Further side-chain lengthening of either Asp2 or Dab9 (or both) resulted in a drop in stabilization for the Zbb2-Xaa9 interaction, suggesting that the increase in the number of conformations that could accommodate a Zbb2-Xaa9 ion-pair could no longer compensate for the increase in the entropic penalty for ion-pair formation. As such, the diagonal Asp2-Dab9 ion-pairing interaction most likely represents an optimal balance between side-chain conformational entropic penalty and the number of side-chain conformations to support the Asp2-Dab9 interaction. This is in sharp contrast to the length matching requirements for the lateral Zbb4-Xaa9 interaction [25], in which the longer side chains most likely interact through hydrophobics and the shorter side chains most likely interact through electrostatics.

## 4. Materials and Methods

### 4.1. Peptide Synthesis and Purification

Peptides were synthesized by solid-phase peptide synthesis using Fmoc-based chemistry [39,40]. The disulfide bond in the HPDFZbbXaa peptides was formed via charcoal-mediated air oxidation [41]. All peptides were purified by reverse-phase high-performance liquid chromatography (RP–HPLC) to higher than 95% purity (except for HPDAspDap, HPDAspDab, HPDGluDap, HPDGluDab, and HPDAadDab, which were purified to higher than 90% purity). The identity of the peptides was confirmed by matrix-assisted laser desorption ionization time-of-flight mass spectroscopy (MALDI–TOF). More detailed procedures and peptide characterization data are provided in the Supplementary Materials.

### 4.2. Chemical Shift Deviation

Purified peptides were dissolved in a H_2_O/D_2_O (9:1 ratio by volume) in the presence of 50 mM sodium deuterioacetate buffer (pH 5.5, uncorrected) to give peptide concentrations of 0.9–10.1 mM. 2-Dimethyl-2-silapentane-5-sulfonate was added to the sample as an internal reference. All NMR experiments were performed on a Brüker AV III 800 MHz spectrometer. Phase-sensitive total correlation spectroscopy (TOCSY) [52], rotating-frame nuclear Overhauser effect spectroscopy (ROESY) [53], and double-quantum filtered-correlated spectroscopy (DQF-COSY) [54] experiments were performed by collecting 2048 points in f2 with 4–8 scans and 256–512 points in f1 at 298 K. Solvent suppression was achieved by the WATERGATE solvent suppression sequence [55]. TOCSY and ROESY experiments employed a spin locking field of 10 kHz. Mixing times of 60 and 200 ms were used for the TOCSY and ROESY experiments, respectively.

### 4.3. Nuclear Magnetic Resonance Spectroscopy

Sequence-specific assignments for all peptides were completed by using the 2D-NMR spectra (TOCSY and ROESY). The chemical shift deviation (ΔδHα) for each residue of the experimental peptide (ΔδHα(exp)) and the folded reference peptide (ΔδHα(F)) was derived using Equations (1) and (2), respectively [46]. δHα(exp) is the chemical shift for the residue of interest on the experimental peptide. δHα(U) is the chemical shift for the corresponding residue of interest on the fully unfolded reference peptide. δHα(F) is the chemical shift for the corresponding residue of interest on the fully folded reference peptide.

(1) $$\Delta \delta H\alpha \left(\exp\right)=\delta H\alpha \left(\exp\right)-\delta H\alpha \left(U\right)$$

(2) $$\Delta \delta H\alpha \left(F\right)=\delta H\alpha \left(F\right)-\delta H\alpha \left(U\right)$$

### 4.4. ^3^J_NHα_ Spin–Spin Coupling Constant

The peak-to-peak separation in the absorptive (υ_a_) and dispersive (υ_d_) DQF-COSY spectra was measured to derive the *^3^J_NHα_* coupling constant using the values along the f2 axis. The absorptive (υ_a_) and dispersive (υ_d_) values were used to derive the coupling constants from the square root of the single real root using Equation (3) [48].

(3) $$J^{6}-\upsilon_{a}{}^{2}J^{4}+\left(-\frac{9}{4}\upsilon_{a}{}^{4}+\frac{3}{2}\upsilon_{a}{}^{2}\upsilon_{d}{}^{2}+\frac{3}{4}\upsilon_{d}{}^{4}\right)J^{2}+\frac{81}{64}\upsilon_{a}{}^{6}-\frac{9}{16}\upsilon_{a}{}^{4}\upsilon_{d}{}^{2}-\frac{21}{32}\upsilon_{a}{}^{2}\upsilon_{d}{}^{4}-\frac{1}{16}\upsilon_{d}{}^{6}-\frac{\upsilon_{d}{}^{8}}{64\upsilon_{a}{}^{2}}=0$$

### 4.5. Interproton Distance Determination via NOE Integration

The NOE cross-peaks of all peptides were assigned from the corresponding ROESY spectra. Integration was performed based on a Gaussian peak model to obtain the intensity of cross-peaks. The distance between the two β-hydrogen atoms on the proline side chain (1.77 Å) was set as the standard to derive the interproton distance for the cross-peak of interest using Equation (4). The distances were grouped into short (≤2.5 Å), medium (2.5–3.5 Å), and long (>3.5 Å) for the illustrations in the Wüthrich diagrams (Figures S39–S50).

(4) $$R_{residue}=1.77\times 10^{-10}\times {\left(\frac{I_{standard}}{I_{residue}}\right)}^{\frac{1}{6}}$$

### 4.6. Fraction Folded Population and Folding Free Energy (ΔG_fold_)

The equilibrium constant between the unfolded and folded states of an experimental peptide is the ratio of the folded and unfolded populations. The fraction folded population for each residue was derived from the chemical shift data using Equation (5). The folding free energy ΔG_fold_ for each residue was derived using Equation (6). The fraction folded populations and folding free energy (ΔG_fold_) of peptides were obtained by averaging the corresponding values for residues at positions 2, 3, 9, and 10 [3,19,22,25,26,27,38]. These four positions were in the middle of the strands, neither at the termini (which would suffer from the end fraying effect) nor directly attached to the turn (which would promote sheet formation). Furthermore, the average value of these four positions would provide equal representation for both hydrogen-bonded positions (residues 3 and 10) and non-hydrogen-bonded positions (residues 2 and 9), and also equal representation for both strands in the hairpin (i.e., the N-terminal strand: residues 2 and 3; the C-terminal strand: residues 9 and 10).

(5) $$\mathrm{Fraction}\;\mathrm{Folded}\;\mathrm{Population}=\frac{\delta H\alpha \left(\exp\right)-\delta H\alpha \left(U\right)}{\delta H\alpha \left(F\right)-\delta H\alpha \left(U\right)}\times 100\%$$

(6) $$\Delta G_{fold}=-RTln\frac{\delta H\alpha \left(\exp\right)-\delta H\alpha \left(U\right)}{\delta H\alpha \left(F\right)-\delta H\alpha \left(\exp\right)}$$

### 4.7. Double Mutant Cycle Analysis

Double mutant cycle analysis [50] was performed to determine the interaction free energy (Δ*G_int_*) between charged residues Zbb2 and Xaa9 in HPDZbbXaa peptides using Equation (7). This analysis accounted for the effect of each charged residue (individually) on strand stability using data from the corresponding Ala-containing peptides HPDZbbAla and HPDAlaXaa [38] to determine the Zbb2-Xaa9 ion-pairing interaction exclusively. The peptide with Ala incorporated at positions 2 and 9, HPDAlaAla, was used as the reference peptide.

(7) $$\Delta G_{int}=\left(\Delta G_{HPDZbbXaa}-\Delta G_{HPDAlaAla}\right)-\left(\Delta G_{HPDZbbAla}-\Delta G_{HPDAlaAla}\right)-\left(\Delta G_{HPDAlaXaa}-\Delta G_{HPDAlaAla}\right)$$

## 5. Conclusions

The effects of charged amino acid side-chain length on diagonal cross-strand ion-pairing interaction between carboxylate- and ammonium-containing residues was investigated in a β-hairpin by NMR methods. HPDAadDap exhibited the highest fraction folded population because the long Aad side chain inherently favors strand formation coupled with the reasonably stabilizing diagonal Aad2-Dap9 ion-pairing interaction. Furthermore, the diagonal Asp2-Dab9 ion-pairing interaction was the most stabilizing interaction, most likely representing an optimal balance between the side-chain conformational entropic penalty to enable the ion-pairing interaction and the number of side-chain conformations that can accommodate the interaction. These results should be useful for stabilizing β-sheet containing molecular entities for various applications. β-Strand containing motifs have been used for specific recognition of various biomolecules including ATP [56], DNA [57,58], and RNA [59,60,61,62,63,64]. Furthermore, β-sheet containing peptides have been developed for ion channel blocking [65], bacterial endotoxin inhibition [66], antimicrobial activity [67], hydrogel formation [68,69,70,71,72,73,74,75], and intracellular delivery [76,77]. The results in this study may also facilitate the design of β-strand containing molecules for reducing amyloid [78], or for inhibiting protein–protein interactions involving β-structures [79,80].

## Figures and Tables

**Table 1:** The chemical shift range and average chemical shift of the δHN and δHα for the HPDFZbbXaa, HPDZbbXaa, and HPDUZbbXaa peptides.

Peptide	δHN Range ^1^ (ppm)	Average δHN (ppm)	δHα Range ^1^ (ppm)	Average δHα (ppm)
HPDFAspDap	1.145	8.697 ± 0.336	1.428	4.664 ± 0.426
HPDAspDap	0.728	8.406 ± 0.195	1.009	4.374 ± 0.276
HPDUAspDap	0.769	8.366 ± 0.196	0.782	4.351 ± 0.209
HPDFAspDab	1.169	8.662 ± 0.339	1.407	4.649 ± 0.406
HPDAspDab	0.665	8.386 ± 0.171	0.902	4.368 ± 0.265
HPDUAspDab	0.570	8.378 ± 0.153	0.693	4.318 ± 0.177
HPDFAspOrn	1.124	8.637 ± 0.333	1.397	4.622 ± 0.391
HPDAspOrn	0.530	8.346 ± 0.150	0.895	4.338 ± 0.250
HPDUAspOrn	0.429	8.317 ± 0.133	0.723	4.279 ± 0.193
HPDFAspLys	1.108	8.625 ± 0.329	1.373	4.608 ± 0.384
HPDAspLys	0.454	8.326 ± 0.135	0.878	4.328 ± 0.244
HPDUAspLys	0.432	8.302 ± 0.127	0.718	4.276 ± 0.192
HPDFGluDap	1.119	8.740 ± 0.330	1.506	4.673 ± 0.421
HPDGluDap	0.795	8.482 ± 0.206	1.135	4.404 ± 0.294
HPDUGluDap	0.574	8.398 ± 0.172	0.772	4.326 ± 0.187
HPDFGluDab	1.192	8.706 ± 0.337	1.467	4.653 ± 0.404
HPDGluDab	0.626	8.417 ± 0.179	1.008	4.398 ± 0.278
HPDUGluDab	0.373	8.368 ± 0.126	0.514	4.299 ± 0.148
HPDFGluOrn	1.166	8.695 ± 0.343	1.457	4.640 ± 0.394
HPDGluOrn	0.584	8.427 ± 0.176	0.977	4.369 ± 0.258
HPDUGluOrn	0.324	8.348 ± 0.106	0.494	4.289 ± 0.140
HPDFGluLys	1.217	8.686 ± 0.360	1.452	4.630 ± 0.390
HPDGluLys	0.618	8.427 ± 0.179	0.959	4.395 ± 0.237
HPDUGluLys	0.355	8.350 ± 0.102	0.584	4.278 ± 0.165
HPDFAadDap	1.135	8.738 ± 0.335	1.536	4.681 ± 0.424
HPDAadDap	0.793	8.476 ± 0.211	1.140	4.410 ± 0.296
HPDUAadDap	0.587	8.298 ± 0.169	0.782	4.244 ± 0.190
HPDFAadDab	1.183	8.702 ± 0.344	1.458	4.654 ± 0.402
HPDAadDab	0.675	8.445 ± 0.192	0.958	4.390 ± 0.268
HPDUAadDab	0.367	8.355 ± 0.116	0.515	4.300 ± 0.148
HPDFAadOrn	1.182	8.693 ± 0.340	1.442	4.638 ± 0.390
HPDAadOrn	0.535	8.405 ± 0.164	0.906	4.359 ± 0.247
HPDUAadOrn	0.273	8.333 ± 0.092	0.513	4.261 ± 0.163
HPDFAadLys	1.207	8.687 ± 0.357	1.455	4.629 ± 0.387
HPDAadLys	0.599	8.399 ± 0.172	0.927	4.360 ± 0.249
HPDUAadLys	0.276	8.317 ± 0.084	0.530	4.255 ± 0.165

^1^ The chemical shift range was the difference between the chemical shift of the most downfield signal and the chemical shift of the most upfield signal for the proton of interest.

**Table 2:** The average *^3^J_NHα_* coupling constant values for the strand residues in the HPDFZbbXaa, HPDZbbXaa, and HPDUZbbXaa peptides ^1^.

Zbb	Xaa	HPDFZbbXaa	HPDZbbXaa	HPDUZbbXaa
Asp	Dap	11 ± 0.7	9.8 ± 1.0	10 ± 1.2
Asp	Dab	11 ± 1.6	9.4 ± 1.5	8.3 ± 2.2
Asp	Orn	12 ± 0.7	10 ± 1.0	9.7 ± 0.8
Asp	Lys	12 ± 0.6	10 ± 0.8	10 ± 1.0
Glu	Dap	10 ± 0.9	9.9 ± 0.9	9.6 ± 1.3
Glu	Dab	10 ± 1.1	9.5 ± 2.1	9.2 ± 1.6
Glu	Orn	11 ± 0.6	10 ± 0.6	10 ± 0.6
Glu	Lys	11 ± 1.1	9.6 ± 0.9	9.2 ± 1.0
Aad	Dap	11 ± 0.9	9.7 ± 1.4	9.9 ± 1.0
Aad	Dab	10 ± 1.0	9.4 ± 1.7	9.8 ± 1.0
Aad	Orn	12 ± 0.7	10 ± 0.7	9.3 ± 0.5
Aad	Lys	11 ± 0.5	9.9 ± 0.5	9.6 ± 0.7

^1^ The average *^3^J_NHα_* coupling constant values for the strand residues were calculated without including values for Gly, which is in the β-turn.

**Table 3:** The fraction folded population (%) for HPDZbbXaa peptides ^1^.

Xaa9		Zbb2	
	Asp	Glu	Aad
Dap	19 ± 4	38 ± 4	47 ± 4
Dab	25 ± 4	42 ± 3	40 ± 3
Orn	17 ± 4	36 ± 3	33 ± 3
Lys	15 ± 5	36 ± 4	36 ± 3

^1^ Average value for residues 2, 3, 9, and 10.

**Table 4:** The folding free energy (ΔG_fold_, kcal/mol) for HPDZbbXaa peptides ^1^.

Xaa9		Zbb2	
	Asp	Glu	Aad
Dap	0.864 ± 0.177	0.303 ± 0.091	0.062 ± 0.085
Dab	0.665 ± 0.143	0.185 ± 0.065	0.231 ± 0.070
Orn	0.975 ± 0.188	0.347 ± 0.075	0.424 ± 0.077
Lys	1.031 ± 0.231	0.333 ± 0.107	0.345 ± 0.086

^1^ Average value for residues 2, 3, 9, and 10.

**Table 5:** The diagonal Zbb2-Xaa9 ion-pairing interaction energy (ΔG_int_, kcal/mol).

Xaa9		Zbb2	
	Asp	Glu	Aad
Dap	−0.434 ± 0.204	−0.412 ± 0.043	−0.425 ± 0.077
Dab	−0.794 ± 0.139	−0.585 ± 0.058	−0.284 ± 0.030
Orn	−0.375 ± 0.081	−0.291 ± 0.048	−0.023 ± 0.040
Lys	−0.206 ± 0.018	−0.258 ± 0.026	−0.020 ± 0.045

## Data Availability

The data presented in this study are available in the Supplementary Materials. The raw data are available on request from the corresponding author.

## Supplementary Materials

The following are available online at https://www.mdpi.com/article/10.3390/molecules27134172/s1, Tables S1–S36: The ^1^H chemical shift assignments for the peptides, Tables S37–S45: The *^3^J_NHα_* values of the peptides, Figure S1: The Hα chemical shift deviations for the residues in the experimental HPDZbbXaa peptides, Figure S2: The Hα chemical shift deviations for the residues in the fully folded reference HPDFZbbXaa peptides, Figures S3–S38: The NOEs observed involving the side chains of the peptides, Figures S39–S50: Wüthrich diagrams of the backbone NOE connectivities involving the α-protons and amide protons for the peptides, Figure S51: The fraction folded of the residues in the peptides, Figure S52: The ΔG_fold_ of the residues in the peptides.

## Sample Availability

Samples of the compounds are not available from the authors.
