# Supplementary material for: The Effects of Charged Amino Acid Side-Chain Length on Diagonal Cross-Strand Interactions between Carboxylate- and Ammonium-Containing Residues in a β-Hairpin
Source: Molecules. 2022 Jun 29;27(13):4172. doi: 10.3390/molecules27134172 (PMC9268152; doi:10.3390/molecules27134172)
Supplement: Supplementary file 1 [file molecules-27-04172-s001.zip › molecules-1595868-supplementary.pdf]

Supplementary Materials

Effect of Charged Amino Acid Side Chain Length on Diagonal Cross-Strand Interaction between Carboxylate- and Ammonium-Containing Residues in a β-Hairpin

Jing-Yuan Chang, Yen-Jin Pan, Pei-Yu Huang, Yi-Ting Sun, Chen-Hsu Yu, Zhi-Jun Ning, Shou-Ling Huang, Shing-Jong Huang, Richard P. Cheng

Tables

Tables S1~S36. The ^{1}H Chemical Shift Assignments for the Peptides S2~S19

Tables S37~S45. The $^{3}J_{NH\alpha}$ Values of the Peptides S20~S24

Figures

Figures S1. The Hα chemical shift deviations for the residues in the experimental HPDZbbXaa peptides. S25

Figures S2. The Hα chemical shift deviations for the residues in the fully folded reference HPDFZbbXaa peptides. S26

Figures S3~S38. The NOEs observed involving the side chains of the peptides S27~S38

Figures S39~S50. Wüthrich diagrams of the backbone NOE connectivities involving the α-protons and amide protons for the peptides S39~S50

Figure S51. The fraction folded of the residues in HPDZbbXaa peptides S51

Figure S52. The ΔG_{fold} of the residues in HPDZbbXaa peptides S52

Material and Methods S53~S65

References S66

Table S1. The ^{1}H Chemical Shift Assignments for Peptide HPDAspDap

Residue	HN	Hα	Hβ	Others
Ac-		2.034		
Arg1	8.283	4.283	1.727, 1.804	Hγ: 1.622; Hδ: 3.198; NHt:7.207
Asp2[b]	8.456	4.690	2.576, 2.699	
Val3[c]	8.180	4.240	2.132	Hγ: 0.919
Thr4	8.375	4.593	4.058	Hγ: 1.110
Val5[d]	8.351	4.508	2.034	Hγ: 0.939
^{D}Pro6		4.416	1.980, 2.327	Hγ: 2.053; Hδ: 3.794, 3.882
Gly7[e]	8.341	3.848, 3.967		
Orn8[a]	8.200	4.488	1.818, 1.897	Hγ: 1.702, 1.760; Hδ: 3.017
Dap9[a]	8.908	4.857	3.277, 3.461	
Ile10[f]	8.474	4.212	1.858	Hγ: 1.404, 1.154, 0.890 (Me); Hδ: 0.831
Leu11	8.434	4.440	1.604	Hγ: 1.604; Hδ: 0.861, 0.919
Gln12[g]	8.468	4.316	1.976, 2.100	Hγ: 2.352; HNt: 6.869, 7.500
NH_2	7.119, 7.627			

[a]Signal for the terminal HN was not observed. [b]The assignments for the minor Asp2 spin system are 8.590(HN), 4.185(Hα), 2.563, 2.698(Hβ). [c]The assignments for the minor Val3 spin system are 8.084(HN), 4.185(Hα), 2.171(Hβ), 0.924(Hγ). [d]The assignments for the minor Val5 spin system are 8.330(HN), 4.150(Hα), 1.836(Hβ), 0.888(Hγ). [e]The assignments for the minor Gly7 spin system are 8.647(HN), 3.970, 4.030(Hα). [f]The assignments for the minor Ile10 spin system are 8.229 (HN), 4.099 (Hα), 2.047(Hβ), 1.319, 0.890(Hγ), 0.802(Hd). [g]The assignments for the minor Gln12 spin system are 8.394 (HN), 4.298 (Hα), 1.974, 2.098(Hβ), 2.360(Hγ).

Table S2. The ^{1}H Chemical Shift Assignments for Peptide HPDAspDab

Residue	HN	Hα	Hβ	Others
Ac-		2.033		
Arg1	8.287	4.291	1.728, 1.811	Hγ: 1.623; Hδ: 3.195; NHt: 7.204
Asp2[b]	8.447	4.705	2.565, 2.699	
Val3[c]	8.200	4.263	2.115	Hγ: 0.910
Thr4	8.365	4.635	3.998	Hγ: 1.066
Val5[d]	8.473	4.528	2.015	Hγ: 0.939
^{D}Pro6[e]		4.401	1.976, 2.336	Hγ: 2.061; Hδ: 3.804, 3.886
Gly7[f]	8.351	3.803, 3.980		
Orn8	8.058	4.513	1.800, 1.875	Hγ: 1.712; Hδ: 3.018; NHt: 7.615
Dab9[a, g]	8.723	4.660	2.044, 2.147	Hγ: 3.012, 3.056
Ile10	8.469	4.215	1.844	Hγ: 1.158, 1.408, 0.880 (Me); Hδ: 0.822
Leu11	8.401	4.474	1.630	Hγ: 1.575; Hδ: 0.861, 0.919
Gln12[h]	8.476	4.322	1.965, 2.103	Hγ: 2.352; HNt: 6.869, 7.501
NH_2	7.119, 7.648			

[a]Signal for the terminal HN was not observed. [b]The assignments for the minor Asp2 spin system are 8.479(HN), 4.652 (Hα), 2.601, 2.747(Hβ). [c]The assignments for the minor Val3 spin system are 8.281(HN), 4.097(Hα), 2.044(Hβ), 0.802(Hγ). [d]The assignments for the minor Val5 spin system are 8.085(HN), 4.186(Hα), 2.173(Hβ), 0.924(Hγ). [e]The assignments for the minor Pro6 spin system are 4.890(Hα), 1.937, 2.300(Hβ), 2.122(Hγ). [f]The assignments for the minor Gly7 spin system are 8.631(HN), 3.942, 4.018(Hα). [g]The assignments for the minor Dab9 spin system are 8.629(HN), 4.460(Hα), 2.055, 2.143(Hβ), 3.054(Hγ). [h]The assignments

for the minor Gln12 spin system are 8.371(HN), 4.300(Hα) 1.974, 2.096(Hβ), 2.363(Hγ).

Table S3. The [1]H Chemical Shift Assignments for Peptide HPDAspOrn

Residue	HN	Hα	Hβ	Others
Ac-		2.038		
Arg1	8.292	4.279	1.728, 1.816	Hγ: 1.624; Hδ: 3.197; HNt: 7.207
Asp2[a]	8.458	4.689	2.573, 2.699	
Val3[b]	8.149	4.235	2.119	Hγ: 0.919
Thr4[c]	8.290	4.629	3.995	Hγ: 1.075
Val5[d]	8.460	4.522	2.023	Hγ: 0.942
[D]Pro6		4.407	1.965, 2.335	Hγ: 1.986, 2.054; Hδ: 3.796, 3.900
Gly7[e]	8.348	3.794, 3.980		
Orn8	8.038	4.486	1.805, 1.871	Hγ: 1.690, 1.733; Hδ: 3.019; NHt: 7.619
Orn9	8.568	4.460	1.834	Hγ: 1.669, 1.744; Hδ: 2.997; NHt: 7.640
Ile10[f]	8.389	4.186	1.847	Hγ: 1.183, 1.428, 0.883 (Me); Hδ: 0.883
Leu11	8.400	4.420	1.588, 1.636	Hγ: 1.588; Hδ: 0.864, 0.922
Gln12[g]	8.419	4.304	1.971, 2.103	Hγ: 2.356; HNt: 6.875, 7.515
NH_2	7.111, 7.622			

[a]The assignments for the minor Asp2 spin system are 8.476(HN), 4.648(Hα), 2.594, 2.736(Hβ). [b]The assignments for the minor Val3 spin system are 8.083(HN), 4.189(Hα), 2.172(Hβ), 0.923(Hγ). [c]The assignments for the minor Thr4 spin system are 8.273(HN), 4.552(Hα), 3.987(Hβ), 1.074(Hγ). [d]The assignments for the minor Val5 spin system are 8.220(HN), 4.094(Hα), 2.047(Hβ), 0.799, 0.896(Hγ). [e]The assignments for the minor Gly7 spin system are 8.635(HN), 3.940, 4.009(Hα). [f]The assignments for the minor Ile10 spin system are 8.308(HN), 4.133(Hα), 1.825(Hβ), 1.179, 0.887(Hγ). [g]The assignments for the minor Gln12 spin system are 8.360(HN), 4.299(Hα), 1.975, 2.100(Hβ), 2.360(Hγ).

Table S4. The [1]H Chemical Shift Assignments for Peptide HPDAspLys

Residue	HN	Hα	Hβ	Others
Ac-		2.040		
Arg1	8.288	4.283	1.731, 1.818	Hγ: 1.624; Hδ: 3.197; HNt: 7.208
Asp2[a]	8.451	4.681	2.577, 2.688	
Val3[b]	8.164	4.229	2.128	Hγ: 0.924
Thr4	8.274	4.605	4.010	Hγ: 1.084
Val5[c]	8.449	4.509	2.037	Hγ: 0.943
[D]Pro6		4.409	1.973, 2.103	Hγ: 2.051, 2.356; Hδ: 3.793, 3.898
Gly7[d]	8.332	3.803, 3.967		
Orn8	8.024	4.474	1.803, 1.872	Hγ: 1.696, 1.731; Hδ: 3.020; NHt: 7.618
Lys9	8.478	4.402	1.703, 1.762	Hγ: 1.346, 1.437; Hδ: 1.667; Hε: 2.964; NHt: 7.553
Ile10	8.361	4.175	1.845	Hγ: 1.186, 1.439, 0.880 (Me); Hδ: 0.822
Leu11	8.381	4.414	1.583, 1.645	Hγ: 1.583; Hδ: 0.862, 0.924
Gln12[e]	8.387	4.307	1.971, 2.107	Hγ: 2.356; HNt: 6.875, 7.521
NH_2	7.109, 7.622			

[a]The assignments for the minor Asp2 spin system are 8.479(HN), 4.649(Hα), 2.601, 2.743(Hβ). [b]The assignments for the minor Val3 spin system are 8.082(HN), 4.191(Hα), 2.171(Hβ), 0.923(Hγ). [c]The assignments for the minor Val5 spin system are 8.219(HN), 4.096(Hα), 2.047(Hβ), 0.800, 0.897(Hγ). [d]The assignments for the minor Gly7 spin system

are 8.626(HN), 3.936, 4.004(Hα). [e]The assignments for the minor Gln12 spin system are 8.338(HN), 4.300(Hα), 1.975, 2.102(Hβ), 2.361(Hγ).

Table S5. The ^{1}H Chemical Shift Assignments for Peptide HPDFAspDap

Residue	HN	Hα	Hβ	Others
Ac-		2.075		
Cys1	8.441	5.175	2.613, 3.160	
Arg2	8.725	4.588	1.772, 1.815	Hγ: 1.576, 1.644; Hδ: 3.184; NHt: 7.141
Asp3	8.535	4.979	1.623, 1.675	Hγ: 1.437, 1.516; Hδ: 3.178
Val4	8.940	4.412	2.031	Hγ: 0.867
Thr5	8.597	5.062	3.971	Hγ: 1.029
Val6	8.695	4.624	1.941	Hγ: 0.885, 0.919
^{D}Pro7		4.354	1.967, 2.364	Hγ: 2.046, 2.152; Hδ: 3.815, 3.879
Gly8	8.480	3.786, 3.990		
Orn9	8.034	4.696	1.854	Hγ: 1.737, 1.685; Hδ: 3.014; NHt: 7.629
Dap10[a]	8.950	5.214	3.221, 3.484	
Ile11	9.179	4.451	1.862	Hγ: 1.093, 1.346, 0.853 (Me); Hδ: 0.804
Leu12	8.343	4.808	1.537, 1.571	Hγ: 1.475; Hδ: 0.794
Gln13	9.126	4.642	1.913, 2.105	Hγ: 2.241, 2.300; HNt: 6.837, 7.610
Cys14	9.013	5.175	2.613, 3.160	
NH_2	7.243, 7.332			

[a]Signal for the terminal HN was not observed.

Table S6. The ^{1}H Chemical Shift Assignments for Peptide HPDFAspDab

Residue	HN	Hα	Hβ	Others
Ac-		2.072		
Cys1	8.448	5.152	2.586, 3.168	
Arg2	8.731	4.569	1.788	Hγ: 1.621; Hδ: 3.188; NHt: 7.143
Asp3	8.529	4.970	2.427, 2.639	
Val4	8.827	4.507	2.044	Hγ: 0.846, 0.870
Thr5	8.571	4.947	3.930	Hγ: 0.996
Val6	8.832	4.624	1.947	Hγ: 0.918, 0.889
^{D}Pro7		4.348	1.956, 2.366	Hγ: 2.043, 2.150; Hδ: 3.819, 3.870
Gly8	8.457	3.745, 3.994		
Orn9	7.949	4.703	1.808, 1.866	Hγ: 1.689 ; Hδ: 3.012; NHt: 7.610
Dab10[a]	8.758	5.114	1.994, 2.165	Hγ: 2.933, 2.982
Ile11	9.084	4.455	1.806	Hγ: 1.040, 1.319, 0.847 (Me); Hδ: 0.801
Leu12	8.268	4.897	1.547	Hγ: 1.482; Hδ: 0.806
Gln13	9.118	4.644	1.897, 2.106	Hγ: 2.229, 2.280; HNt: 6.833, 7.355
Cys14	9.040	5.068	3.011, 3.138	
NH_2	7.243, 7.616			

[a]Signal for the terminal HN was not observed.

Table S7. The [1]H Chemical Shift Assignments for Peptide HPDFAspOrn

Residue	HN	Hα	Hβ	Others
Ac-		2.069		
Cys1	8.444	5.130	2.661, 3.150	
Arg2	8.720	4.580	1.795	Hγ: 1.581, 1.648; Hδ: 3.185; HNt: 7.140
Asp3	8.500	5.047	2.457, 2.530	
Val4	8.867	4.465	2.031	Hγ: 0.852, 0.872
Thr5	8.468	5.009	3.936	Hγ: 1.003
Val6	8.860	4.620	1.952	Hγ: 0.897, 0.922
^{D}Pro7		4.351	1.962, 2.368	Hγ: 2.045, 2.146; Hδ: 3.834, 3.873
Gly8	8.446	3.733, 4.000		
Orn9	7.970	4.654	1.831	Hγ: 1.682 ; Hδ: 3.007; NHt: 7.617
Orn10[a]	8.577	4.847	1.711, 1.794	Hγ: 1.558, 1.679; Hδ: 2.920
Ile11	9.094	4.447	1.827	Hγ: 1.092, 1.336, 0.841 (Me); Hδ: 0.795
Leu12	8.275	4.783	1.515, 1.624	Hγ: 1.469; Hδ: 0.801, 0.830
Gln13	9.074	4.638	1.903, 2.110	Hγ: 2.235, 2.289; HNt: 6.837, 7.377
Cys14	8.983	5.033	3.004, 3.133	
NH_2	7.240, 7.619			

[a]Signal for the terminal HN was not observed.

Table S8. The [1]H Chemical Shift Assignments for Peptide HPDFAspLys

Residue	HN	Hα	Hβ	Others
Ac-		2.067		
Cys1	8.436	5.113	2.703, 3.139	
Arg2	8.704	4.605	1.803	Hγ: 1.563, 1.656; Hδ: 3.185; HNt: 7.137
Asp3	8.500	5.074	2.468	
Val4	8.896	4.428	2.022	Hγ: 0.873
Thr5	8.473	4.974	3.935	Hγ: 1.007
Val6	8.864	4.610	1.954	Hγ: 0.901, 0.922
^{D}Pro7		4.353	1.963, 2.367	Hγ: 2.045, 2.144; Hδ: 3.831, 3.870
Gly8	8.438	3.740, 3.998		
Orn9	7.954	4.644	1.829	Hγ: 1.680 ; Hδ: 3.007; NHt: 7.614
Lys10[a]	8.509	4.775	1.666	Hγ: 1.258, 1.377; Hδ: 1.589; Hε: 2.891
Ile11	9.062	4.431	1.841	Hγ: 1.116, 1.350, 0.857 (Me); Hδ: 0.795
Leu12	8.288	4.724	1.516, 1.648	Hγ: 1.469; Hδ: 0.805, 0.839
Gln13	9.044	4.648	1.896, 2.111	Hγ: 2.239, 2.292; HNt: 6.842, 7.384
Cys14	8.960	5.008	3.003, 3.128	
NH_2	7.237, 7.623			

[a]Signal for the terminal HN was not observed.

Table S9. The ^{1}H Chemical Shift Assignments for Peptide HPDUAspDap

Residue	HN	Hα	Hβ	Others
Ac-		2.044		
Arg1	8.298	4.264	1.733, 1.812	Hγ: 1.624; Hδ: 3.196; NHt: 7.207
Asp2	8.474	4.646	2.611, 2.728	
Val3[b]	8.044	4.192	2.514	Hγ: 0.923
Thr4[c]	8.299	4.318	4.134	Hγ: 1.180
Val5[d]	8.175	4.428	2.080	Hγ: 0.939, 0.969
^{L}Pro6		4.401	1.933, 2.308	Hγ: 1.991, 2.073; Hδ: 3.706, 3.889
Gly7[e]	8.469	3.970		
Orn8[a, f]	8.306	4.403	1.916	Hγ: 1.701, 1.769; Hδ: 3.008
Dap9[a]	8.813	4.752	3.285, 3.472	
Ile10	8.314	4.156	1.846	Hγ: 1.155, 1.429, 0.890 (Me); Hδ: 0.851
Leu11	8.444	4.385	1.635	Hγ: 1.594; Hδ: 0.873, 0.930
Gln12	8.395	4.298	1.976, 2.103	Hγ: 2.363; HNt: 6.868, 7.520
NH_2	7.109, 7.599			

[a]Signal for the terminal HN was not observed. [b]The assignments for the minor Val3 spin system are 8.102(HN), 4.237(Hα), 2.190(Hβ), 0.930(Hγ). [c]The assignments for the minor Thr4 spin system are 8.237(HN), 4.313(Hα), 4.166(Hβ), 1.200(Hγ). [d]The assignments for the minor Val5 spin system are 7.966(HN), 4.274(Hα), 1.988(Hβ), 0.900(Hγ). [e]The assignments for the minor Gly7 spin system are 8.547(HN), 3.926, 3.988(Hα). [f]The assignments for the minor Orn8 spin system are 8.543(HN), 4.370 (Hα), 1.908(Hβ), 1.765(Hγ), 3.012(Hδ).

Table S10. The ^{1}H Chemical Shift Assignments for Peptide HPDUAspDab

Residue	HN	Hα	Hβ	Others
Ac-		2.039		
Arg1	8.331	4.263	1.733, 1.811	Hγ: 1.625; Hδ: 3.196; NHt: 7.222
Asp2	8.506	4.640	2.597, 2.729	
Val3[b]	8.077	4.190	2.152	Hγ: 0.924
Thr4[c]	8.340	4.314	4.130	Hγ: 1.178
Val5[d]	8.236	4.428	2.078	Hγ: 0.943, 0.973
Pro6		4.400	1.931, 2.317	Hγ: 1.988, 2.063; Hδ: 3.702, 3.892
Gly7[e]	8.493	3.947		
Orn8	8.278	4.356	1.872	Hγ: 1.695, 1.758; Hδ: 3.007; NHt: 7.642
Dab9[a]	8.647	4.464	2.063, 2.151	Hγ: 3.065
Ile10	8.366	4.130	1.837	Hγ: 1.179, 1.462, 0.895 (Me); Hδ: 0.851
Leu11	8.464	4.386	1.645	Hγ: 1.590; Hδ: 0.875, 0.934
Gln12	8.415	4.296	1.978, 2.104	Hγ: 2.367; HNt: 6.899, 7.558
NH_2	7.134, 7.633			

[a]Signal for the terminal HN was not observed. [b]The assignments for the minor Val3 spin system are 8.118(HN), 4.236(Hα), 2.176(Hβ), 0.924(Hγ). [c]The assignments for the minor Thr4 spin system are 8.824(HN), 4.307(Hα), 2.173(Hβ), 1.192(Hγ). [d]The assignments for the minor Val5 spin system are 8.009(HN), 4.269(Hα), 1.984(Hβ), 0.902(Hγ). [e]The assignments for the minor Gly7 spin system are 8.573(HN), 3.897, 3.975(Hα).

Table S11. The ^{1}H Chemical Shift Assignments for Peptide HPDUAspOrn

Residue	HN	Hα	Hβ	Others
Ac-		2.040		
Arg1	8.295	4.265	1.731, 1.823	Hγ: 1.621; Hδ: 3.196; HNt: 7.205
Asp2	8.471	4.644	2.599, 2.725	
Val3[a]	8.043	4.190	2.145	Hγ: 0.921
Thr4[b]	8.301	4.314	4.235	Hγ: 1.196
Val5[c]	8.192	4.427	2.081	Hγ: 0.956
^{L}Pro6		4.399	1.927, 2.308	Hγ: 1.987, 2.062; Hδ: 3.702, 3.884
Gly7[d]	8.452	3.921, 3.968		
Orn8	8.209	4.350	1.777, 1.868	Hγ: 1.692, 1.742; Hδ: 3.009; NHt: 7.615
Orn9	8.472	4.356	1.756, 1.836	Hγ: 1.690; Hδ: 3.016; NHt: 7.615
Ile10	8.300	4.131	1.827	Hγ: 1.177, 1.470, 0.892 (Me); Hδ: 0.852
Leu11	8.393	4.374	1.589, 1.642	Hγ: 1.589; Hδ: 0.868, 0.928
Gln12	8.356	4.294	1.977, 2.102	Hγ: 2.360; HNt: 6.867, 7.527
NH_2	7.108, 7.599			

[a]The assignments for the minor Val3 spin system are 8.076(HN), 4.234(Hα), 2.178(Hβ), 0.925(Hγ). [b]The assignments for the minor Thr4 spin system are 8.257(HN), 4.314(Hα), 4.207(Hβ), 1.189(Hγ). [c]The assignments for the minor Val5 spin system are 7.968(NH), 4.268(Hα), 1.986(Hβ), 0.900(Hγ). [d]The assignments for the minor Gly7 spin system are 8.539(HN), 3.895, 3.971(Hα).

Table S12. The ^{1}H Chemical Shift Assignments for Peptide HPDUAspLys

Residue	HN	Hα	Hβ	Others
Ac-		2.041		
Arg1	8.296	4.267	1.731, 1.809	Hγ: 1.622; Hδ: 3.197; HNt: 7.207
Asp2	8.474	4.640	2.600, 2.726	
Val3[a]	8.042	4.190	2.150	Hγ: 0.922
Thr4	8.305	4.316	4.128	Hγ: 1.178
Val5[b]	8.195	4.429	2.079	Hγ: 0.958
^{L}Pro6		4.399	1.928, 2.308	Hγ: 1.990, 2.065; Hδ: 3.703, 3.885
Gly7[c]	8.452	3.922, 3.964		
Orn8	8.184	4.351	1.765, 1.868	Hγ: 1.699, 1.735; Hδ: 3.008; NHt: 7.552
Lys9	8.384	4.303	1.728, 1.772	Hγ: 1.371, 1.436; Hδ: 1.677; Hε: 2.986; NHt: 7.544
Ile10	8.272	4.130	1.833	Hγ: 1.187, 1.483, 0.887 (Me); Hδ: 0.851
Leu11	8.380	4.381	1.584, 1.646	Hγ: 1.584; Hδ: 0.865, 0.928
Gln12[d]	8.337	4.302	1.978, 2.106	Hγ: 2.361; HNt: 6.869, 7.531
NH_2	7.110, 7.599			

[a]The assignments for the minor Val3 spin system are 8.071(HN), 4.231(Hα), 2.168(Hβ), 0.924(Hγ). [b]The assignments for the minor Val5 spin system are 7.970(NH), 4.269(Hα), 1.987(Hβ), 0.901(Hγ). [c]The assignments for the minor Gly7 spin system are 8.536(HN), 3.896, 3.970(Hα). [d]The assignments for the minor Gln12 spin system are 8.258(HN), 4.316(Hα), 1.836, 2.059(Hβ).

Table S13. The ^{1}H Chemical Shift Assignments for Peptide HPDGluDap

Residue	HN	Hα	Hβ	Others
Ac-		2.024		
Arg1	8.253	4.322	1.731, 1.814	Hγ: 1.623; Hδ: 3.198; NHt: 7.202
Glu2	8.503	4.508	1.913, 1.969	Hγ: 2.161, 2.234
Val3[b]	8.518	4.303	2.073	Hγ: 0.909
Thr4[c]	8.420	4.743	4.029	Hγ: 1.076
Val5	8.459	4.557	2.006	Hγ: 0.929
^{D}Pro6		4.401	1.975, 2.337	Hγ: 2.044, 2.093; Hδ: 3.824, 3.863
Gly7[d]	8.400	3.833, 3.975		
Orn8[a]	8.157	4.542	1.829, 1.878	Hγ: 1.692,1.760 ; Hδ: 3.012
Dap9[a]	8.952	4.968	3.276, 3.384	
Ile10	8.654	4.293	1.858	Hγ: 1.129, 1.379, 0.871 (Me); Hδ: 0.812
Leu11[e]	8.449	4.479	1.594	Hγ: 1.594; Hδ: 0.851,0.900
Gln12[f]	8.537	4.332	1.966, 2.092	Hγ: 2.337; HNt: 6.865, 7.461
NH_2	7.129, 7.666			

[a]Signal for the terminal HN was not observed. [b]The assignments for the minor Val3 spin system are 8.263(HN), 4.165(Hα), 2.083(Hβ), 0.924(Hγ); [c]The assignments for the minor Thr4 spin system are 8.205(HN), 4.292 (Hα), 4.097(Hβ), 1.154(Hγ); [d]The assignments for the minor Gly7 spin system are 8.703(HN), 4.000(Hα). [e]The assignments for the minor Leu11 spin system are 8.340(HN), 4.107 (Hα), 2.024(Hβ), 0.802, 0.900(Hδ); [f]The assignments for the minor Gln12 spin system are 8.390(HN), 4.294 (Hα), 2.091, 2.113(Hβ), 2.366 (Hγ).

Table S14. The ^{1}H Chemical Shift Assignments for Peptide HPDGluDab

Residue	HN	Hα	Hβ	Others
Ac-		2.025		
Arg1	8.254	4.325	1.736, 1.810	Hγ: 1.624; Hδ: 3.199; NHt:7.201
Glu2	8.489	4.577	1.907, 1.976	Hγ: 2.162, 2.230
Val3[b]	8.542	4.333	1.954, 2.089	Hγ: 0.869, 0.847
Thr4[c]	8.372	4.793	3.976	Hγ: 1.037
Val5	8.587	4.576	1.991	Hγ: 0.929
^{D}Pr6[d]		4.388	1.976, 2.343	Hγ: 2.045, 2.097; Hδ: 3.825, 3.874
Gly7[e]	8.401	3.785, 3.990		
Orn8	8.039	4.552	1.810, 1.859	Hγ: 1.732 ; Hδ: 3.017; NHt: 7.619
Dab9[a]	8.268	4.720	2.056	Hγ: 2.970, 3.041
Ile10[f]	8.665	4.296	1.842	Hγ: 1.132, 1.380, 0.871 (Me); Hδ: 0.803
Leu11	8.428	4.502	1.597	Hγ: 1.597; Hδ: 0.852, 0.901
Gln12[g]	8.538	4.336	1.964, 2.088	Hγ: 2.329; HNt: 7.453, 6.865
NH_2	7.130, 7.672			

[a]Signal for the terminal HN was not observed. [b]The assignments for the minor Val3 spin system are 8.263(HN), 4.163(Hα), 2.085(Hβ), 0.929(Hγ). [c]The assignments for the minor Thr4 spin system are 8.220(HN), 4.290(Hα), 4.099(Hβ), 1.160(Hγ). [d]The assignments for the minor Pro6 spin system are 4.875(Hα), 2.300, 1.923(Hβ), 2.133(Hγ), 3.473, 3.589(Hδ). [e]The assignments for the minor Gly7 spin system are 8.679(HN), 4.001, 3.961(Hα). [f]The assignments for the minor Ile10 spin system are 8.330(HN), 4.119(Hα), 1.835(Hβ), 0.895(Hγ), 0.802(Hδ). [g]The assignments for the minor Gln12 spin system are 8.372(HN), 4.229(Hα), 1.976, 2.103(Hβ), 2.362(Hγ).

Table S15. The ^{1}H Chemical Shift Assignments for Peptide HPDGluOrn

Residue	HN	Hα	Hβ	Others
Ac-		2.028		
Arg1	8.258	4.316	1.732, 1.813	Hγ: 1.620; Hδ: 3.199; HNt: 7.202
Glu2[b]	8.499	4.532	1.941	Hγ: 2.154, 2.231
Val3[c]	8.521	4.304	2.070	Hγ: 0.907
Thr4[d]	8.311	4.758	3.972	Hγ: 1.048
Val5[e]	8.606	4.568	1.991	Hγ: 0.929
^{D}Pro6		4.391	1.972, 2.346	Hγ: 2.046, 2.088; Hδ: 3.822, 3.880
Gly7[f]	8.393	3.781, 3.989		
Orn8	8.022	4.534	1.809, 1.859	Hγ: 1.686, 1.720; Hδ: 3.016; NHt: 7.617
Orn9[a]	8.573	4.560	1.742	Hγ: 1.637; Hδ: 2.971
Ile10	8.598	4.271	1.838	Hγ: 1.150, 1.394, 0.867 (Me); Hδ: 0.803
Leu11	8.406	4.472	1.622	Hγ: 1.580; Hδ: 0.852, 0.906
Gln12[g]	8.508	4.322	2.076, 2.105	Hγ: 2.337; HNt: 6.871, 7.467
NH_2	7.124, 7.658			

[a]Signal for the terminal HN was not observed. [b]The assignments for the minor Glu2 spin system are 8.480(HN), 4.349(Hα), 1.757(Hβ), 1.833, 1.928(Hγ). [c]The assignments for the minor Val3 spin system are 8.264(HN), 4.162(Hα), 2.089(Hβ), 0.927(Hγ). [d]The assignments for the minor Thr4 spin system are 8.218(HN), 4.289(Hα), 4.104(Hβ), 1.158(Hγ). [e]The assignments for the minor Val5 spin system are 8.338(HN), 4.102(Hα), 2.035(Hβ), 0.799, 0.899(Hγ). [f]The assignments for the minor Gly7 spin system are 8.685(HN), 3.945, 4.000(Hα). [g]The assignments for the minor Gln12 spin system are 8.360(HN), 4.297(Hα), 1.977, 2.101(Hβ), 2.360(Hγ).

Table S16. The ^{1}H Chemical Shift Assignments for Peptide HPDGluLys

Residue	HN	Hα	Hβ	Others
Ac-		2.029		
Arg1	8.273	4.318	1.730, 1.810	Hγ: 1.618; Hδ: 3.201; HNt: 7.211
Glu2	8.523	4.519	1.910, 1.962	Hγ: 2.161, 2.244
Val3[a]	8.530	4.296	2.074	Hγ: 0.907
Thr4[b]	8.306	4.743	3.968	Hγ: 1.049
Val5[c]	8.626	4.559	1.994	Hγ: 0.930
^{D}Pro6		4.389	1.972, 2.345	Hγ: 2.042, 2.090; Hδ: 3.822, 3.878
Gly7[d]	8.398	3.784		
Orn8	8.008	4.531	1.801, 1.865	Hγ: 1.699; Hδ: 3.016; NHt: 7.620
Lys9	8.503	4.528	1.700	Hγ: 1.322, 1.418; Hδ: 1.625; Hε: 2.936; NHt: 7.552
Ile10	8.604	4.260	1.839	Hγ: 1.157, 1.403, 0.865 (Me); Hδ: 0.801
Leu11	8.406	4.483	1.571, 1.629	Hγ: 1.602; Hδ: 0.850, 0.904
Gln12[e]	8.516	4.328	1.959, 2.092	Hγ: 2.344; HNt: 6.882, 7.483
NH_2	7.135, 7.673			

[a]The assignments for the minor Val3 spin system are 8.301(HN), 4.129(Hα), 1.823(Hβ), 0.877(Hγ). [b]The assignments for the minor Thr4 spin system are 8.238(HN), 4.292(Hα), 4.104(Hβ), 1.158(Hγ). [c]The assignments for the minor Val5 spin system are 8.282(HN), 4.160(Hα), 2.086(Hβ), 0.928(Hγ). [d]The assignments for the minor Gly7 spin system are 8.691(HN), 3.935, 3.998(Hα). [e]The assignments for the minor Gln12 spin system are 8.358(HN), 4.296(Hα), 1.976, 2.100(Hβ), 2.360(Hγ).

Table S17. The ^{1}H Chemical Shift Assignments for Peptide HPDFGluDap

Residue	HN	Hα	Hβ	Others
Ac-		2.075		
Cys1	8.450	5.209	2.603, 3.170	
Arg2	8.734	4.617	1.811	Hγ: 1.556, 1.654; Hδ: 3.180; NHt: 7.140
Glu3	8.705	4.871	1.860, 1.903	Hγ: 2.065, 2.163
Val4	9.052	4.520	2.026	Hγ: 0.828, 0.872
Thr5	8.656	5.111	3.972	Hγ: 1.028
Val6	8.685	4.637	1.948	Hγ: 0.891, 0.921
^{D}Pro7		4.353	1.967, 2.369	Hγ: 2.045, 2.153; Hδ: 3.815, 3.883
Gly8	8.509	3.786, 3.991		
Orn9	8.050	4.705	1.854	Hγ: 1.679,1.742 ; Hδ: 3.013; NHt: 7.630
Dap10[a]	9.018	5.292	3.268	
Ile11	9.169	4.519	1.860	Hγ: 1.077, 1.312, 0.852 (Me); Hδ: 0.794
Leu12	8.441	4.745	1.561	Hγ: 1.478; Hδ: 0.798
Gln13	9.135	4.656	1.899, 2.099	Hγ: 2.222, 2.275; HNt: 6.827, 7.355
Cys14	9.013	5.087	3.004, 3.131	
NH_2	7.238, 7.600			

[a]Signal for the terminal HN was not observed.

Table S18. The ^{1}H Chemical Shift Assignments for Peptide HPDFGluDab

Residue	HN	Hα	Hβ	Others
Ac-		2.073		
Cys1	8.448	5.192	2.610, 3.176	
Arg2	8.716	4.608	1.817	Hγ: 1.562, 1.652; Hδ: 3.185; NHt: 7.146
Glu3	8.712	4.951	1.877	Hγ: 2.033, 2.150
Val4	8.999	4.565	2.052	Hγ: 0.810, 0.870
Thr5	8.537	5.065	3.940	Hγ: 0.987
Val6	8.835	4.633	1.936	Hγ: 0.889, 0.918
^{D}Pro7		4.346	1.964, 2.375	Hγ: 2.043, 2.150; Hδ: 3.833, 3.876
Gly8	8.458	3.725, 4.008		
Orn9	7.974	4.680	1.828	Hγ: 1.661, 1.710; Hδ: 3.005; NHt: 7.607
Dab10[a]	8.771	5.019	1.974, 2.029	Hγ: 2.884, 2.993
Ile11	9.166	4.512	1.827	Hγ: 1.051, 1.300, 0.841 (Me); Hδ: 0.786
Leu12	8.419	4.772	1.562	Hγ: 1.499; Hδ: 0.801
Gln13	9.131	4.642	1.912, 2.096	Hγ: 2.219, 2.267; HNt: 6.822, 7.353
Cys14	9.006	5.083	3.002, 3.130	
NH_2	7.240, 7.607			

[a]Signal for the terminal HN was not observed.

Table S19. The ^{1}H Chemical Shift Assignments for Peptide HPDFGluOrn

Residue	HN	Hα	Hβ	Others
Ac-		2.076		
Cys1	8.448	5.203	2.615, 3.169	
Arg2	8.724	4.616	1.817	Hγ: 1.553, 1.654; Hδ: 3.182; HNt: 7.137
Glu3	8.705	4.947	1.866	Hγ: 1.993, 2.153
Val4	9.042	4.534	2.013, 2.048	Hγ: 0.816, 0.870
Thr5	8.513	5.045	3.954	Hγ: 1.010
Val6	8.906	4.625	1.923, 1.961	Hγ: 0.887, 0.918
^{D}Pro7		4.351	1.963, 2.369	Hγ: 2.045, 2.152; Hδ: 3.820, 3.880
Gly8	8.450	3.746, 3.998		
Orn9	7.977	4.664	1.833	Hγ: 1.688; Hδ: 3.006; NHt: 7.620
Orn10[a]	8.579	4.898	1.729	Hγ: 1.571, 1.661; Hδ: 2.886, 2.915
Ile11	9.143	4.500	1.823	Hγ: 1.074, 1.325, 0.841 (Me); Hδ: 0.791
Leu12	8.417	4.740	1.494, 1.570	Hγ: 1.494; Hδ: 0.807
Gln13	9.127	4.648	1.905, 2.098	Hγ: 2.224, 2.272; HNt: 6.825, 7.347
Cys14	9.005	5.085	3.003, 3.129	
NH_2	7.240, 7.602			

[a]Signal for the terminal HN was not observed.

Table S20. The ^{1}H Chemical Shift Assignments for Peptide HPDFGluLys

Residue	HN	Hα	Hβ	Others
Ac-		2.076		
Cys1	8.447	5.193	2.610, 3.171	
Arg2	8.718	4.614	1.815	Hγ: 1.559, 1.656; Hδ: 3.185; HNt: 7.138
Glu3	8.696	4.932	1.838, 1.892	Hγ: 1.992, 2.172
Val4	9.051	4.516	2.029	Hγ: 0.820, 0.869
Thr5	8.486	5.008	3.947	Hγ: 1.006
Val6	8.930	4.614	1.943	Hγ: 0.888, 0.917
^{D}Pro7		4.352	1.962, 2.368	Hγ: 2.043, 2.152; Hδ: 3.822, 3.877
Gly8	8.435	3.741, 3.996		
Orn9	7.950	4.670	1.826	Hγ: 1.686; Hδ: 3.009; NHt: 7.619
Lys10[a]	8.496	4.855	1.681	Hγ: 1.254, 1.369; Hδ: 1.568; Hε: 2.873
Ile11	9.167	4.489	1.830	Hγ: 1.096, 1.332, 0.845 (Me); Hδ: 0.791
Leu12	8.392	4.739	1.501, 1.571	Hγ: 1.501; Hδ: 0.801, 0.818
Gln13	9.133	4.650	1.908, 2.097	Hγ: 2.223, 2.272; HNt: 6.825, 7.338
Cys14	9.015	5.084	3.002, 3.131	
NH_2	7.238, 7.605			

[a]Signal for the terminal HN was not observed.

Table S21. The ^{1}H Chemical Shift Assignments for Peptide HPDUGluDap

Residue	HN	Hα	Hβ	Others
Ac-		2.034		
Arg1	8.273	4.274	1.734, 1.810	Hγ: 1.633; Hδ: 3.198; NHt: 7.212
Glu2	8.537	4.332	1.939, 2.035	Hγ: 2.261
Val3	8.254	4.166	2.078	Hγ: 0.929
Thr4[b]	8.263	4.352	4.107	Hγ: 1.164
Val5[c]	8.273	4.440	2.073	Hγ: 0.939, 0.968
^{L}Pro6		4.401	1.932, 2.308	Hγ: 1.990, 2.063; Hδ: 3.704, 3.882
Gly7[d]	8.469	3.971		
Orn8[a]	8.312	4.401	1.912	Hγ: 1.701, 1.770; Hδ: 3.008
Dap9[a]	8.828	4.743	3.281, 3.472	
Ile10	8.322	4.156	1.848	Hγ: 1.154, 1.428, 0.891 (Me); Hδ: 0.850
Leu11	8.449	4.386	1.614	Hγ: 1.614; Hδ: 0.871, 0.929
Gln12[e]	8.400	4.294	1.917, 2.103	Hγ: 2.362; HNt: 6.865, 7.520
NH_2	7.109, 7.599			

[a]Signal for the terminal HN was not observed. [b]The assignments for the minor Thr4 spin system are 8.200(HN), 4.352(Hα), 4.157(Hβ), 1.183(Hγ). [c]The assignments for the minor Val5 spin system are 8.000(HN), 4.264(Hα), 1.986(Hβ), 0.890(Hγ). [d]The assignments for the minor Gly7 spin system are 8.547(HN), 3.907, 3.990(Hα). [e]The assignments for the minor Gln12 spin system are 8.400(HN), 4.161(Hα), 1.917(Hβ), 2.274(Hγ).

Table S22. The ^{1}H Chemical Shift Assignments for Peptide HPDUGluDab

Residue	HN	Hα	Hβ	Others
Ac-		2.039		
Arg1	8.272	4.271	1.735, 1.818	Hγ: 1.633; Hδ: 3.202; NHt: 7.214
Glu2	8.537	4.337	1.941, 2.037	Hγ: 2.265
Val3[b]	8.253	4.165	2.080	Hγ: 0.932
Thr4[c]	8.267	4.365	4.107	Hγ: 1.163
Val5	8.280	4.446	2.068	Hγ: 0.938, 0.971
Pr6[d]		4.400	1.934, 2.313	Hγ: 1.989, 2.063; Hδ: 3.706, 3.882
Gly7[e]	8.458	3.955		
Orn8	8.241	4.357	1.874	Hγ: 1.639, 1.767; Hδ: 3.011
Dab9[a]	8.614	4.469	2.065, 2.151	Hγ: 3.061
Ile10[f]	8.326	4.137	1.837	Hγ: 1.182, 1.462, 0.896 (Me); Hδ: 0.855
Leu11	8.429	4.386	1.617	Hγ: 1.617; Hδ: 0.870, 0.929
Gln12[g]	8.374	4.302	1.983, 2.101	Hγ: 2.362; NHt: 6.873, 7.529
NH_2	7.109, 7.605			

[a]Signal for the terminal HN was not observed. [b]The assignments for the minor Thr4 spin system are 8.207(HN), 4.353(Hα), 4.161(Hβ), 1.183(Hγ). [c]The assignments for the minor Val5 spin system are 8.008(HN), 4.263(Hα), 1.985(Hβ), 0.900(Hγ). [d]The assignments for the minor Pro6 spin system are 4.720(Hα), 2.396,1.882 (Hβ), 1.972, 2.133(Hγ), 3.530, 3.611(Hδ). [e]The assignments for the minor Gly7 spin system are 8.543(HN), 3.885, 3.988(Hα).

Table S23. The ^{1}H Chemical Shift Assignments for Peptide HPDUGluOrn

Residue	HN	Hα	Hβ	Others
Ac-		2.039		
Arg1	8.272	4.272	1.733	Hγ: 1.631; Hδ: 3.202; HNt: 7.213
Glu2	8.536	4.341	1.941	Hγ: 2.037, 2.273
Val3[b]	8.254	4.167	2.078	Hγ: 0.934
Thr4	8.270	4.357	4.107	Hγ: 1.163
Val5	8.285	4.445	1.834	Hγ: 0.972
^{L}Pro6		4.401	1.933, 2.311	Hγ: 1.990, 2.064; Hδ: 3.707, 3.882
Gly7[c]	8.458	3.951		
Orn8	8.212	4.355	1.866	Hγ: 1.689, 1.770; Hδ: 3.011; NHt: 7.621
Orn9[a]	8.481	4.359	1.837	Hγ: 1.693, 1.756; Hδ: 3.019
Ile10	8.301	4.136	1.843	Hγ: 1.476, 1.185, 0.893 (Me); Hδ: 0.854
Leu11	8.397	4.380	1.644	Hγ: 1.591; Hδ: 0.869, 0.931
Gln12	8.362	4.298	1.979, 2.107	Hγ: 2.364; HNt: 6.870, 7.530
NH_2	7.112, 7.602			

[a]Signal for the terminal HN was not observed. [b]The assignments for the minor Val3 spin system are 8.017(HN), 4.267(Hα), 1.986(Hβ), 0.901(Hγ). [c]The assignments for the minor Gly7 spin system are 8.550(HN), 3.883, 3.987(Hα).

Table S24. The ^{1}H Chemical Shift Assignments for Peptide HPDUGluLys

Residue	HN	Hα	Hβ	Others
Ac-		2.039		
Arg1	8.287	4.269	1.730, 1.829	Hγ: 1.628; Hδ: 3.202; HNt: 7.211
Glu2	8.552	4.334	1.938, 2.032	Hγ: 2.243, 2.279
Val3[a]	8.273	4.162	2.079	Hγ: 0.907
Thr4	8.288	4.104	3.968	Hγ: 1.049
Val5	8.310	4.443	2.073	Hγ: 0.969
^{L}Pro6		4.398	1.922, 2.064	Hγ: 1.941, 1.989; Hδ: 3.706, 3.884
Gly7[b]	8.473	3.944		
Orn8	8.197	4.351	1.765, 1.862	Hγ: 1.688; Hδ: 3.016; NHt: 7.623
Lys9	8.407	4.303	1.772	Hγ: 1.373, 1.439; Hδ: 1.625; Hε: 2.984; NHt: 7.557
Ile10	8.298	4.131	1.839	Hγ: 1.157, 1.403, 0.865 (Me); Hδ: 0.801
Leu11	8.404	4.380	1.583, 1.649	Hγ: 1.602; Hδ: 0.850, 0.904
Gln12	8.359	4.296	1.959, 2.092	Hγ: 2.344; HNt: 6.880, 7.545
NH_2	7.123, 7.613			

[a]The assignments for the minor Val3 spin system are 8.036(HN), 4.263(Hα), 1.984(Hβ), 0.899(Hγ). [b]The assignments for the minor Gly7 spin system are 8.558(HN), 3.875, 3.981(Hα).

Table S25. The ^{1}H Chemical Shift Assignments for Peptide HPDAadDap

Residue	HN	Hα	Hβ	Others
Ac-		2.015		
Arg1	8.245	4.323	1.732, 1.800	Hγ: 1.614; Hδ: 3.199; NHt: 7.198
Aad2	8.411	4.509	1.698	Hγ: 1.478, 1.585; Hδ: 2.167
Val3[b]	8.523	4.304	2.045	Hγ: 0.901
Thr4[c]	8.401	4.739	4.030	Hγ: 1.076
Val5	8.449	4.568	2.006	Hγ: 0.930
^{D}Pro6		4.402	1.986, 2.329	Hγ: 2.045, 2.084; Hδ: 3.815, 3.863
Gly7[d]	8.411	3.839, 3.972		
Orn8[a]	8.166	4.558	1.830, 1.884	Hγ: 1.693, 1.756; Hδ: 3.013
Dap9[a]	8.959	4.979	3.287, 3.346	
Ile10	8.665	4.304	1.859	Hγ: 1.379, 1.120, 0.871 (Me); Hδ: 0.813
Leu11	8.440	4.499	1.595	Hγ: 1.595; Hδ: 0.852, 0.901
Gln12	8.567	4.333	1.970, 2.093	Hγ: 2.337; HNt: 6.866, 7.462
NH_2	7.130, 7.668			

[a]Signal for the terminal HN was not observed. [b]The assignments for the minor Val3 spin system are 8.264(HN), 4.157(Hα), 2.069(Hβ), 0.930(Hγ). [c]The assignments for the minor Thr4 spin system are 8.206(HN), 4.280(Hα), 4.098 (Hβ), 1.150(Hγ). [d]The assignments for the minor Gly7 spin system are 8.665(HN), 3.971, 4.010(Hα).

Table S26. The ^{1}H Chemical Shift Assignments for Peptide HPDAadDab

Residue	HN	Hα	Hβ	Others
Ac-		2.015		
Arg1	8.244	4.327	1.734, 1.807	Hγ: 1.619; Hδ: 3.198; NHt: 7.198
Aad2	8.401	4.543	1.679, 1.721	Hγ: 1.487, 1.585; Hδ: 2.166
Val3[b]	8.544	4.329	2.060	Hγ: 0.900
Thr4[c]	8.332	4.753	3.982	Hγ: 1.045
Val5	8.571	4.567	1.995	Hγ: 0.926
^{D}Pro6		4.391	1.975, 2.346	Hγ: 2.045, 2.092; Hδ: 3.824, 3.873
Gly7[d]	8.404	3.795, 3.980		
Orn8[a]	8.052	4.535	1.811, 1.856	Hγ: 1.682, 1.733; Hδ: 3.016
Dab9[a]	8.727	4.700	2.064	Hγ: 2.990, 3.042
Ile10	8.645	4.294	1.837	Hγ: 1.389, 1.129, 0.869 (Me); Hδ: 0.807
Leu11	8.416	4.513	1.577, 1.611	Hγ: 1.577; Hδ: 0.851, 0.900
Gln12[e]	8.558	4.338	1.968, 2.090	Hγ: 2.334; HNt: 6.865, 7.462
NH_2	7.129, 7.676			

[a]Signal for the terminal HN was not observed. [b]The assignments for the minor Val3 spin system are 8.260(HN), 4.157(Hα), 2.070(Hβ), 0.930(Hγ). [c]The assignments for the minor Thr4 spin system are 8.213(HN), 4.277(Hα), 4.096(Hβ), 1.155(Hγ). [d]The assignments for the minor Gly7 spin system are 8.650(HN), 3.951, 4.010(Hα). [e]The assignments for the minor Gln12 spin system are 8.372(HN), 4.300(Hα), 1.976, 2.098(Hβ), 2.363(Hγ).

Table S27. The ^{1}H Chemical Shift Assignments for Peptide HPDAadOrn

Residue	HN	Hα	Hβ	Others
Ac-		2.021		
Arg1[b]	8.247	4.318	1.733, 1.808	Hγ: 1.617; Hδ: 3.197; HNt: 7.201
Aad2	8.410	4.501	1.706	Hγ: 1.492; Hδ: 2.153, 2.192
Val3[c]	8.478	4.291	2.050	Hγ: 0.907
Thr4[d]	8.287	4.711	3.991	Hγ: 1.059
Val5[e]	8.573	4.560	2.000	Hγ: 0.928
^{D}Pro6		4.396	1.964, 2.338	Hγ: 1.985, 2.062; Hδ: 3.815, 3.873
Gly7[f]	8.386	3.805, 3.972		
Orn8	8.038	4.515	1.809, 1.864	Hγ: 1.688, 1.728; Hδ: 3.016; NHt: 7.618
Orn9[a]	8.566	4.554	1.835	Hγ: 1.663, 1.753; Hδ: 2.972
Ile10	8.557	4.259	1.834	Hγ: 1.147, 1.403, 0.869 (Me); Hδ: 0.808
Leu11	8.408	4.463	1.598, 1.697	Hγ: 1.488; Hδ: 0.851, 0.904
Gln12[g]	8.510	4.322	1.969, 2.092	Hγ: 2.341; HNt: 6.870, 7.476
NH_2	7.124, 7.652			

[a]Signal for the terminal HN was not observed. [b]The assignments for the minor Arg1 spin system are 8.381(HN), 4.359(Hα), 1.743, 1.866(Hβ), 1.589(Hγ). [c]The assignments for the minor Val3 spin system are 8.261(HN), 4.158(Hα), 2.070(Hβ), 0.923(Hγ). [d]The assignments for the minor Thr4 spin system are 8.217(HN), 4.280(Hα), 4.102(Hβ), 1.154(Hγ). [e]The assignments for the minor Val5 spin system are 8.327(HN), 4.108(Hα), 2.037(Hβ), 0.802, 0.896(Hγ). [f]The assignments for the minor Gly7 spin system are 8.650(HN), 3.945, 4.000(Hα). [g]The assignments for the minor Gln12 spin system are 8.359(HN), 4.297(Hα), 1.977, 2.102(Hβ), 2.360(Hγ).

Table S28. The ^{1}H Chemical Shift Assignments for Peptide HPDAadLys

Residue	HN	Hα	Hβ	Others
Ac-		2.021		
Arg1[a]	8.244	4.323	1.732, 1.811	Hγ: 1.618; Hδ: 3.197; HNt: 7.201
Aad2	8.410	4.519	1.695	Hγ: 1.488; Hδ: 2.146, 2.199
Val3[b]	8.498	4.293	2.053	Hγ: 0.906
Thr4	8.274	4.723	3.979	Hγ: 1.052
Val5[c]	8.607	4.555	2.012	Hγ: 0.929
^{D}Pro6		4.391	1.964, 2.339	Hγ: 1.984, 2.068; Hδ: 3.818, 3.871
Gly7[d]	8.383	3.796, 3.976		
Orn8	8.008	4.523	1.801, 1.865	Hγ: 1.684, 1.719; Hδ: 3.016; NHt: 7.614
Lys9	8.488	4.526	1.701	Hγ: 1.331, 1.430; Hδ: 1.638; Hε: 2.942; NHt: 7.609
Ile10	8.576	4.264	1.837	Hγ: 1.155, 1.402, 0.886 (Me); Hδ: 0.804
Leu11	8.399	4.468	1.600, 1.717	Hγ: 1.600; Hδ: 0.851, 0.901
Gln12[e]	8.503	4.321	1.961, 2.079	Hγ: 2.339; HNt: 6.870, 7.471
NH_2	7.123, 7.654			

[a]The assignments for the minor Arg1 spin system are 8.357(HN), 4.355(Hα), 1.737, 1.874(Hβ), 1.598(Hγ). [b]The assignments for the minor Val3 spin system are 8.260(HN), 4.160(Hα), 2.069(Hβ), 0.925(Hγ). [c]The assignments for the minor Val5 spin system are 8.323(HN), 4.110(Hα), 2.037(Hβ), 0.802, 0.897(Hγ). [d]The assignments for the minor Gly7 spin system are 8.641(HN), 3.940, 3.998(Hα). [e]The assignments for the minor Gln12 spin system are 8.337(HN), 4.298(Hα), 1.976, 2.104(Hβ), 2.361(Hγ).

Table S29. The [1]H Chemical Shift Assignments for Peptide HPDFAadDap

Residue	HN	Hα	Hβ	Others
Ac-		2.076		
Cys1	8.442	5.214	2.594, 3.171	
Arg2	8.744	4.618	1.802	Hγ: 1.561, 1.646; Hδ: 3.180; NHt: 7.141
Aad3	8.656	4.862	1.654	Hγ: 1.430, 1.518; Hδ: 2.109
Val4	9.018	4.525	2.017	Hγ: 0.824, 0.873
Thr5	8.657	5.097	3.963	Hγ: 1.029
Val6	8.671	4.637	1.954	Hγ: 0.892, 0.922
[D]Pro7		4.354	1.968, 2.369	Hγ: 2.046, 2.154; Hδ: 3.807, 3.875
Gly8	8.520	3.796, 3.992		
Orn9	8.040	4.716	1.856	Hγ: 1.684,1.743 ; Hδ: 3.015; NHt: 7.623
Dap10 [a]	9.038	5.332	3.180, 3.269	
Ile11	9.160	4.540	1.860	Hγ: 1.063, 1.303, 0.852 (Me); Hδ: 0.795
Leu12	8.451	4.774	1.528, 1.587	Hγ: 1.478; Hδ: 0.785,0.804
Gln13	9.175	4.657	1.899, 2.105	Hγ: 2.222, 2.271; HNt: 6.827, 7.366
Cys14	9.028	5.097	3.004, 3.132	
NH_2	7.243, 7.600			

[a]Signal for the terminal HN was not observed.

Table S30. The [1]H Chemical Shift Assignments for Peptide HPDFAadDab

Residue	HN	Hα	Hβ	Others
Ac-		2.074		
Cys1	8.445	5.203	2.587, 3.178	
Arg2	8.738	4.607	1.808	Hγ: 1.566, 1.648; Hδ: 3.178; NHt: 7.139
Aad3	8.634	4.906	1.623, 1.675	Hγ: 1.437, 1.516; Hδ: 2.082, 2.119
Val4	9.016	4.557	2.034	Hγ: 0.812, 0.861
Thr5	8.503	5.065	3.945	Hγ: 0.998
Val6	8.831	4.626	1.941	Hγ: 0.885, 0.919
[D]Pro7		4.349	1.961, 2.370	Hγ: 2.044, 2.152; Hδ: 3.824, 3.873
Gly8	8.478	3.745, 4.000		
Orn9	7.989	4.675	1.830	Hγ: 1.690; Hδ: 3.003; NHt: 7.623
Dab10[a]	8.748	5.016	1.966, 2.025	
Ile11	9.164	4.518	1.824	Hγ: 1.042, 1.301, 0.836 (Me); Hδ: 0.787
Leu12	8.391	4.806	1.535, 1.584	Hγ: 1.483; Hδ: 0.793, 0.812
Gln13	9.172	4.643	1.906, 2.112	Hγ: 2.220, 2.266; HNt: 6.816, 7.364
Cys14	9.023	5.091	3.003, 3.130	
NH_2	7.246, 7.607			

[a]Signal for the terminal HN was not observed.

Table S31. The ^{1}H Chemical Shift Assignments for Peptide HPDFAadOrn

Residue	HN	Hα	Hβ	Others
Ac-		2.078		
Cys1	8.449	5.210	2.610, 3.169	
Arg2	8.740	4.618	1.802	Hγ: 1.553, 1.650; Hδ: 3.180; HNt: 7.134
Aad3	8.645	4.904	1.591, 1.666	Hγ: 1.430, 1.542; Hδ: 2.071, 2.115
Val4	9.009	4.520	2.031	Hγ: 0.823, 0.868
Thr5	8.518	4.998	3.957	Hγ: 1.022
Val6	8.911	4.613	1.939	Hγ: 0.886, 0.916
^{D}Pro7		4.353	1.963, 2.365	Hγ: 2.043, 2.153; Hδ: 3.810, 3.878
Gly8	8.461	3.768, 3.984		
Orn9	7.979	4.674	1.835	Hγ: 1.691; Hδ: 3.009; NHt: 7.620
Orn10[a]	8.586	4.944	1.771	Hγ: 1.618, 1.684; Hδ: 2.875, 2.918
Ile11	9.115	4.512	1.823	Hγ: 1.071, 1.322, 0.846 (Me); Hδ: 0.793
Leu12	8.420	4.737	1.632	Hγ: 1.495; Hδ: 0.784, 0.818
Gln13	9.161	4.648	1.898, 2.108	Hγ: 2.223, 2.276; HNt: 6.827, 7.356
Cys14	9.012	5.087	3.003, 3.128	
NH_2	7.241, 7.600			

[a]Signal for the terminal HN was not observed.

Table S32. The ^{1}H Chemical Shift Assignments for Peptide HPDFAadLys

Residue	HN	Hα	Hβ	Others
Ac-		2.076		
Cys1	8.447	5.213	2.615, 3.180	
Arg2	8.738	4.614	1.805	Hγ: 1.550, 1.650; Hδ: 3.180; HNt: 7.134
Aad3	8.648	4.920	1.554, 1.680	Hγ: 1.429; Hδ: 2.067, 2.129
Val4	9.031	4.504	2.024	Hγ: 0.827, 0.869
Thr5	8.492	4.959	3.956	Hγ: 1.017
Val6	8.941	4.601	1.941	Hγ: 0.887, 0.916
^{D}Pro7		4.351	1.960, 2.363	Hγ: 2.044, 2.150; Hδ: 3.807, 3.877
Gly8	8.440	3.758, 3.990		
Orn9	7.951	4.675	1.826	Hγ: 1.686; Hδ: 3.009; NHt: 7.619
Lys10[a]	8.504	4.884	1.716	Hγ: 1.286, 1.393; Hδ: 1.552; Hε: 2.881
Ile11	9.150	4.501	1.826	Hγ: 1.095, 1.337, 0.848 (Me); Hδ: 0.794
Leu12	8.420	4.725	1.636	Hγ: 1.496; Hδ: 0.784, 0.817
Gln13	9.158	4.656	1.900, 2.113	Hγ: 2.222, 2.279; HNt: 6.829, 7.342
Cys14	9.005	5.089	3.003, 3.130	
NH_2	7.241, 7.601			

[a]Signal for the terminal HN was not observed.

Table S33. The ^{1}H Chemical Shift Assignments for Peptide HPDUAadDap

Residue	HN	Hα	Hβ	Others
Ac-		1.939		
Arg1	8.168	4.193	1.649, 1.714	Hγ: 1.537, 1.544; Hδ: 3.112; NHt: 7.121
Aad2	8.324	4.247	1.625, 1.675	Hγ: 2.261; Hδ: 2.134
Val3	8.177	4.080	1.978	Hγ: 0.843
Thr4[b]	8.158	4.266	4.012	Hγ: 1.069
Val5[c]	8.187	4.364	1.988	Hγ: 0.853, 0.883
^{L}Pro6		4.315	1.840, 2.222	Hγ: 1.900, 1.978; Hδ: 3.625, 3.796
Gly7[d]	8.383	3.885		
Orn8[a, e]	8.226	4.320	1.826	Hγ: 1.606, 1.685; Hδ: 2.922
Dap9[a]	8.745	4.667	3.200, 3.386	
Ile10	8.236	4.070	1.758	Hγ: 1.059, 1.342, 0.805 (Me); Hδ: 0.765
Leu11	8.363	4.305	1.529	Hγ: 1.529; Hδ: 0.785, 0.844
Gln12	8.314	4.217	1.890, 2.017	Hγ: 2.277; HNt: 6.779, 7.435
NH_2	7.024, 7.513			

[a]Signal for the terminal HN was not observed. [b]The assignments for the minor Thr4 spin system are 8.099(HN), 4.257 (Hα), 4.071(Hβ), 1.074(Hγ). [c]The assignments for the minor Val5 spin system are 7.894(HN), 4.188(Hα), 1.890(Hβ), 0.805(Hγ). [d]The assignments for the minor Gly7 spin system are 8.471(HN), 3.824, 3.914(Hα). [e]The assignments for the minor Orn8 spin system are 8.432(HN), 4.291(Hα),1.814(Hβ), 1.616, 1.675(Hγ), 2.927(Hδ).

Table S34. The ^{1}H Chemical Shift Assignments for Peptide HPDUAadDab

Residue	HN	Hα	Hβ	Others
Ac-		2.025		
Arg1	8.253	4.279	1.740, 1.799	Hγ: 1.624; Hδ: 3.199; NHt: 7.207
Aad2	8.410	4.337	1.710, 1.764	Hγ: 1.528, 1.622
Val3[c]	8.263	4.165	2.063	Hγ: 0.929
Thr4[d]	8.250	4.362	4.097	Hγ: 1.154
Val5	8.283	4.449	2.064	Hγ: 0.939, 0.969
^{L}Pro6		4.400	1.936, 2.308	Hγ: 1.986, 2.063; Hδ: 3.707, 3.881
Gly7	8.458	3.954		
Orn8[a]	8.248	4.352	1.873	Hγ: 1.692, 1.760 ; Hδ: 3.012
Dab9[a]	8.615	4.469	2.161, 2.142	Hγ: 3.047, 3.075
Ile10	8.326	4.136	1.834	Hγ: 1.178, 1.467, 0.894 (Me); Hδ: 0.855
Leu11	8.429	4.389	1.594, 1.624	Hγ: 1.594, 1.624; Hδ: 0.871, 0.929
Gln12	8.375	4.302	1.983, 2.103	Hγ: 2.329; HNt: 6.874, 7.528
NH_2	7.109, 7.607			

[a]Signal for the terminal HN was not observed. [b]The assignments for the minor Val3 spin system are 7.995(HN), 4.269(Hα), 1.995(Hβ), 0.900(Hγ). [c]The assignments for the minor Thr4 spin system are 8.200(HN), 4.352 (Hα), 4.107(Hβ), 1.154(Hγ). [d]The assignments for the minor Pro6 spin system are 1.887, 2.396(Hβ), 1.971 ,2.162(Hγ). [e]The assignments for the minor Gly7 spin system are 8.552(HN), 3.885, 3.990(Hα).

Table S35. The ^{1}H Chemical Shift Assignments for Peptide HPDUAadOrn

Residue	HN	Hα	Hβ	Others
Ac-		2.026		
Arg1	8.251	4.279	1.731, 1.806	Hγ: 1.626; Hδ: 3.198; HNt: 7.207
Aad2	8.405	4.335	1.708, 1.767	Hγ: 1.528; Hδ: 2.215
Val3[a]	8.262	4.162	2.054	Hγ: 0.921
Thr4	8.250	4.351	4.096	Hγ: 1.154
Val5	8.284	4.444	2.081	Hγ: 0.971
^{L}Pro6		4.400	1.928, 2.308	Hγ: 1.988, 2.064; Hδ: 3.707, 3.881
Gly7[b]	8.458	3.931, 3.968		
Orn8	8.208	4.352	1.767, 1.865	Hγ: 1.684; Hδ: 3.011; NHt: 7.598
Orn9	8.481	4.358	1.756, 1.836	Hγ: 1.690; Hδ: 3.016; NHt: 7.632
Ile10	8.302	4.135	1.834	Hγ: 1.187, 1.471, 0.892 (Me); Hδ: 0.852
Leu11	8.399	4.378	1.591, 1.641	Hγ: 1.591; Hδ: 0.869, 0.929
Gln12	8.360	4.297	1.979, 2.102	Hγ: 2.361; HNt: 6.868, 7.529
NH_2	7.109, 7.602			

[a]The assignments for the minor Val3 spin system are 7.999(HN), 4.267(Hα), 1.983(Hβ), 0.897(Hγ). [b]The assignments for the minor Gly7 spin system are 8.556(HN), 3.882, 3.985(Hα).

Table S36. The ^{1}H Chemical Shift Assignments for Peptide HPDUAadLys

Residue	HN	Hα	Hβ	Others
Ac-		2.027		
Arg1	8.249	4.279	1.733, 1.805	Hγ: 1.625; Hδ: 3.198; HNt: 7.204
Aad2	8.404	4.336	1.711, 1.766	Hγ: 1.530, 1.622; Hδ: 2.216
Val3[a]	8.266	4.161	2.057	Hγ: 0.922
Thr4[b]	8.249	4.351	4.095	Hγ: 1.154
Val5	8.287	4.445	2.083	Hγ: 0.937
^{L}Pro6		4.398	1.929, 2.308	Hγ: 1.987, 2.065; Hδ: 3.706, 3.881
Gly7[c]	8.457	3.915, 3.964		
Orn8	8.181	4.350	1.762, 1.863	Hγ: 1.683, 1.708; Hδ: 3.010; NHt: 7.618
Lys9	8.392	4.306	1.721, 1.771	Hγ: 1.370, 1.439; Hδ: 1.653; Hε: 2.986; NHt: 7.548
Ile10	8.276	4.132	1.834	Hγ: 1.185, 1.483, 0.886 (Me); Hδ: 0.849
Leu11	8.385	4.381	1.583, 1.644	Hγ: 1.583; Hδ: 0.863, 0.928
Gln12	8.340	4.298	1.979, 2.103	Hγ: 2.362; HNt: 6.868, 7.531
NH_2	7.110, 7.602			

[a]The assignments for the minor Val3 spin system are 8.002(HN), 4.266(Hα), 1.984(Hβ), 0.898(Hγ). [b]The assignments for the minor Thr4 spin system are 8.206(NH), 4.348(Hα), 4.158(Hβ), 1.167(Hγ). [c]The assignments for the minor Gly7 spin system are 8.549(HN), 3.880, 3.980(Hα).

Table S37. The $^3J_{NH\alpha}$ (Hz) Values of the HPDAspXaa Peptides

	Xaa			
Residue	Dap	Dab	Orn	Lys
Arg1	8.8	8.8	9.8	9.8
**Asp2**	9.9	7.6	9.8	9.8
Val3	11	11	11	11
Thr4	11	11	11	10
Val5	11	11	11	9.2
Gly7	9.0	11	9.0	7.7
Orn8	9.8	7.6	9.8	11
**Xaa9**	10	8.4	9.9	9.9
Ile10	8.1	9.3	12	8.9
Leu11	9.9	8.1	9.9	11
Gln12	8.8	11	8.4	11

Table S38. The $^3J_{NH\alpha}$ (Hz) Values of the HPDGluXaa Peptides

	Xaa			
Residue	Dap	Dab	Orn	Lys
Arg1	9.9	6.5	10	10
**Glu2**	9.8	8.8	9.8	10
Val3	12	13	11	11
Thr4	11	12	10	10
Val5	9.4	9.9	10	9.6
Gly7	9.2	6.8	7.9	8.1
Orn8	11	11	11	11
**Xaa9**	9	7.3	11	8.4
Ile10	11	9.9	11	8.8
Leu11	11	7.6	9.8	8.8
Gln12	7.7	9.4	9.8	8.8

Table S39. The $^{3}J_{NH\alpha}$ (Hz) Values of the HPDAadXaa Peptides

	Xaa			
Residue	Dap	Dab	Orn	Lys
Arg1	8.8	9.9	10	10
**Aad2**	11	8.4	9.9	10
Val3	9.4	8.8	11	11
Thr4	9.4	9.6	10	10
Val5	9.6	11	12	9.8
Gly7	6.9	9.2	9.0	7.9
Orn8	8.4	9.4	9.9	9.8
**Xaa9**	13	12	10	9.8
Ile10	8.9	9.6	10	8.8
Leu11	8.8	9.6	9.9	9.8
Gln12	9.8	5.7	10	10

Table S40. The $^{3}J_{NH\alpha}$ (Hz) Values of the HPDFAspXaa Peptides

	Xaa			
Residue	Dap	Dab	Orn	Lys
Cys	11	11	11	12
Arg1	9.8	12	12	12
**Asp2**	9.6	9.9	11	11
Val3	11	12	13	13
Thr4	11	13	11	12
Val5	10	7.7	11	12
Gly7	9.0	11	9.8	11
Orn8	10	8.8	12	13
**Xaa9**	11	8.7	11	12
Ile10	10	12	12	13
Leu11	11	11	11	12
Gln12	12	11	11	12
Cys	11	12	12	12

Table S41. The $^{3}J_{NH\alpha}$ (Hz) Values of the HPDFGluXaa Peptides

	Xaa			
Residue	Dap	Dab	Orn	Lys
Cys	9.8	9.0	11	9.8
Arg1	9.5	9.0	11	9.6
**Glu2**	11	9.8	11	12
Val3	11	10	11	11
Thr4	11	8.4	10	11
Val5	9.6	9.4	11	11
Gly7	9.2	14	9.6	9.0
Orn8	11	12	11	10
**Xaa9**	9.6	9.6	11	9.6
Ile10	11	10	11	11
Leu11	11	9.9	9.6	13
Gln12	8.1	12	9.4	12
Cys	10	11	11	12

Table S42. The $^{3}J_{NH\alpha}$ (Hz) Values of the HPDFAadXaa Peptides

	Xaa			
Residue	Dap	Dab	Orn	Lys
Cys	11	8.6	12	11
Arg1	9.6	11	11	12
**Aad2**	9.8	8.6	11	11
Val3	11	11	11	11
Thr4	11	11	12	11
Val5	12	9.6	11	12
Gly7	9.3	7.6	9.6	11
Orn8	12	11	11	12
**Xaa9**	11	9.8	12	11
Ile10	9.6	9.2	13	12
Leu11	11	8.9	12	11
Gln12	12	11	12	11
Cys	10	10	12	11

Table S43. The $^3J_{NH\alpha}$ (Hz) Values of the HPDUAspXaa Peptides

	Xaa			
Residue	Dap	Dab	Orn	Lys
Arg1	10	9.5	9.4	9.9
**Asp2**	9.6	6.5	8.8	9.9
Val3	8.6	6.8	9.6	11
Thr4	9.9	12	9.8	11
Val5	8.6	7.3	11	9.2
Gly7	11	9.2	9.9	16
Orn8	9.8	8.6	9.2	11
**Xaa9**	12	8.4	9.8	9.9
Ile10	12	12	11	8.2
Leu11	9.8	6.1	9.9	9.4
Gln12	9.9	6.1	8.6	11

Table S44. The $^3J_{NH\alpha}$ (Hz) Values of the HPDUGluXaa Peptides

	Xaa			
Residue	Dap	Dab	Orn	Lys
Arg1	7.9	10	9.8	9.8
**Glu2**	7.9	8.4	11	9.2
Val3	8.8	7.6	9.8	9.3
Thr4	11	11	11	8.8
Val5	8.8	8.6	9.8	11
Gly7	13	13	12	8.9
Orn8	9.8	6.5	11	8.6
**Xaa9**	12	9.6	9.8	9.6
Ile10	10	12	9.8	7.3
Leu11	9.9	9.0	11	8.6
Gln12	9.9	8.8	11	9.8

Table S45. The $^{3}J_{NH\alpha}$ (Hz) Values of the HPDUAadXaa Peptides

	Xaa			
Residue	Dap	Dab	Orn	Lys
Arg1	7.6	11	8.8	9.9
**Aad2**	9.9	8.6	8.8	9.9
Val3	11	9.9	9.0	9.2
Thr4	11	11	9.8	9.8
Val5	9.6	9.8	9.9	9.8
Gly7	12	9.9	13	12
Orn8	9.8	9.8	8.6	9.8
**Xaa9**	11	11	9.8	8.8
Ile10	9.4	8.1	9.2	8.9
Leu11	9.9	9.8	9.8	11
Gln12	10	8.8	9.0	8.6

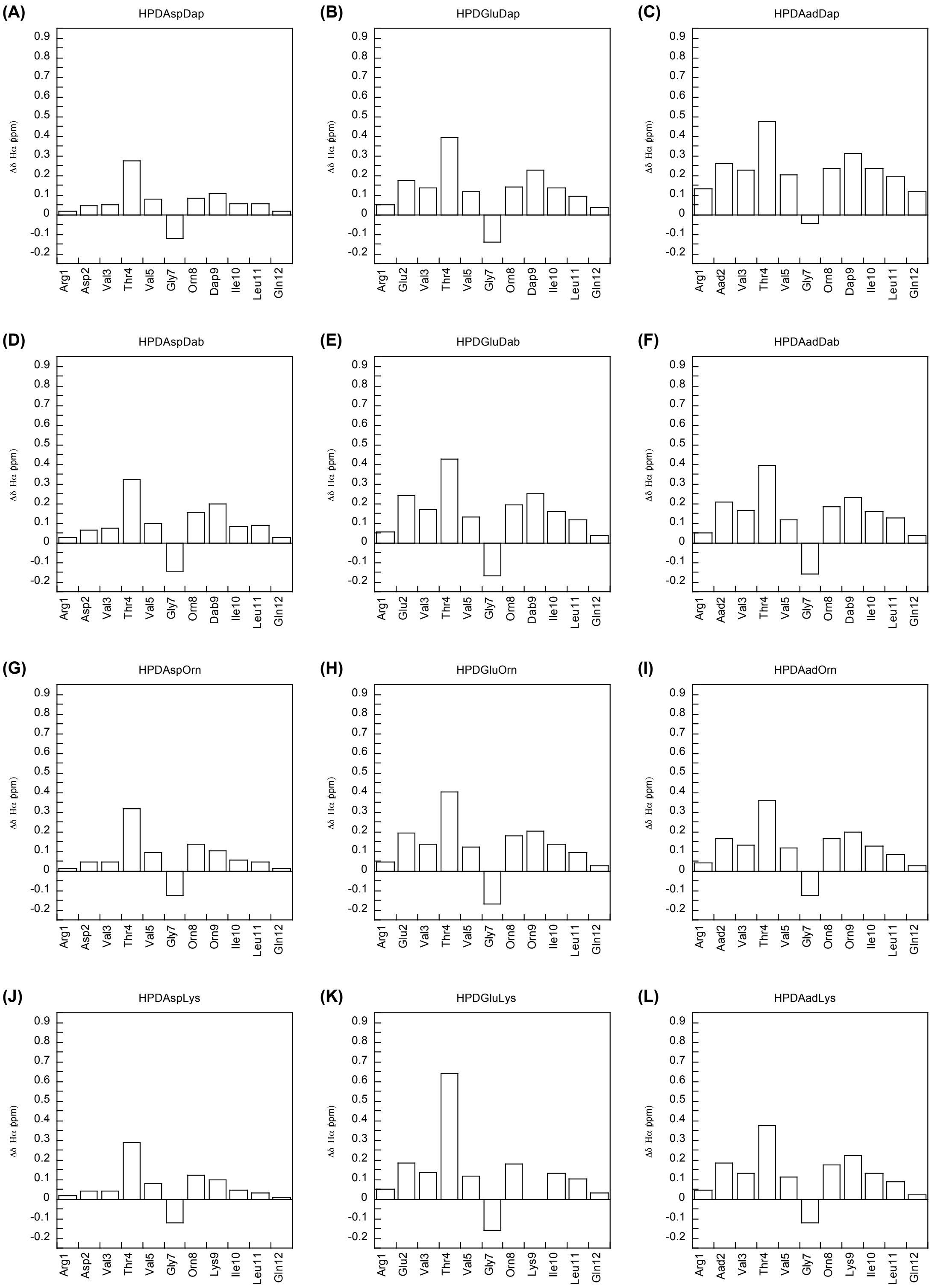

Figure S1. The Hα chemical shift deviation for the residues in the experimental HPDZbbXaa peptides: HPDAspDap (A), HPDGluDap (B), HPDAadDap (C), HPDAspDab (D), HPDGluDab (E), HPDAadDab (F), HPDAspOrn (G), HPDGluOrn (H), HPDAadOrn (I), HPDAspLys (J), HPDGluLys (K), HPDAadLys (L).

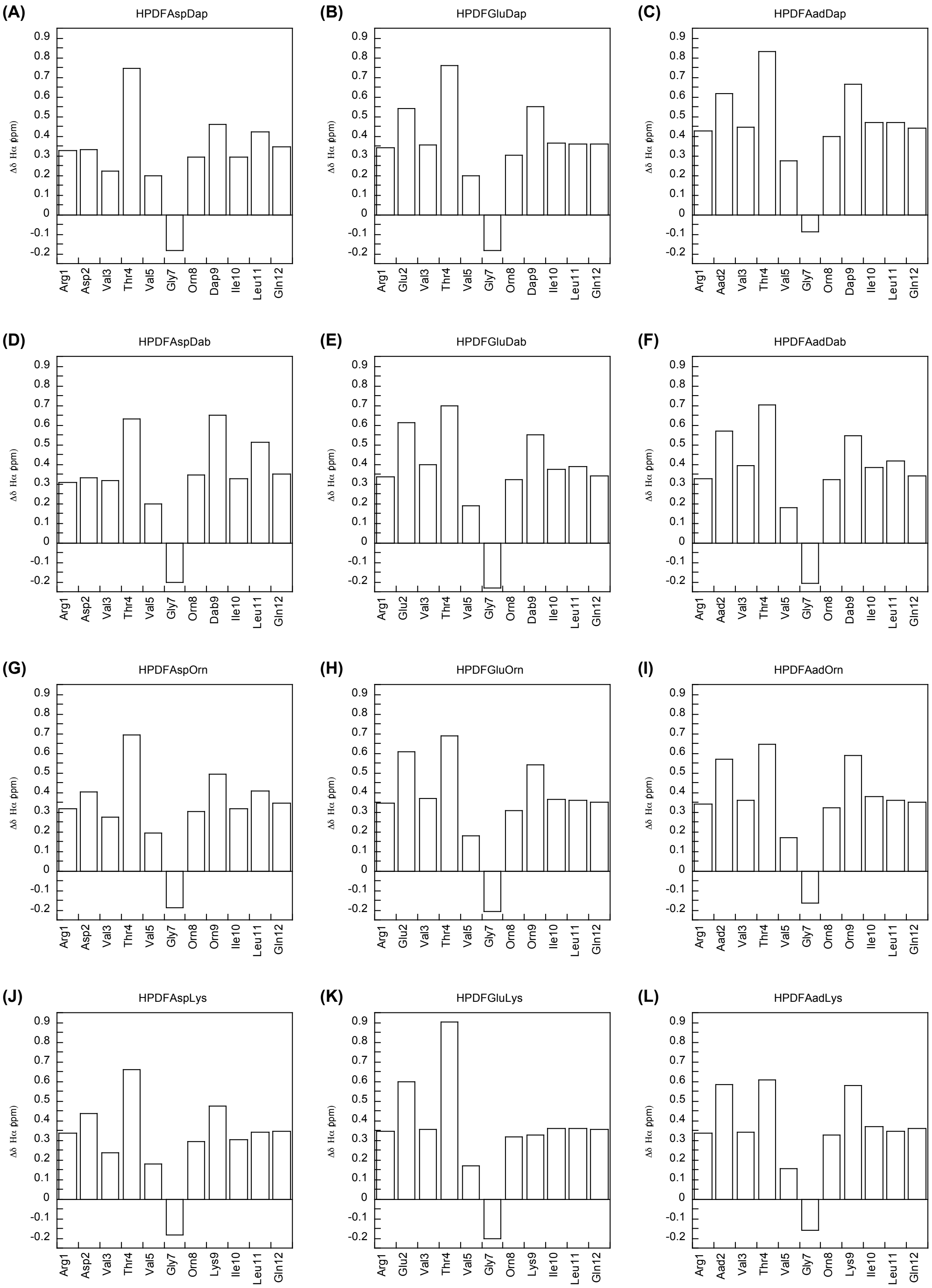

Figure S2. The Hα chemical shift deviation for the residues in the fully folded reference HPDFZbbXaa peptides: HPDFAspDap (A), HPDFGluDap (B), HPDFAadDap (C), HPDFAspDab (D), HPDFGluDab (E), HPDFAadDab (F), HPDFAspOrn (G), HPDFGluOrn (H), HPDFAadOrn (I), HPDFAspLys (J), HPDFGluLys (K), HPDFAadLys (L).

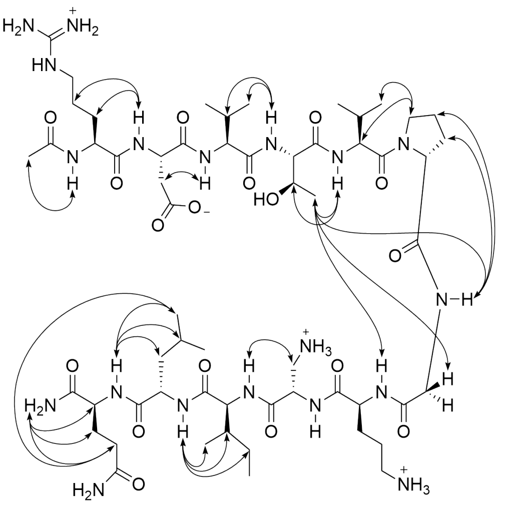

Figure S3. The NOEs in the ROESY spectra of HPDAspDap involving side chain protons.

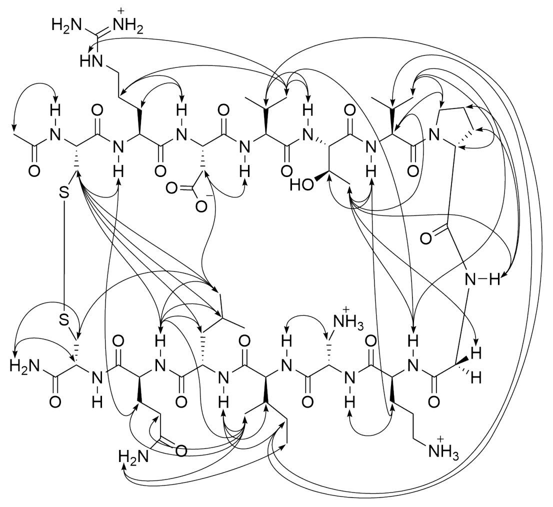

Figure S4. The NOEs in the ROESY spectra of HPDFAspDap involving side chain protons.

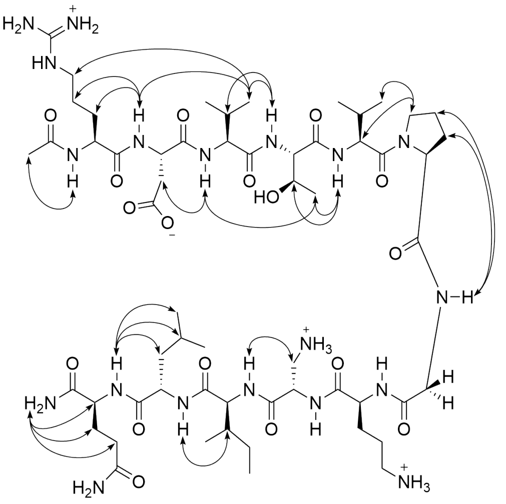

Figure S5. The NOEs in the ROESY spectra of HPDUAspDap involving side chain protons.

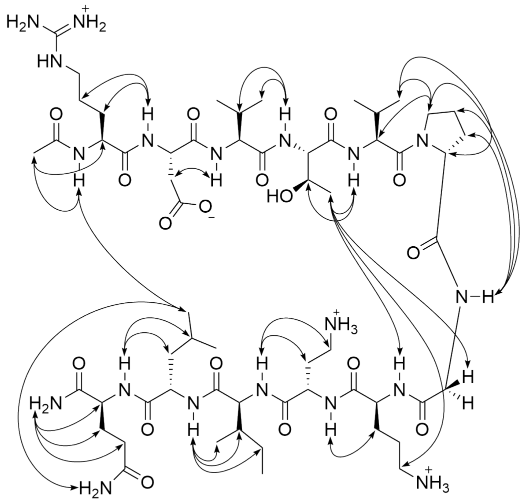

Figure S6. The NOEs in the ROESY spectra of HPDAspDab involving side chain protons.

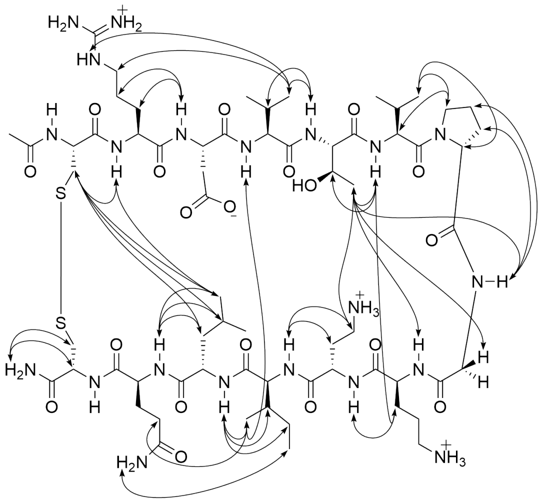

Figure S7. The NOEs in the ROESY spectra of HPDFAspDab involving side chain protons.

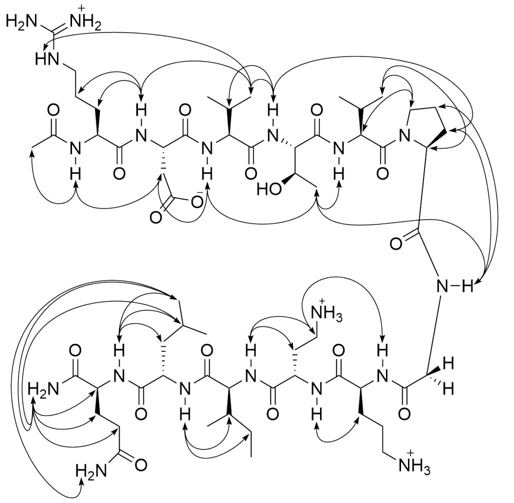

Figure S8. The NOEs in the ROESY spectra of HPDUAspDab involving side chain protons.

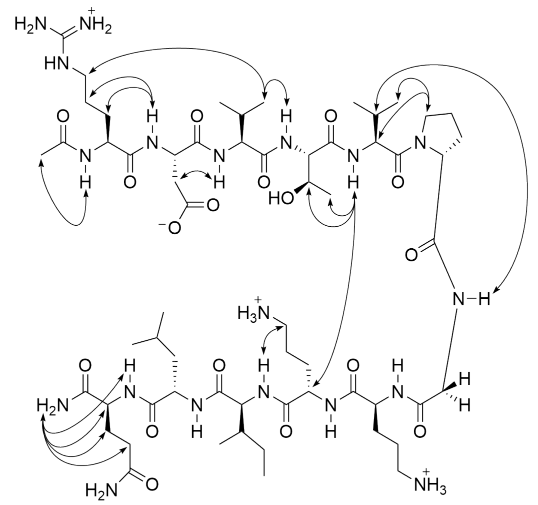

Figure S9. The NOEs in the ROESY spectra of HPDAspOrn involving side chain protons.

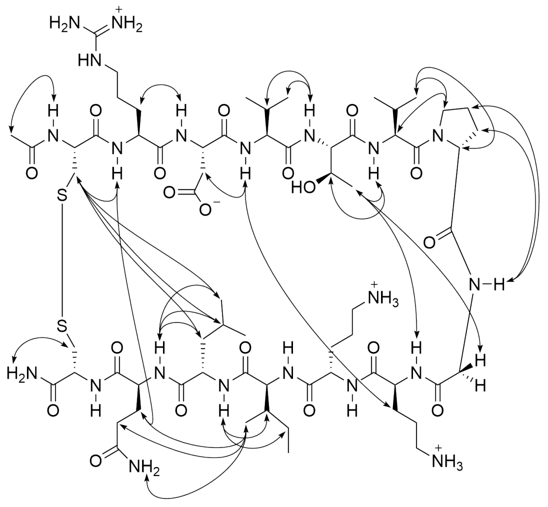

Figure S10. The NOEs in the ROESY spectra of HPDFAspOrn involving side chain protons.

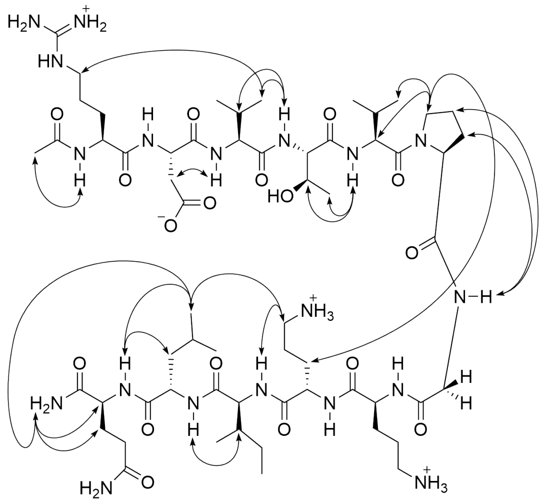

Figure S11. The NOEs in the ROESY spectra of HPDUAspOrn involving side chain protons.

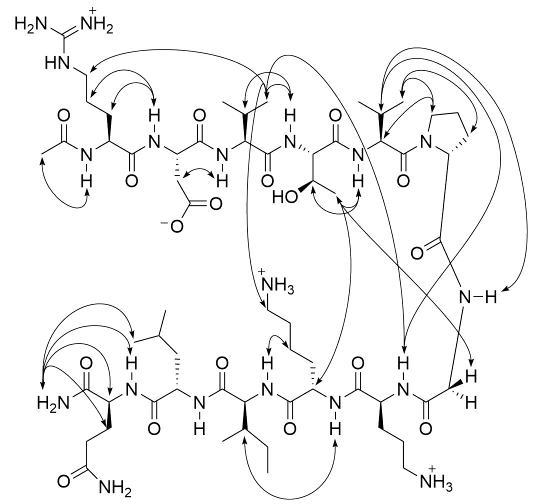

Figure S12. The NOEs in the ROESY spectra of HPDAspLys involving side chain protons.

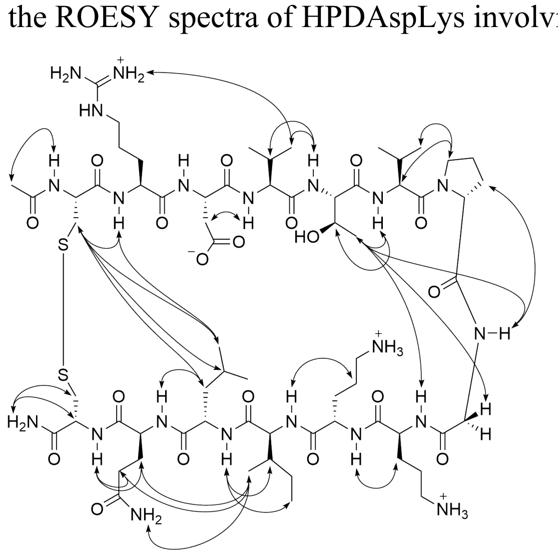

Figure S13. The NOEs in the ROESY spectra of HPDFAspLys involving side chain protons.

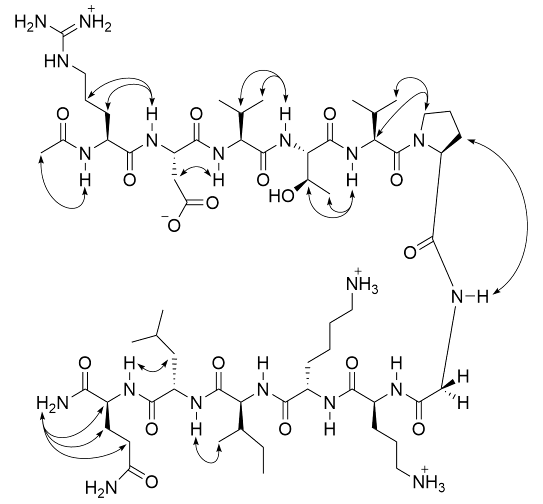

Figure S14. The NOEs in the ROESY spectra of HPDUAspLys involving side chain protons.

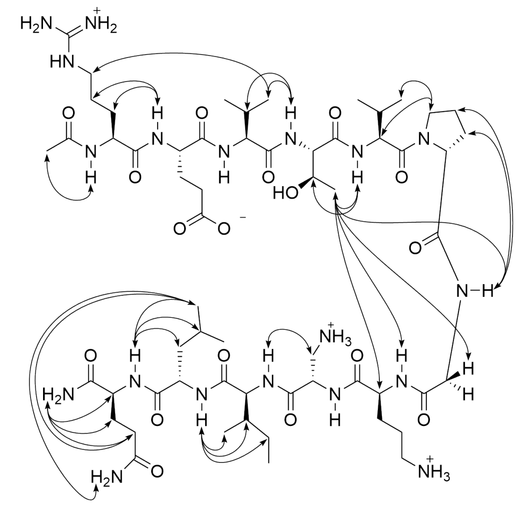

Figure S15. The NOEs in the ROESY spectra of HPDGluDap involving side chain protons.

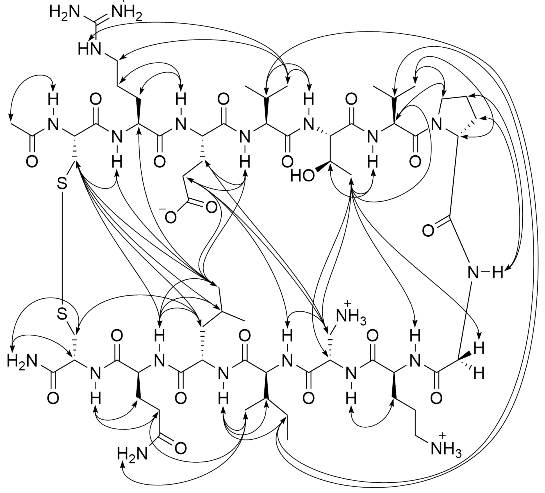

Figure S16. The NOEs in the ROESY spectra of HPDFGluDap involving side chain protons.

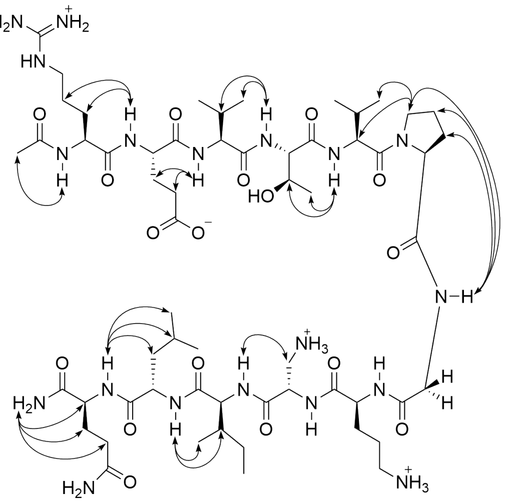

Figure S17. The NOEs in the ROESY spectra of HPDUGluDap involving side chain protons.

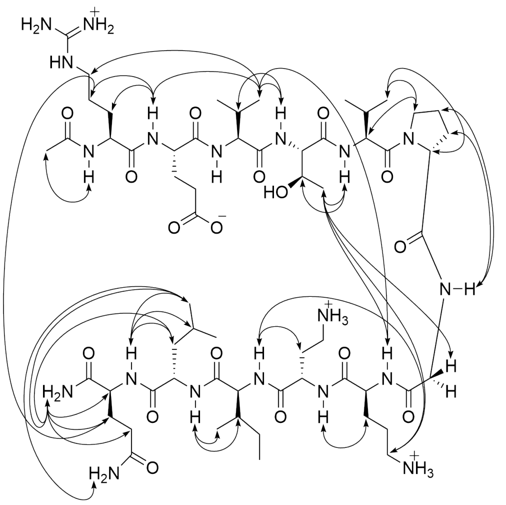

Figure S18. The NOEs in the ROESY spectra of HPDGluDab involving side chain protons.

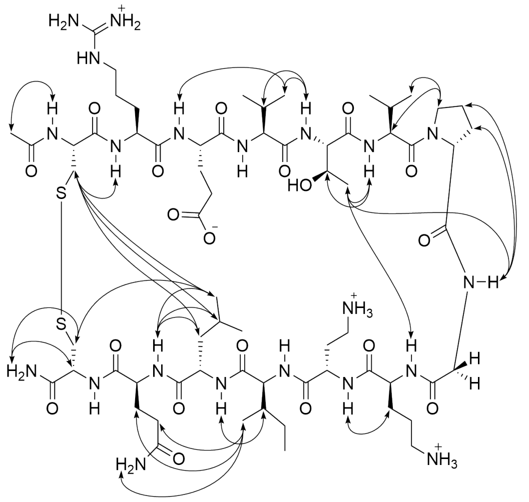

Figure S19. The NOEs in the ROESY spectra of HPDFGluDab involving side chain protons.

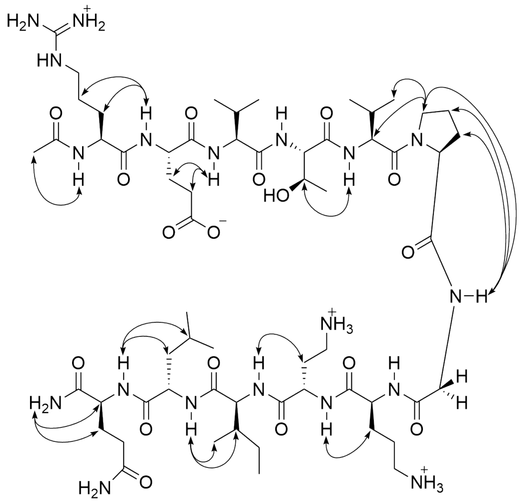

Figure S20. The NOEs in the ROESY spectra of HPDUGluDab involving side chain protons.

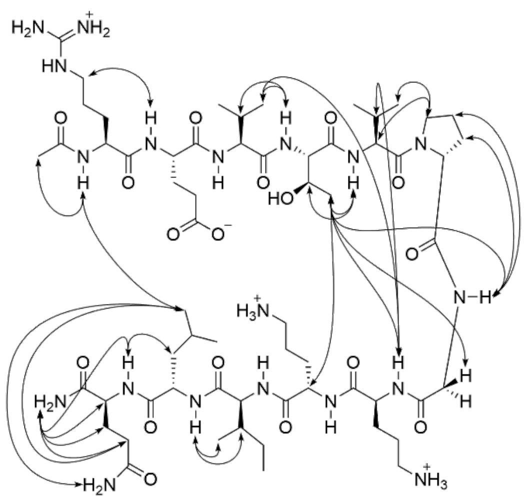

Figure S21. The NOEs in the ROESY spectra of HPDGluOrn involving side chain protons.

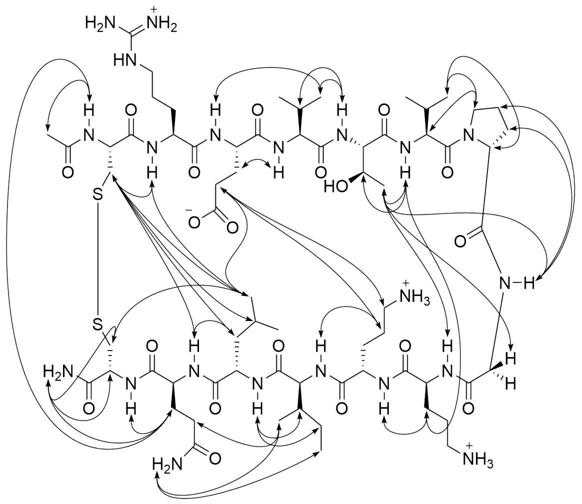

Figure S22. The NOEs in the ROESY spectra of HPDFGluOrn involving side chain protons.

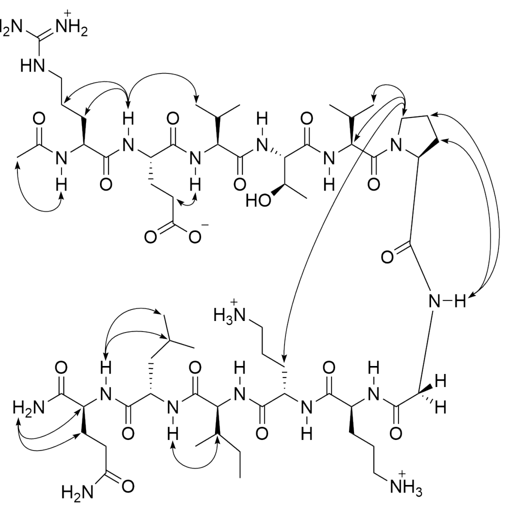

Figure S23. The NOEs in the ROESY spectra of HPDUGluOrn involving side chain protons.

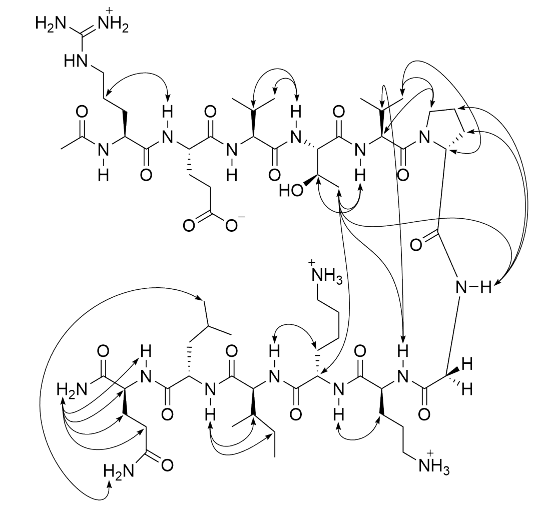

Figure S24. The NOEs in the ROESY spectra of HPDGluLys involving side chain protons.

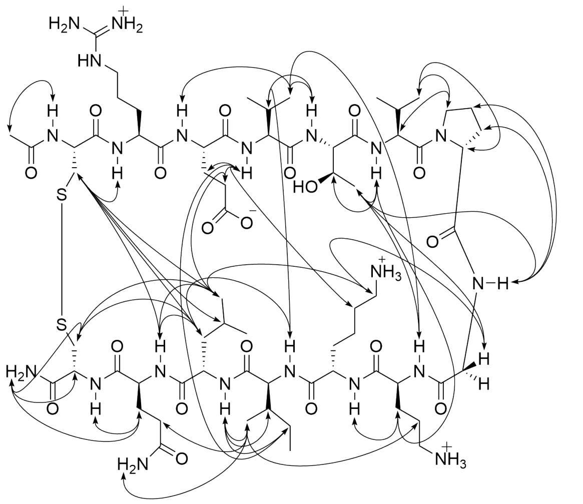

Figure S25. The NOEs in the ROESY spectra of HPDFGluLys involving side chain protons.

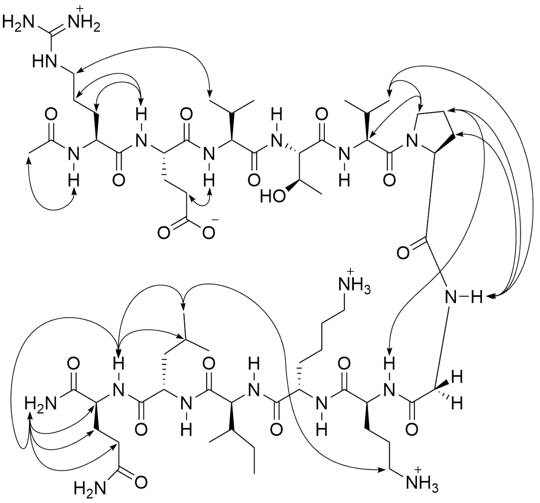

Figure S26. The NOEs in the ROESY spectra of HPDUGluLys involving side chain protons.

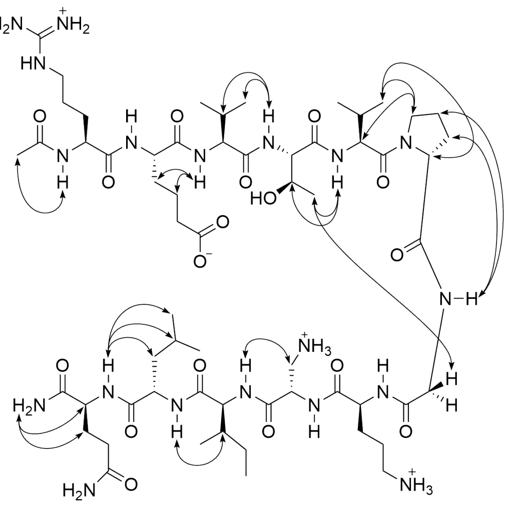

Figure S27. The NOEs in the ROESY spectra of HPDAadDap involving side chain protons.

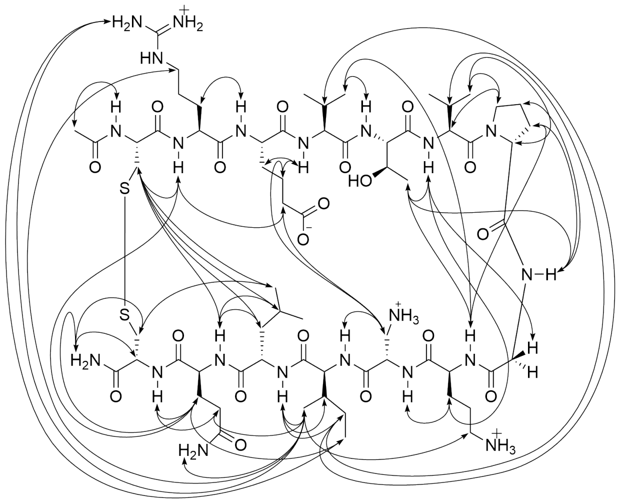

Figure S28. The NOEs in the ROESY spectra of HPDFAadDap involving side chain protons.

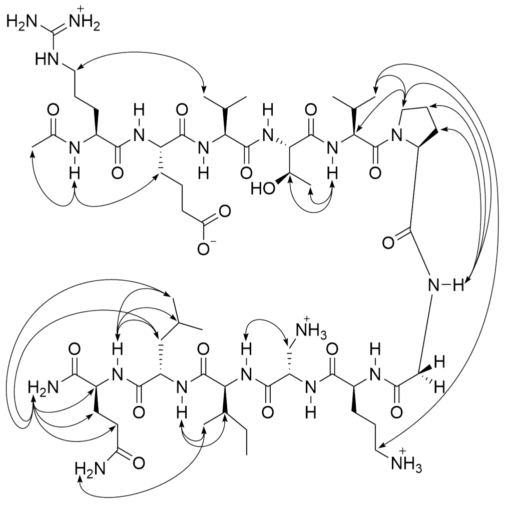

Figure S29. The NOEs in the ROESY spectra of HPDUAadDap involving side chain protons.

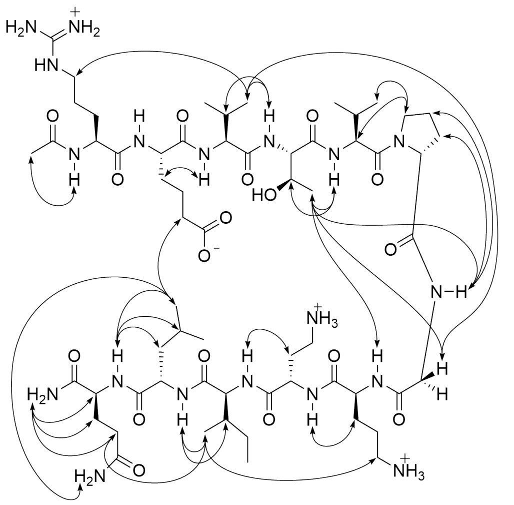

Figure S30. The NOEs in the ROESY spectra of HPDAadDab involving side chain protons.

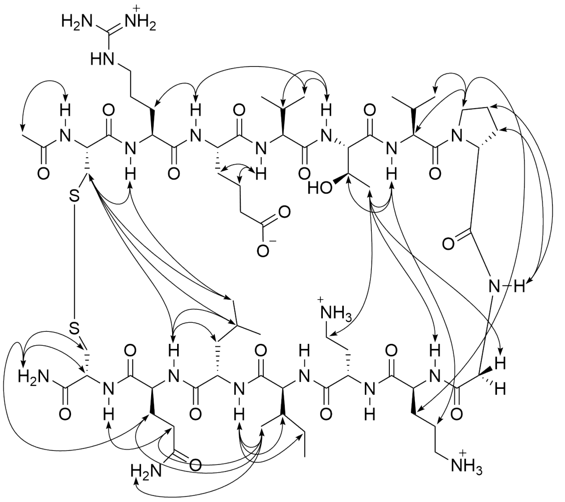

Figure S31. The NOEs in the ROESY spectra of HPDFAadDab involving side chain protons.

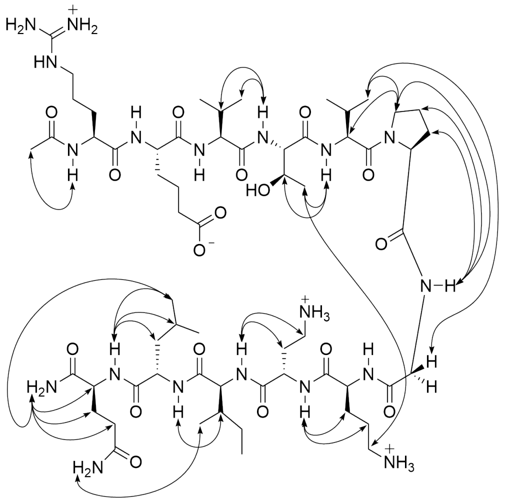

Figure S32. The NOEs in the ROESY spectra of HPDUAadDab involving side chain protons.

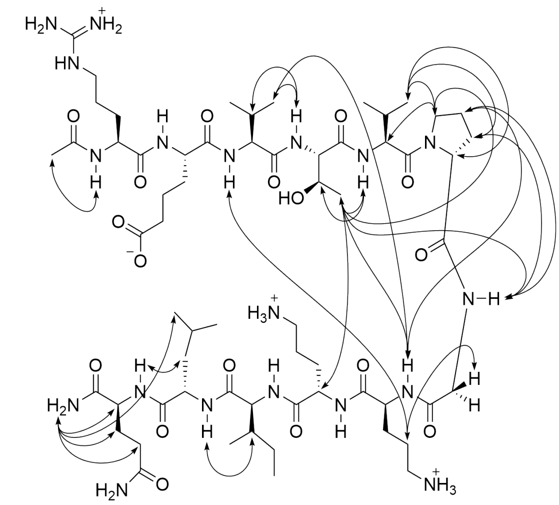

Figure S33. The NOEs in the ROESY spectra of HPDAadOrn involving side chain protons.

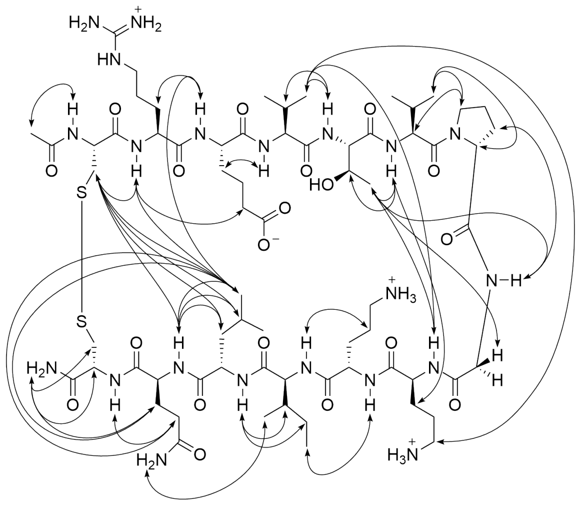

Figure S34. The NOEs in the ROESY spectra of HPDFAadOrn involving side chain protons.

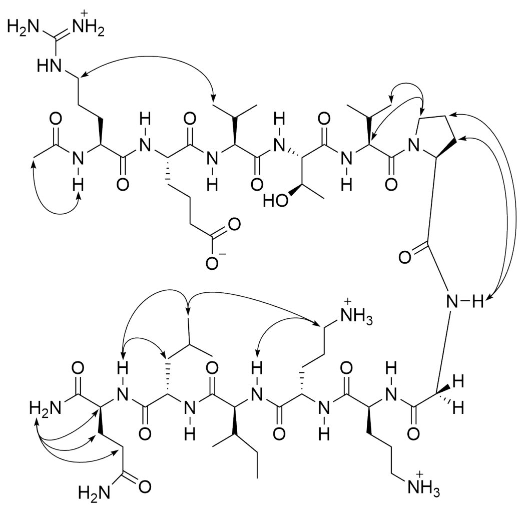

Figure S35. The NOEs in the ROESY spectra of HPDUAadOrn involving side chain protons.

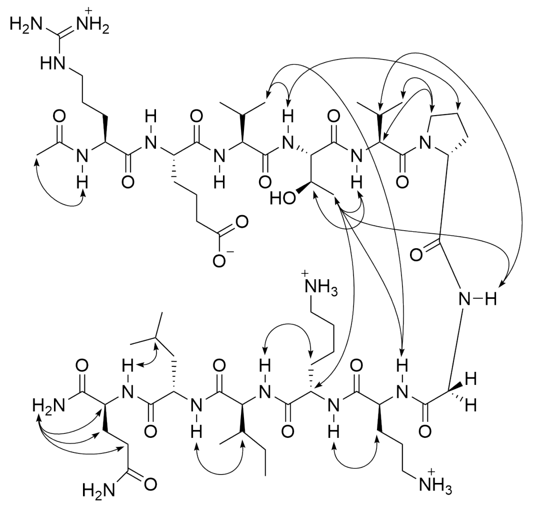

Figure S36. The NOEs in the ROESY spectra of HPDAadLys involving side chain protons.

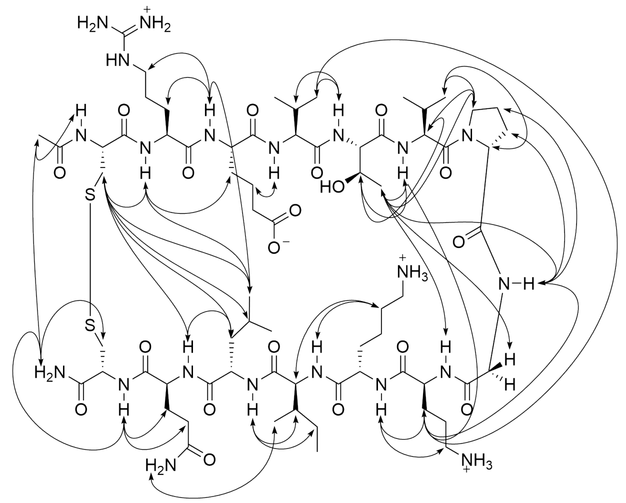

Figure S37. The NOEs in the ROESY spectra of HPDFAadLys involving side chain protons.

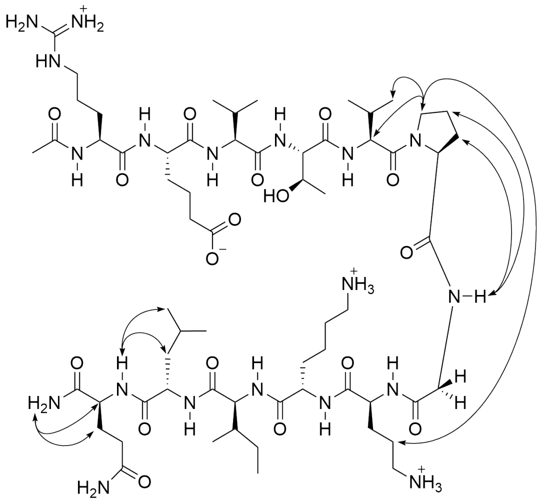

Figure S38. The NOEs in the ROESY spectra of HPDUAadLys involving side chain protons.

HPDAspDap

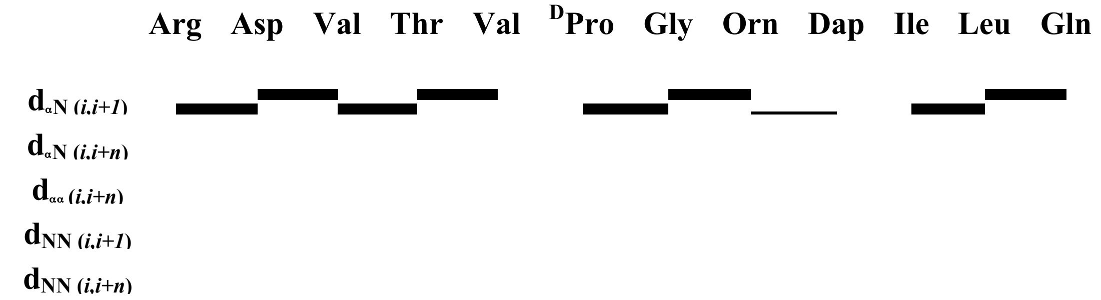

HPDFAspDap

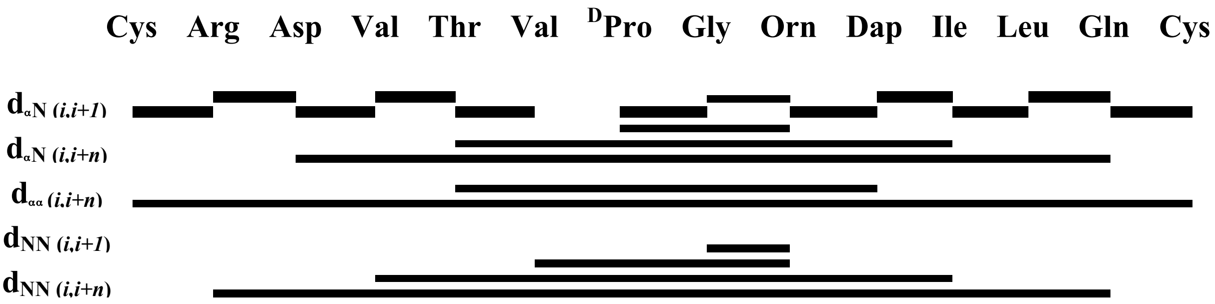

HPDUAspDap

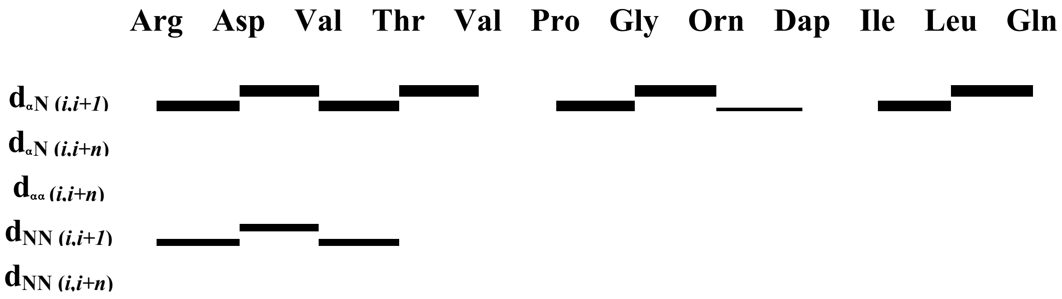

Figure S39. Wüthrich diagrams of the backbone NOE connectivities involving the α-protons and amide protons for peptides HPDAspDap, HPDFAspDap, and HPDUAspDap.

HPDAspDab

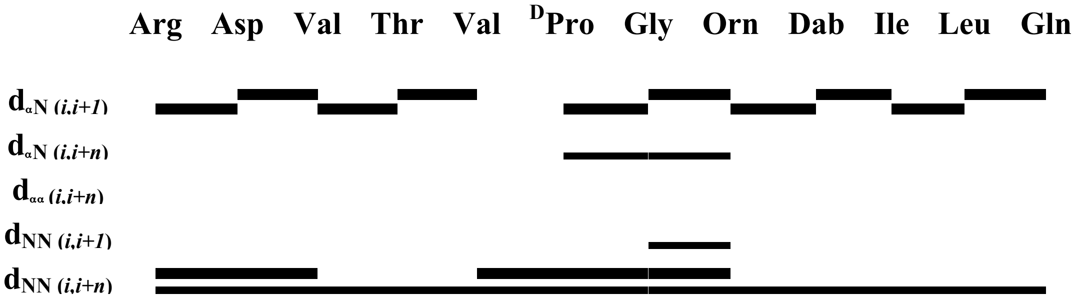

HPDFAspDab

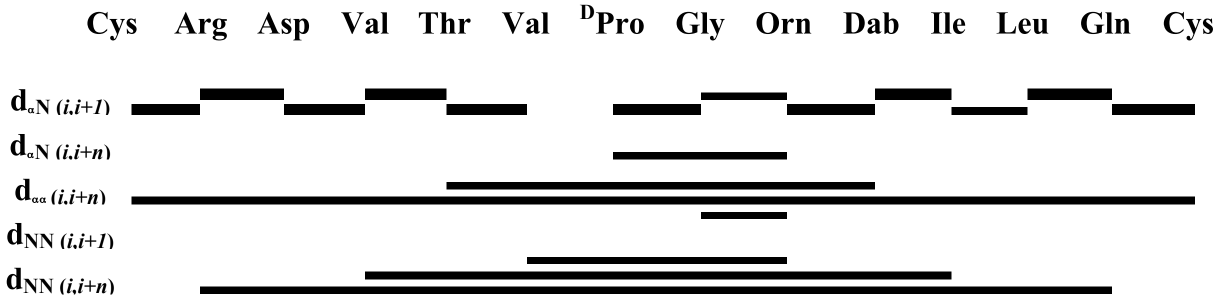

HPDUAspDab

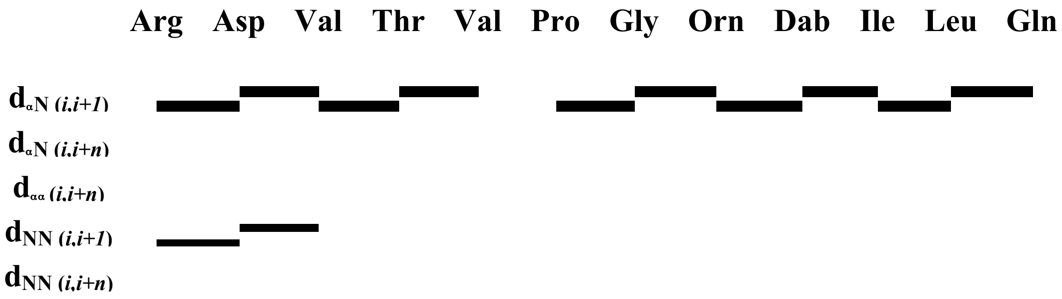

Figure S40. Wüthrich diagrams of the backbone NOE connectivities involving the α-protons and amide protons for peptides HPDAspDab, HPDFAspDab, and HPDUAspDab.

HPDAspOrn

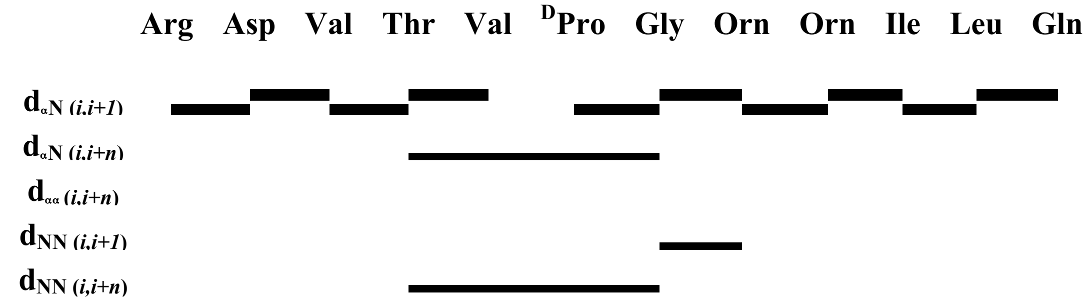

HPDFAspOrn

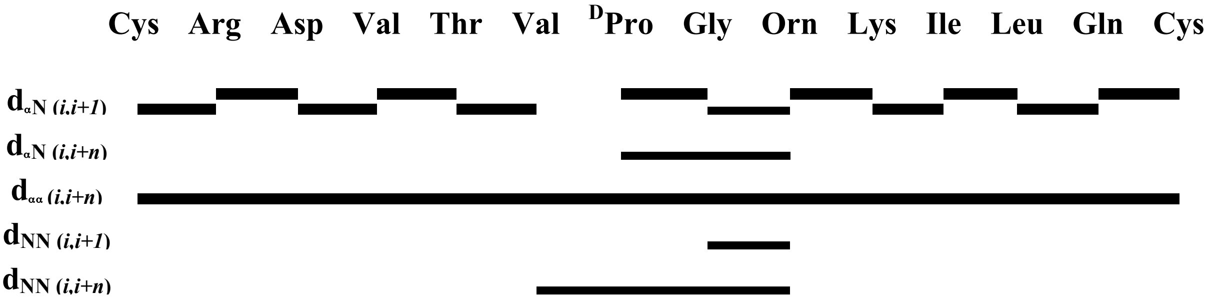

HPDUAspOrn

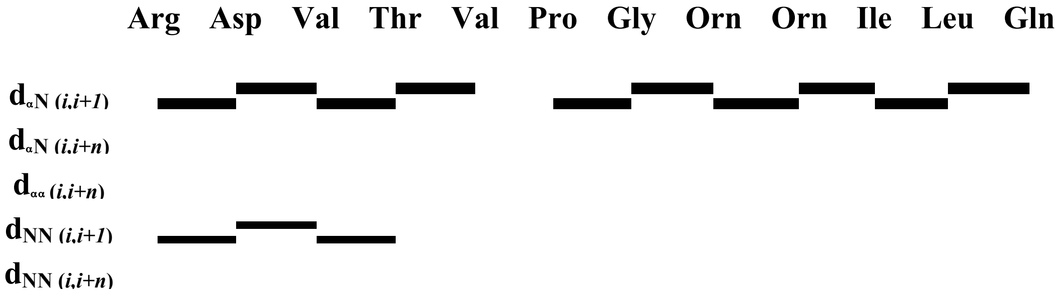

Figure S41. Wüthrich diagrams of the backbone NOE connectivities involving the α-protons and amide protons for peptides HPDAspOrn, HPDFAspOrn, and HPDUAspOrn.

HPDAspLys

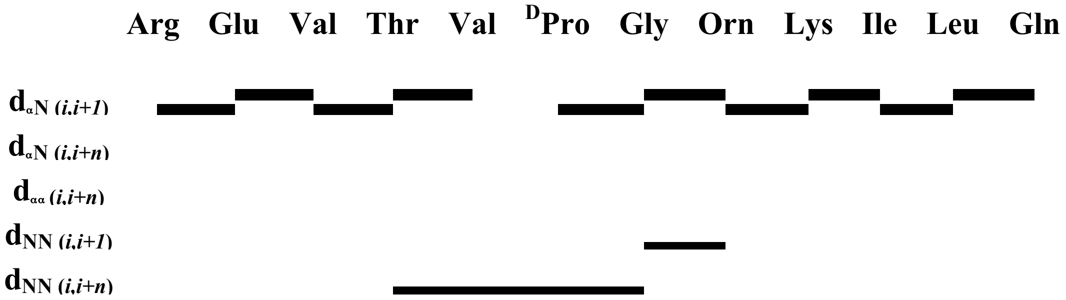

HPDFAspLys

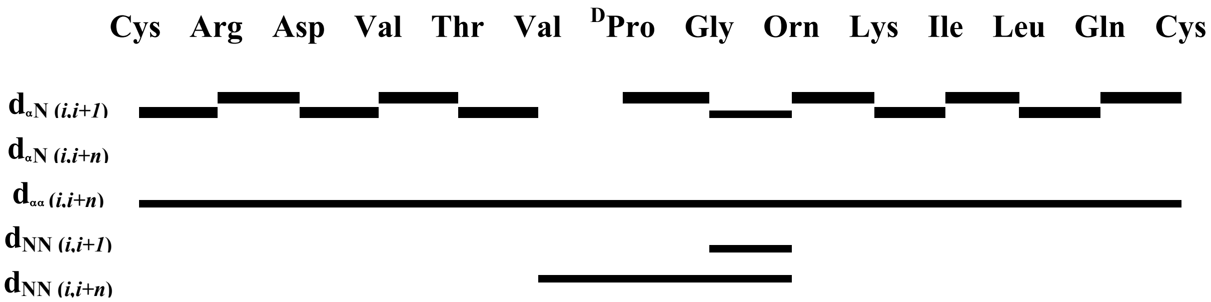

HPDUAspLys

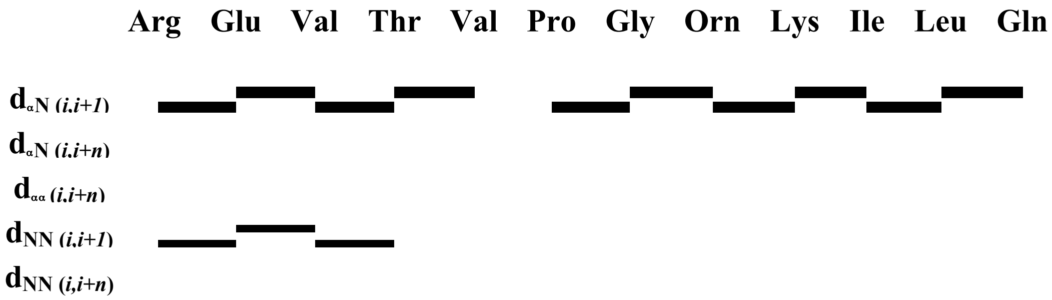

Figure S42. Wüthrich diagrams of the backbone NOE connectivities involving the α-protons and amide protons for peptides HPDAspLys, HPDFAspLys, and HPDUAspLys.

HPDGluDap

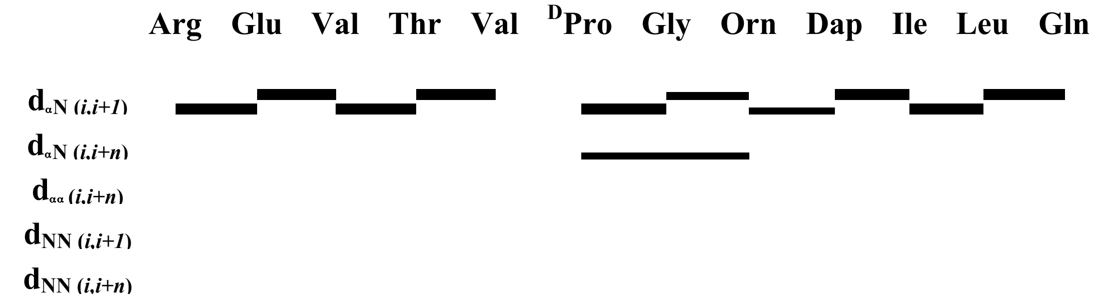

HPDFGluDap

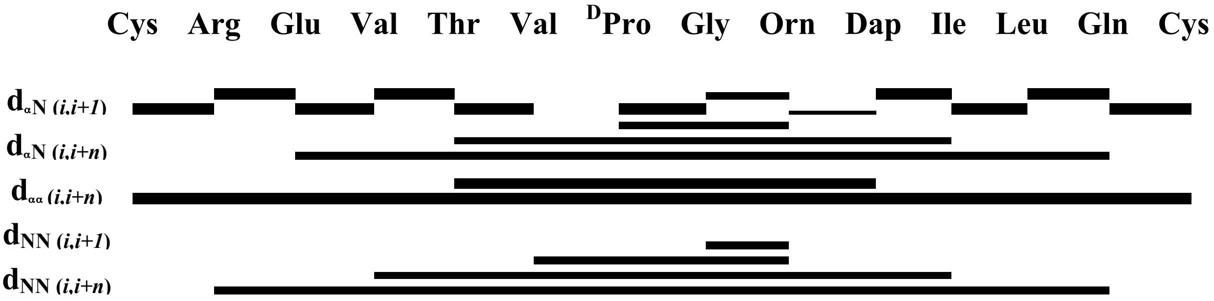

HPDUGluDap

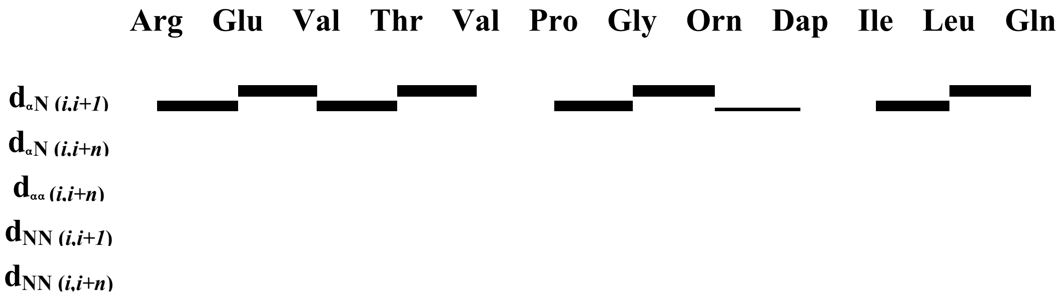

Figure S43. Wüthrich diagrams of the backbone NOE connectivities involving the α-protons and amide protons for peptides HPDGluDap, HPDFGluDap, and HPDUGluDap.

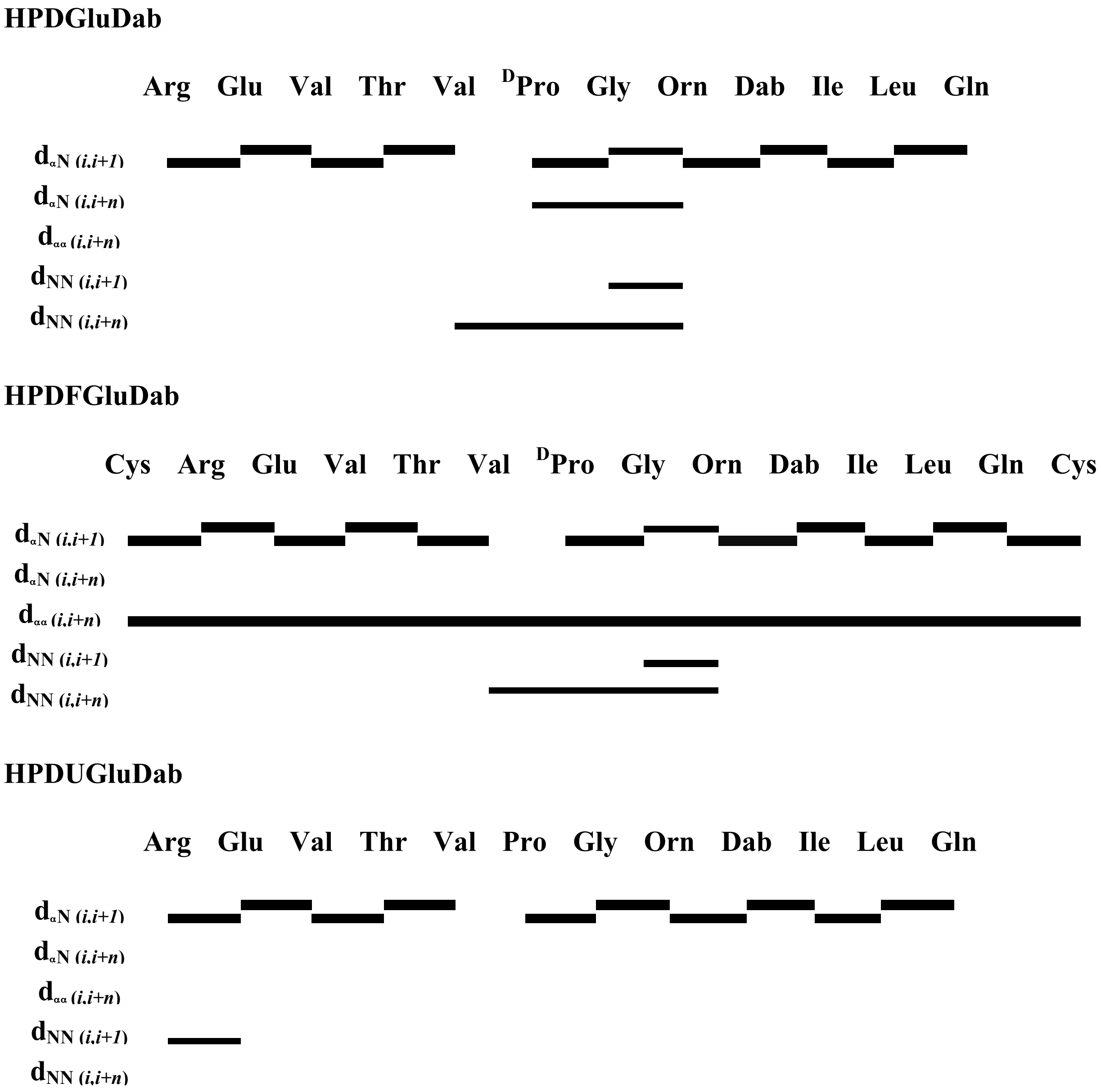

Figure S44. Wüthrich diagrams of the backbone NOE connectivities involving the α-protons and amide protons for peptides HPDGluDab, HPDFGluDab, and HPDUGluDab.

HPDGluOrn

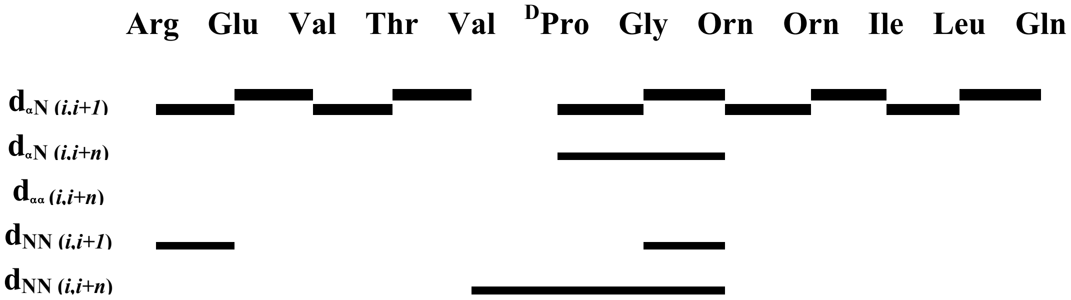

HPDFGluOrn

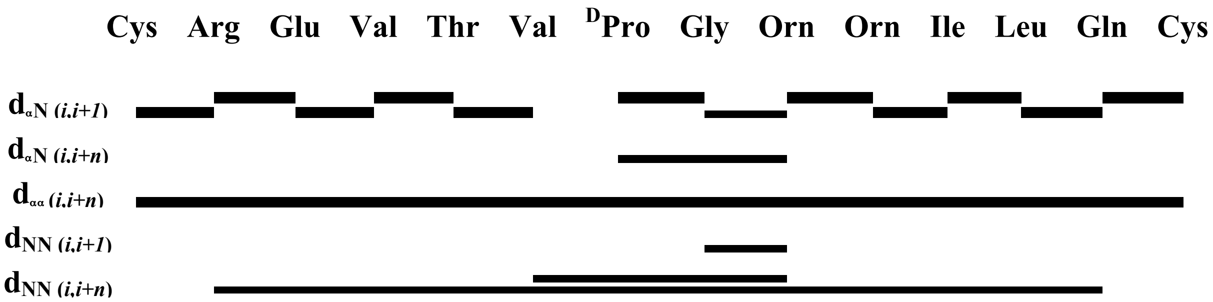

HPDUGluOrn

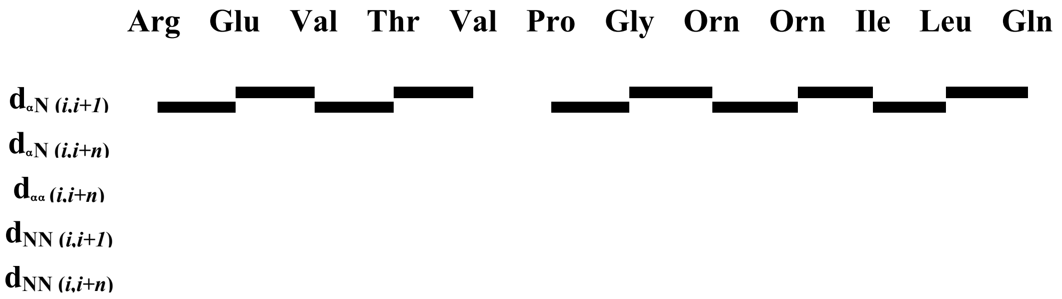

Figure S45. Wüthrich diagrams of the backbone NOE connectivities involving the α-protons and amide protons for peptides HPDGluOrn, HPDFGluOrn, and HPDUGluOrn.

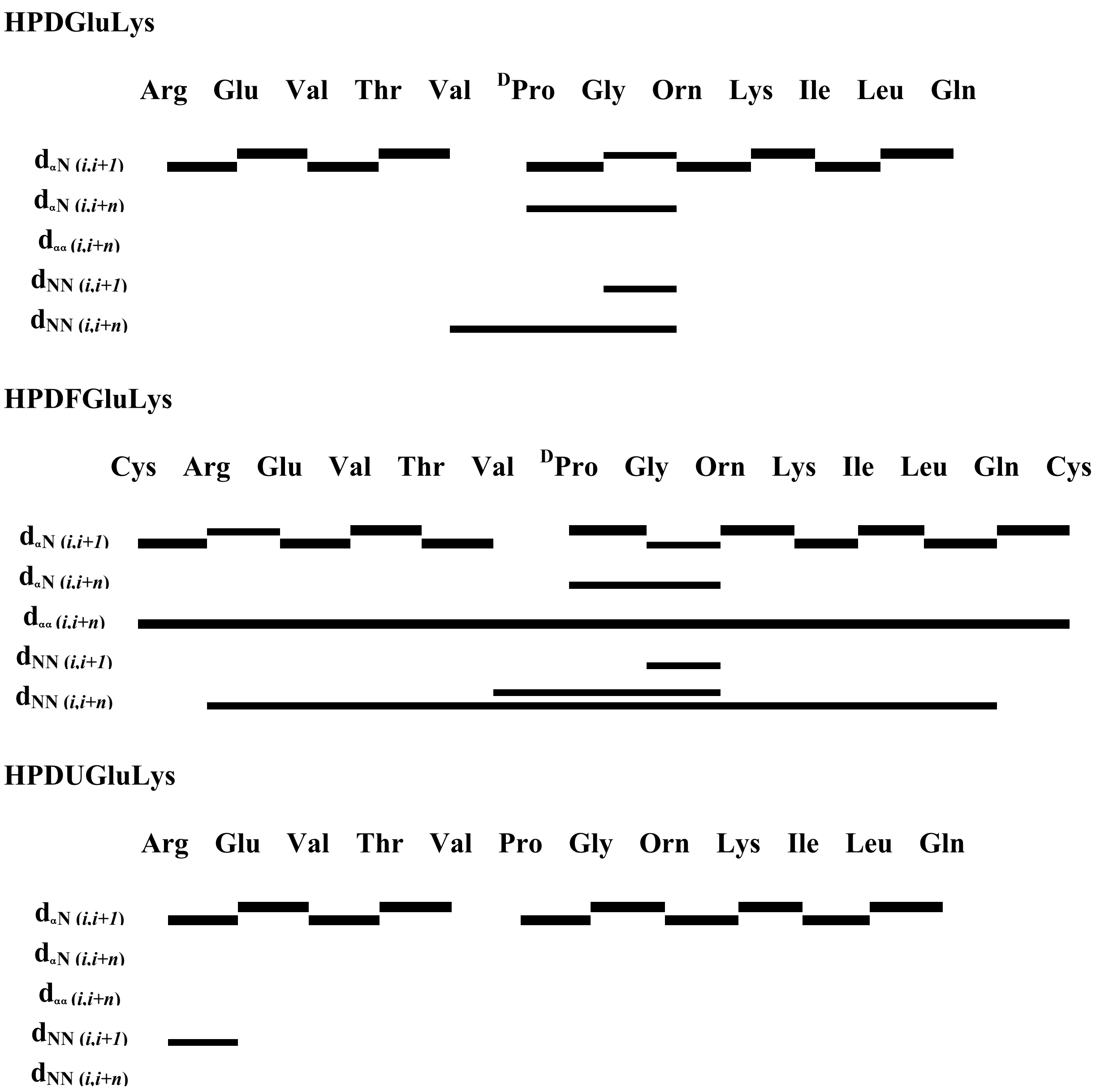

Figure S46. Wüthrich diagrams of the backbone NOE connectivities involving the α-protons and amide protons for peptides HPDGluLys, HPDFGluLys, and HPDUGluLys.

HPDAadDap

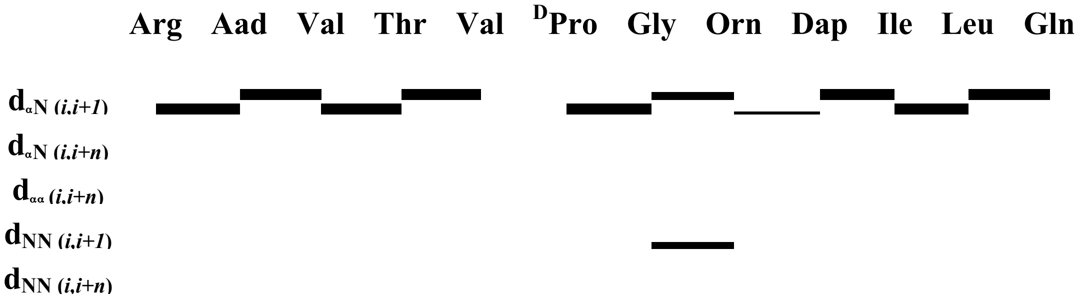

HPDFAadDap

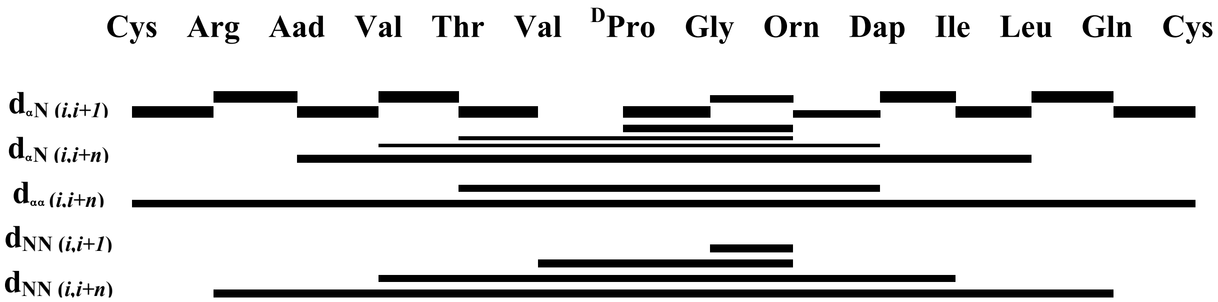

HPDUAadDap

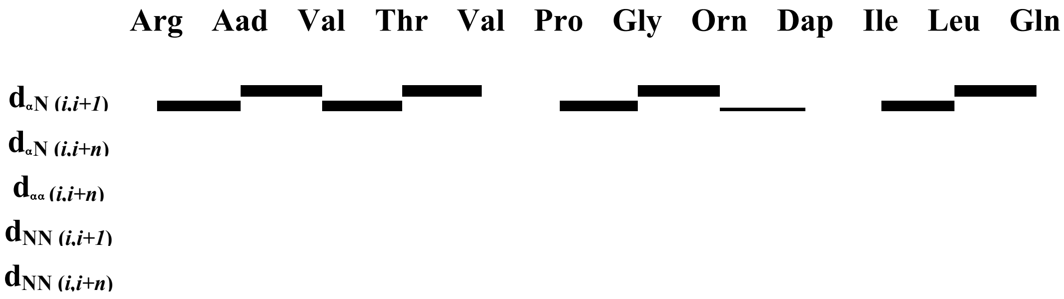

Figure S47. Wüthrich diagrams of the backbone NOE connectivities involving the α-protons and amide protons for peptides HPDAadDap, HPDFAadDap, and HPDUAadDap.

HPDAadDab

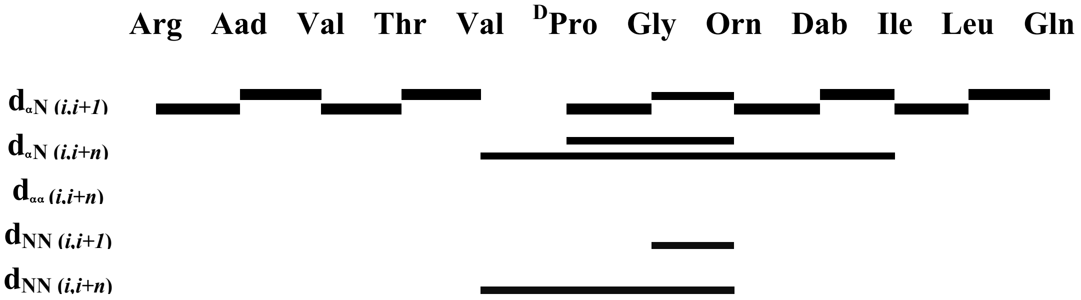

HPDFAadDab

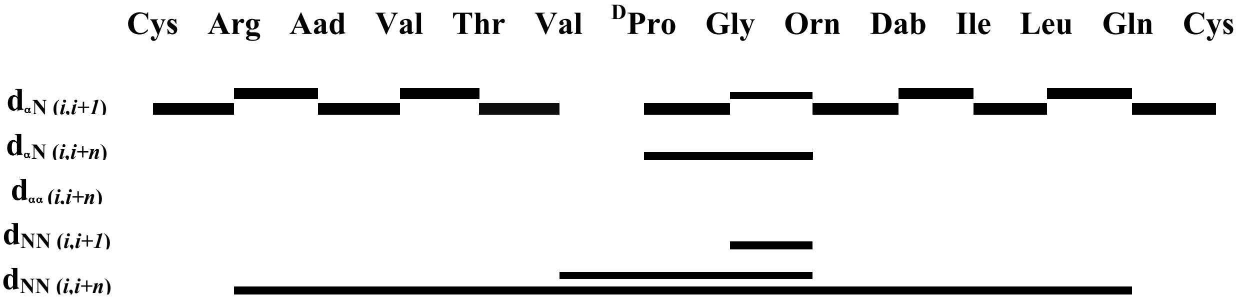

HPDUAadDab

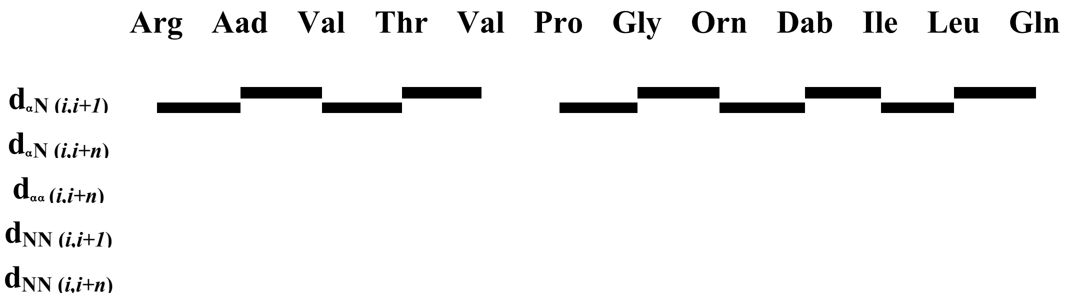

Figure S48. Wüthrich diagrams of the backbone NOE connectivities involving the α-protons and amide protons for peptides HPDAadDab, HPDFAadDab, and HPDUAadDab.

HPDAadOrn

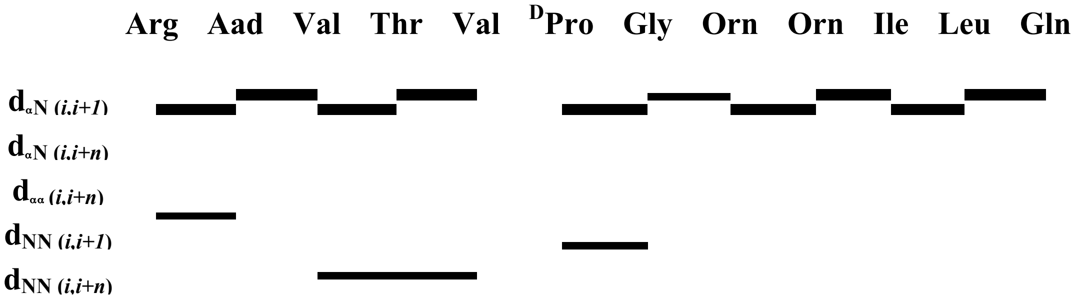

HPDFAadOrn

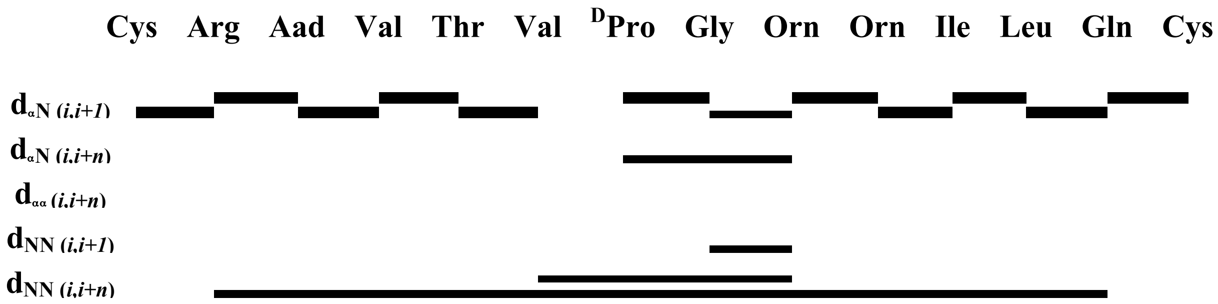

HPDUAadOrn

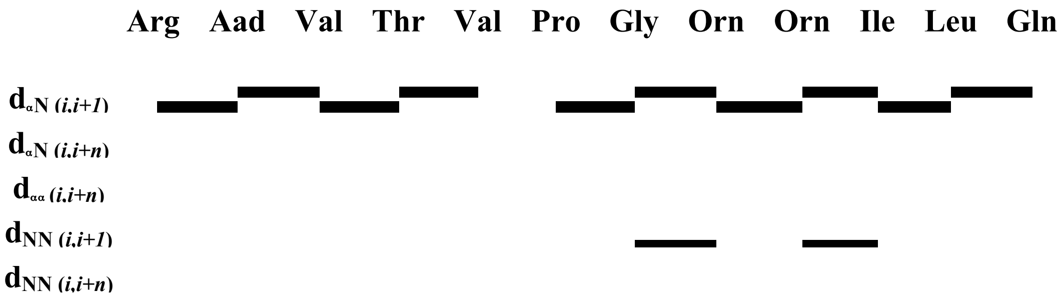

Figure S49. Wüthrich diagrams of the backbone NOE connectivities involving the α-protons and amide protons for peptides HPDAadOrn, HPDFAadOrn, and HPDUAadOrn.

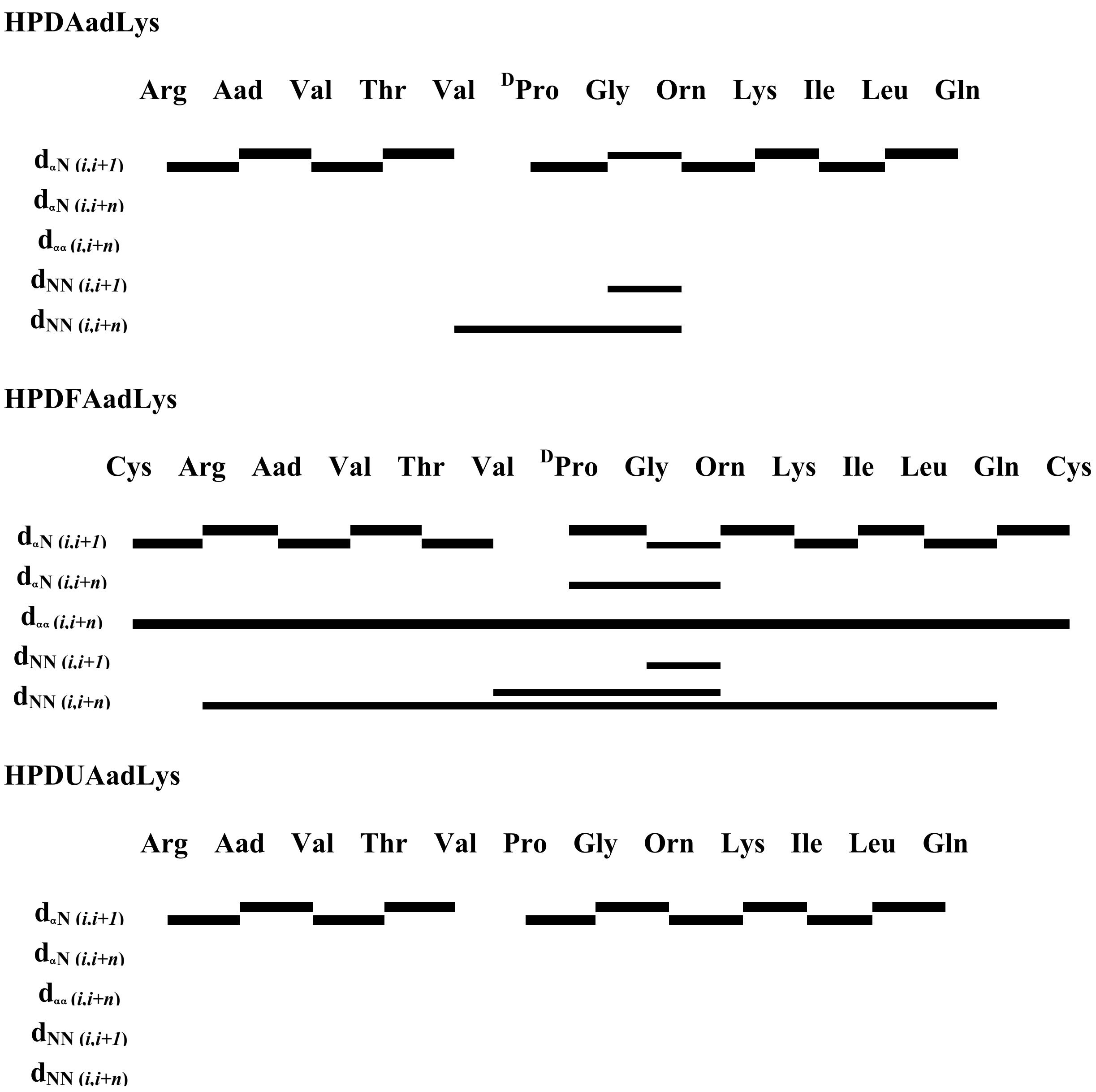

Figure S50. Wüthrich diagrams of the backbone NOE connectivities involving the α-protons and amide protons for peptides HPDAadLys, HPDFAadLys, and HPDUAadLys.

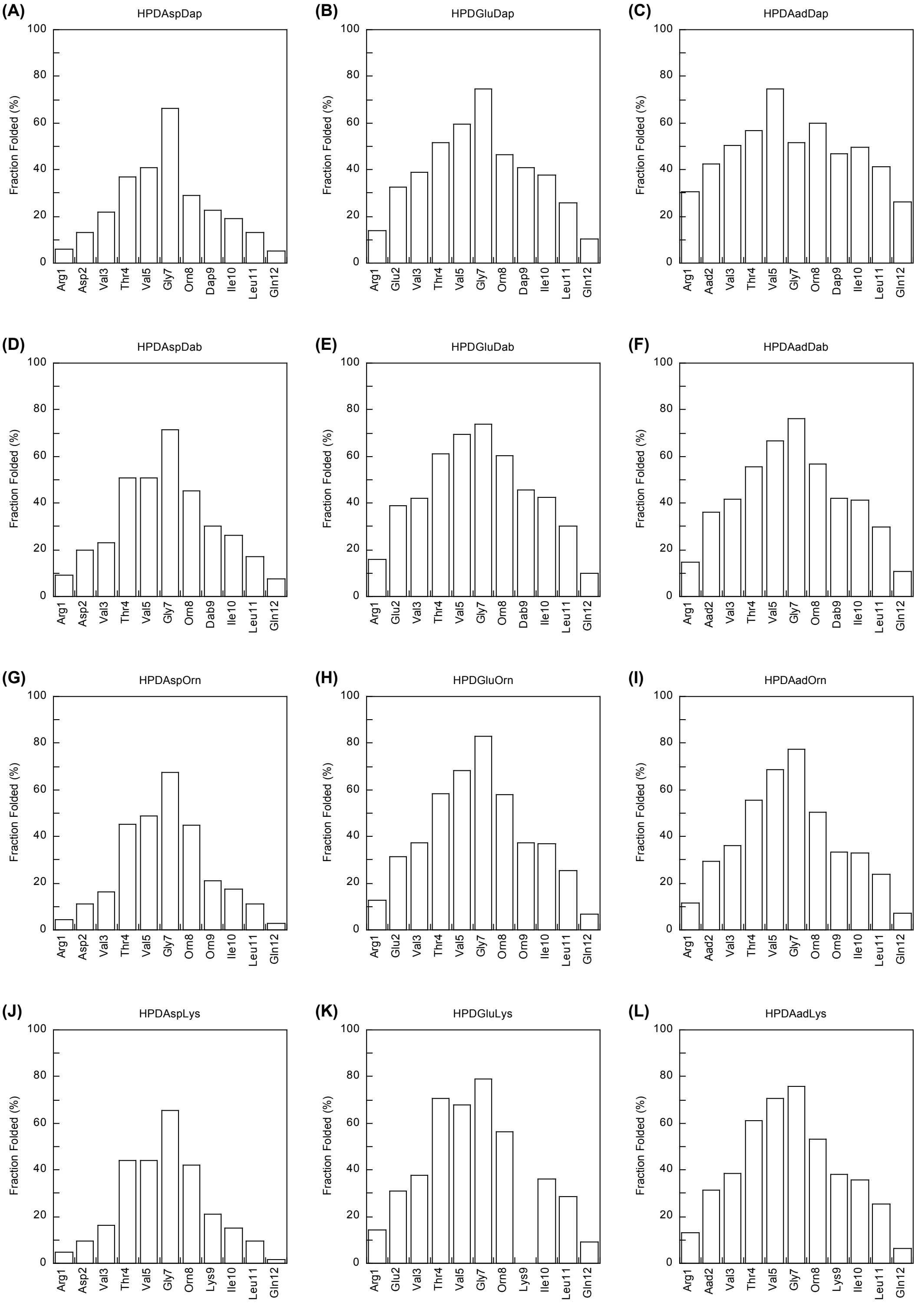

Figure S51. The fraction folded of the residues in HPDZbbXaa peptides: HPDAspDap (A), HPDGluDap (B), HPDAadDap (C), HPDAspDab (D), HPDGluDab (E), HPDAadDab (F), HPDAspOrn (G), HPDGluOrn (H), HPDAadOrn (I), HPDAspLys (J), HPDGluLys (K), HPDAadLys (L).

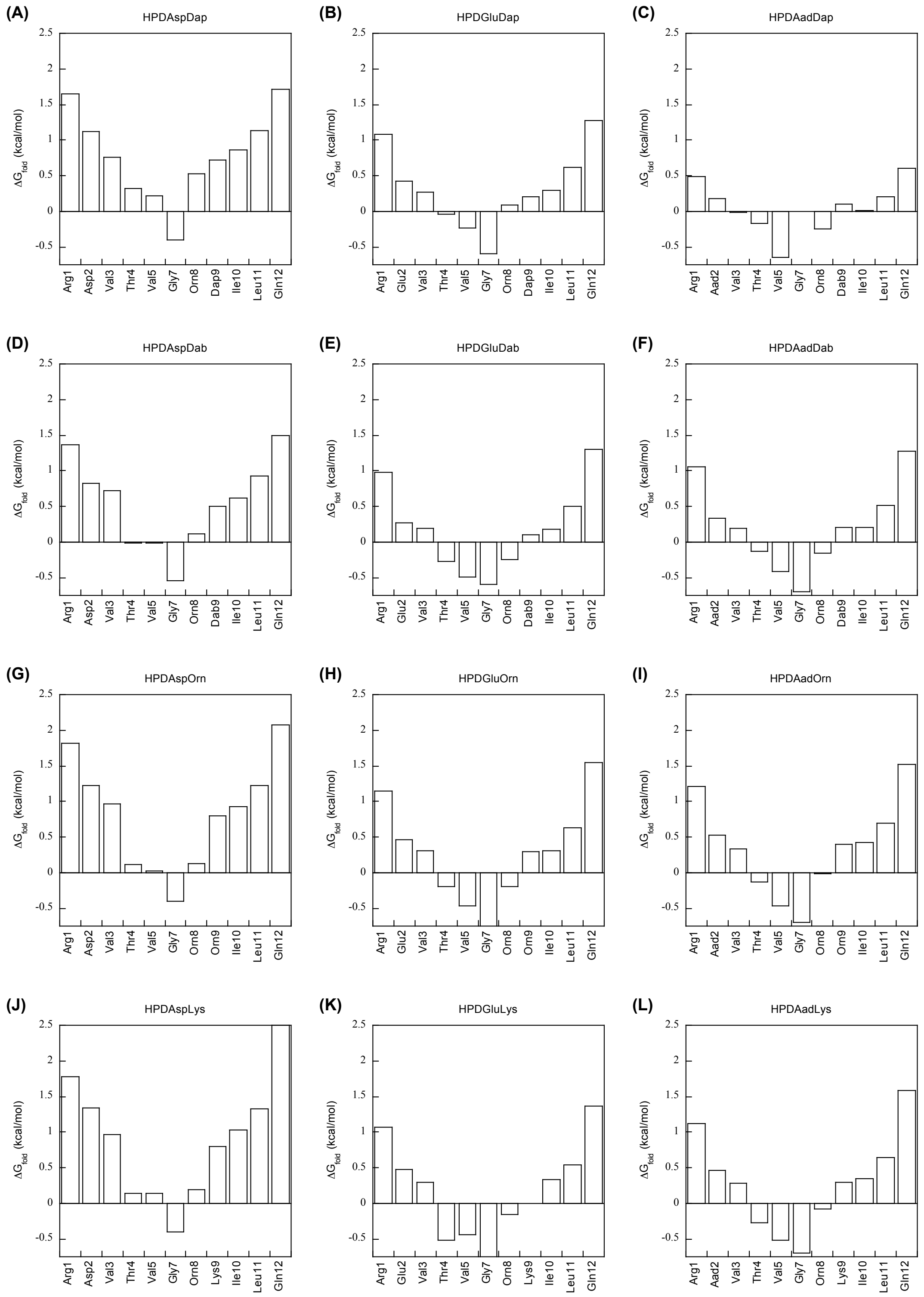

Figure S52. The ΔG_{fold} of the residues in HPDZbbXaa peptides: HPDAspDap (A), HPDGluDap (B), HPDAadDap (C), HPDAspDab (D), HPDGluDab (E), HPDAadDab (F), HPDAspOrn (G), HPDGluOrn (H), HPDAadOrn (I), HPDAspLys (J), HPDGluLys (K), HPDAadLys (L).

Material and Methods

General Section

All reagents and solvents were used without further purification. Diisopropylethylamine (DIEA), piperidine, trifluoroacetic acid (TFA), acetic anhydride (Ac_2O), and triisopropylsilane (TIS) were purchased from Acros. 1-Hydroxybenzotriazole (HOBt), 2-(1H-benzotriazole-1-yl)-1,1,3,3-tetramethyluronium hexafluorophosphate (HBTU), NovaSyn® TGR resin, and all N-9-fluorenylmethoxycarbonyl (Fmoc)-amino acids except those indicated otherwise were from Novabiochem. N_{α}-Fmoc-N_{β}-Boc-L-2,3-diaminopropionic acid (Fmoc-L-Dap(Boc)-OH), N_{α}-Fmoc-N_{γ}-Boc-L-2,4-diaminobutyric acid (Fmoc-L-Dab(Boc)-OH), N_{α}-Fmoc-D-proline, dimethylformamide (DMF), methanol and acetonitrile were from Merck. N_{α}-Fmoc-aminoadipic acid-δ-tert-butyl ester (Fmoc-L-Aad(tBuO)-OH) was from Bachem. Hexanes was from Duskan. Analytical reverse phase (RP)-HPLC was performed on an Agilent 1200 series chromatography system using a Vydac C_{18} column (4.6 mm diameter, 250 mm length). Preparative HPLC was performed on Waters Breeze chromatography system using Vydac C_4 or C_{18} column (22 mm diameter, 250 mm length). Mass spectrometry of the peptides was performed on a matrix-assisted laser desorption ionization time-of-flight (MALDI-TOF) mass spectrometer (Bruker Autoflex Speed) using α-cyano-4-hydroxycinnamic acid as the matrix. 2-Dimensional nuclear magnetic resonance spectroscopy experiments were performed on the Bruker AV III 800MHz spectrometer.

Peptide Synthesis

The peptides were synthesized by solid phase peptide synthesis (SPPS) using Fmoc-based chemistry.[1, 2] NovaSyn® TGR resin (0.050 mmol) was swollen in N,N-dimethylformamide (DMF, 3 mL) for 30 minutes. A mixture of 3 equivalents of the appropriately protected Fmoc-amino acid, HOBt, and HBTU was dissolved in DMF (1 mL). Diisopropylethylamine (DIEA, 8 equivalents) was then added to the solution and mixed thoroughly. The solution was then applied to the resin, and the residual in vial was washed into the reaction with another 1 mL DMF. The coupling times varied for different amino acids depending on their positions in the sequence. The first coupling was carried out for 8 hours. The 2nd to 7th residues were coupled for 45 minutes, and the 8th to 14th residues were coupled for 1.5 hours. The coupling time for β-branched amino acids and the sequential following amino acid were doubled. Arginine was double coupled for 30 minutes per coupling. After each coupling, the resin was washed with DMF (5 mL, 5×1 min). The Fmoc-group was then removed by 20% piperidine/DMF (3 mL, 3×8 min). After all residues were coupled, a solution of acetic anhydride (20 equivalents), DIEA (20 equivalents), and DMF (2 mL) was added to the resin for capping. The reaction was shaken for 2 hours.

The peptides were deprotected and cleaved off the resin with 95:5 trifluoroacetic acid

(TFA, 5 mL)/triisopropylsilane (TIS, 250 μL) and shaken for 2 hours. For Cys-containing peptides, 90:5:5 trifluoroacetic acid (TFA, 5 mL)/triisopropylsilane (TIS, 250 μL)/1,2-ethanedithiol (EDT, 250 μL) was used for cleavage instead. The solution was then filtered through glass wool and the resin was washed with TFA (5×1 mL). The combined filtrate was then evaporated gently by an air pump (nitrogen gas was used for the Cys-containing peptides). The resulting material was washed with hexanes (3×3 mL), dissolved in water, and lyophilized. For the Cys-containing peptides (i.e. the fully folded reference peptides), charcoal mediated air oxidation was performed to form an intramolecular disulfide bond.[3] The peptide was dissolved in 1 mM citrate, borate, and phosphate buffer solution (pH 8). Granulated charcoal (10 times of the peptide weight) was added to the solution and stirred for 2~4 hours at room temperature. After the reaction was completed, the solution was filtered through glass wool and a syringe filter (PTFE, 0.45 μm), and lyophilized. The peptide (1 mg/mL, aqueous solution) was analyzed using analytical RP-HPLC on a C_{18} column (4.6 mm diameter, 250 mm length) with 1 mL/min flow rate, temperature 25□, linear 1%/min gradient from 0% B to 100% B (solvent A: 99.9% water, 0.1% TFA; solvent B: 90% acetonitrile, 9.9% water, 0.1% TFA). Peptides were purified to greater than 95% purity (except for HPDAspDap, HPDAspDab, HPDGluDap, HPDGluDab, and HPDAadDab, to greater than 90% purity) by Sep-Pak® Plus Short tC18 cartridges using an appropriate percentage of B solvent (vide infra) and by preparative RP-HPLC using C_4 or C_{18} columns (22 mm diameter, 250 mm length) with 10 mL/min flow rate, temperature 25□, linear 0.5%/min gradient. Appropriate linear gradients were used for each peptide (vide infra); for example, PLG17_27 was used to purify HPDAspDap using a C_{18} column, representing the linear gradient from 17% B to 27% B. The identity of the peptide was confirmed by MALDI-TOF mass spectroscopy.

HPDAspDap (Ac-Arg Asp Val Thr Val ^{D}Pro Gly Orn Dap Ile Leu Gln-NH_2)

The peptide was synthesized using 207.1 mg (0.052 mmol) of NovaSyn® TGR resin. The synthesis gave 313.3 mg of resin (>99% yield). The cleavage yielded 63.8 mg of crude peptide (73.7% yield). The peptide was purified by Sep-Pak® Plus Short tC18 cartridges (35% B solvent) and preparative RP-HPLC using a C18 (PLG17_27) column to give 6.8 mg of pure peptide (93.4% purity). Retention time on analytical RP-HPLC was 28.0 minutes. The identity of the peptide was confirmed by MALDI-TOF mass spectrometry. Calculated for $C_{58}H_{103}N_{19}O_{17}$ [MH]+: 1338.785; observed: 1338.808. The concentration of peptide for NMR analysis was 10.1 mM.

HPDAspDab (Ac-Arg Asp Val Thr Val ^{D}Pro Gly Orn Dab Ile Leu Gln-NH_2)

The peptide was synthesized using 206.2 mg (0.052 mmol) of NovaSyn® TGR resin. The synthesis gave 293.5 mg of resin (88.5% yield). The cleavage yielded 70.1 mg of crude

peptide (98.6% yield). The peptide was purified by Sep-Pak® Plus Short tC18 cartridges (35% B solvent) and preparative RP-HPLC using a C18 (PLG17_27) column to give 8.0 mg of pure peptide (91.2% purity). Retention time on analytical RP-HPLC was 28.4 minutes. The identity of the peptide was confirmed by MALDI-TOF mass spectrometry. Calculated for $C_{59}H_{105}N_{19}O_{17}$ [MH]+: 1352.801; observed: 1352.848. The concentration of peptide for NMR analysis was 8.9 mM.

HPDAspOrn (Ac-Arg Asp Val Thr Val ^{D}Pro Gly Orn Orn Ile Leu Gln-NH_2)

The peptide was synthesized using 208.5 mg (0.052 mmol) of NovaSyn® TGR resin. The synthesis gave 321.4 mg of resin (>99% yield). The cleavage yielded 60.4 mg of crude peptide (62.0% yield). The peptide was purified by preparative RP-HPLC using C4 (PLG08_18) and C18 (PLG17_27) columns to give 2.7 mg of pure peptide (96.8% purity). Retention time on analytical RP-HPLC was 27.7 minutes. The identity of the peptide was confirmed by MALDI-TOF mass spectrometry. Calculated for $C_{60}H_{107}N_{19}O_{17}$ [MH]+: 1366.817; observed: 1366.956. The concentration of peptide for NMR analysis was 4.0 mM.

HPDAspLys (Ac-Arg Asp Val Thr Val ^{D}Pro Gly Orn Lys Ile Leu Gln-NH_2)

The peptide was synthesized using 208.2 mg (0.052 mmol) of NovaSyn® TGR resin. The synthesis gave 292.9 mg of resin (87.0% yield). The cleavage yielded 54.0 mg of crude peptide (73.0% yield). The peptide was purified by preparative RP-HPLC using C4 (PLG07_17) and C18 (PLG17_27) columns to give 4.8 mg of pure peptide (95.6% purity). Retention time on analytical RP-HPLC was 28.2 minutes. The identity of the peptide was confirmed by MALDI-TOF mass spectrometry. Calculated for $C_{61}H_{109}N_{19}O_{17}$ [MH]+: 1380.832; observed: 1380.930. The concentration of peptide for NMR analysis was 7.0 mM.

HPDGluDap (Ac-Arg Glu Val Thr Val ^{D}Pro Gly Orn Dap Ile Leu Gln-NH_2)

The peptide was synthesized using 200.6 mg (0.050 mmol) of NovaSyn® TGR resin. The synthesis gave 289.5 mg of resin (93.2% yield). The cleavage yielded 63.8 mg of crude peptide (78.8% yield). The peptide was purified by Sep-Pak® Plus Short tC18 cartridges (35% B solvent) and preparative RP-HPLC using a C18 (PLG17_27) column to give 3.1 mg of pure peptide (94.8% purity). Retention time on analytical RP-HPLC was 26.9 minutes. The identity of the peptide was confirmed by MALDI-TOF mass spectrometry. Calculated for $C_{59}H_{105}N_{19}O_{17}$ [MH]+: 1352.801; observed: 1352.888. The concentration of peptide for NMR analysis was 4.6 mM.

HPDGluDab (Ac-Arg Glu Val Thr Val ^{D}Pro Gly Orn Dab Ile Leu Gln-NH_2)

The peptide was synthesized using 209.2 mg (0.052 mmol) of NovaSyn® TGR resin. The synthesis gave 281.6 mg of resin (71.8% yield). The cleavage yielded 68.8 mg of crude

peptide (>99% yield). The peptide was purified by Sep-Pak® Plus Short tC18 cartridges (35% B solvent) and preparative RP-HPLC using a C18 (PLG16_26) column to give 4.2 mg of pure peptide (93.5% purity). Retention time on analytical RP-HPLC was 27.5 minutes. The identity of the peptide was confirmed by MALDI-TOF mass spectrometry. Calculated for $C_{60}H_{107}N_{19}O_{17}$ [MH]+: 1366.817; observed: 1366.878. The concentration of peptide for NMR analysis was 6.1 mM.

HPDGluOrn (Ac-Arg Glu Val Thr Val ^{D}Pro Gly Orn Orn Ile Leu Gln-NH_2)

The peptide was synthesized using 204.8 mg (0.051 mmol) of NovaSyn® TGR resin. The synthesis gave 334.6 mg of resin (>99% yield). The cleavage yielded 82.2 mg of crude peptide (74.0% yield). The peptide was purified by preparative RP-HPLC using C4 (PLG07_17) and C18 (PLG16_26) columns to give 10.3 mg of pure peptide (95.7% purity). Retention time on analytical RP-HPLC was 27.7 minutes. The identity of the peptide was confirmed by MALDI-TOF mass spectrometry. Calculated for $C_{61}H_{109}N_{19}O_{17}$ [MH]+: 1380.832; observed: 1380.705. The concentration of peptide for NMR analysis was 7.5 mM.

HPDGluLys (Ac-Arg Glu Val Thr Val ^{D}Pro Gly Orn Lys Ile Leu Gln-NH_2)

The peptide was synthesized using 202.4 mg (0.051 mmol) of NovaSyn® TGR resin. The synthesis gave 312.7 mg of resin (>99% yield). The cleavage yielded 58.7 mg of crude peptide (61.0% yield). The peptide was purified by Sep-Pak® Plus Short tC18 cartridges (30% B solvent) and preparative RP-HPLC using a C18 (PLG16_26) column to give 4.3 mg of pure peptide (97.5% purity). Retention time on analytical RP-HPLC was 27.2 minutes. The identity of the peptide was confirmed by MALDI-TOF mass spectrometry. Calculated for $C_{62}H_{111}N_{19}O_{17}$ [MH]+: 1394.848; observed: 1394.859. The concentration of peptide for NMR analysis was 6.2 mM.

HPDAadDap (Ac-Arg Aad Val Thr Val ^{D}Pro Gly Orn Dap Ile Leu Gln-NH_2)

The peptide was synthesized using 205.2 mg (0.051 mmol) of NovaSyn® TGR resin. The synthesis gave 294.7 mg of resin (90.5% yield). The cleavage yielded 65.9 mg of crude peptide (86.6% yield). The peptide was purified by Sep-Pak® Plus Short tC18 cartridges (35% B solvent) and preparative RP-HPLC using a C18 (PLG17_27) column to give 5.9 mg of pure peptide (95.4% purity). Retention time on analytical RP-HPLC was 27.4 minutes. The identity of the peptide was confirmed by MALDI-TOF mass spectrometry. Calculated for $C_{60}H_{107}N_{19}O_{17}$ [MH]+: 1366.817; observed: 1366.822. The concentration of peptide for NMR analysis was 8.6 mM.

HPDAadDab (Ac-Arg Aad Val Thr Val ^{D}Pro Gly Orn Dab Ile Leu Gln-NH_2)

The peptide was synthesized using 203.6 mg (0.051 mmol) of NovaSyn® TGR resin. The

synthesis gave 284.9 mg of resin (82.8% yield). The cleavage yielded 46.1 mg of crude peptide (64.8% yield). The peptide was purified by Sep-Pak® Plus Short tC18 cartridges (35% B solvent) and preparative RP-HPLC using a C18 (PLG17_27) column to give 7.6 mg of pure peptide (93.1% purity). Retention time on analytical RP-HPLC was 28.0 minutes. The identity of the peptide was confirmed by MALDI-TOF mass spectrometry. Calculated for $C_{61}H_{109}N_{19}O_{17}$ [MH]+: 1380.832; observed: 1381.001. The concentration of peptide for NMR analysis was 7.0 mM.

HPDAadOrn (Ac-Arg Aad Val Thr Val ^{D}Pro Gly Orn Orn Ile Leu Gln-NH_2)

The peptide was synthesized using 202.9 mg (0.051 mmol) of NovaSyn® TGR resin. The synthesis gave 311.0 mg of resin (>99% yield). The cleavage yielded 91.4 mg of crude peptide (97.0% yield). The peptide was purified by preparative RP-HPLC using C4 (PLG06_16) and C18 (PLG16_26) columns to give 6.6 mg of pure peptide (96.3% purity). Retention time on analytical RP-HPLC was 27.6 minutes. The identity of the peptide was confirmed by MALDI-TOF mass spectrometry. Calculated for $C_{62}H_{111}N_{19}O_{17}$ [MH]+: 1394.848; observed: 1394.951. The concentration of peptide for NMR analysis was 4.8 mM.

HPDAadLys (Ac-Arg Aad Val Thr Val ^{D}Pro Gly Orn Lys Ile Leu Gln-NH_2)

The peptide was synthesized using 208.2 mg (0.052 mmol) of NovaSyn® TGR resin. The synthesis gave 309.4 mg of resin (>99% yield). The cleavage yielded 66.2 mg of crude peptide (75.0% yield). The peptide was purified by preparative RP-HPLC using C4 (PLG08_18) and C18 (PLG16_26) columns to give 7.0 mg of pure peptide (96.7% purity). Retention time on analytical RP-HPLC was 27.5 minutes. The identity of the peptide was confirmed by MALDI-TOF mass spectrometry. Calculated for $C_{63}H_{113}N_{19}O_{17}$ [MH]+: 1408.863; observed: 1408.968. The concentration of peptide for NMR analysis was 5.0 mM.

HPDUAspDap (Ac-Arg Asp Val Thr Val ^{L}Pro Gly Orn Dap Ile Leu Gln-NH_2)

The peptide was synthesized using 205.3 mg (0.051 mmol) of NovaSyn® TGR resin. The synthesis gave 280.0 mg of resin (76.8% yield). The cleavage yielded 42.7 mg of crude peptide (69.9% yield). The peptide was purified by Sep-Pak® Plus Short tC18 cartridges (35% B solvent) and preparative RP-HPLC using a C18 (PLG15_25) column to give 6.4 mg of pure peptide (96.7% purity). Retention time on analytical RP-HPLC was 24.9 minutes. The identity of the peptide was confirmed by MALDI-TOF mass spectrometry. Calculated for $C_{58}H_{103}N_{19}O_{17}$ [MH]+: 1338.785; observed: 1338.935. The concentration of peptide for NMR analysis was 9.6 mM.

HPDUAspDab (Ac-Arg Asp Val Thr Val ^{L}Pro Gly Orn Dab Ile Leu Gln-NH_2)

The peptide was synthesized using 205.2 mg (0.051 mmol) of NovaSyn® TGR resin. The

synthesis gave 288.0 mg of resin (84.3% yield). The cleavage yielded 49.9 mg of crude peptide (74.4% yield). The peptide was purified by preparative RP-HPLC using C4 (PLG04_14) and C18 (PLG14_24) columns to give 9.4 mg of pure peptide (96.1% purity). Retention time on analytical RP-HPLC was 25.0 minutes. The identity of the peptide was confirmed by MALDI-TOF mass spectrometry. Calculated for $C_{59}H_{105}N_{19}O_{17}$ [MH]+: 1352.801; observed: 1352.808. The concentration of peptide for NMR analysis was 7.0 mM.

HPDUAspOrn (Ac-Arg Asp Val Thr Val ^{L}Pro Gly Orn Orn Ile Leu Gln-NH_2)

The peptide was synthesized using 208.5 mg (0.052 mmol) of NovaSyn® TGR resin. The synthesis gave 320.8 mg of resin (>99% yield). The cleavage yielded 58.7 mg of crude peptide (62.0% yield). The peptide was purified by Sep-Pak® Plus Short tC18 cartridges (35% B solvent) and preparative RP-HPLC using a C18 (PLG15_25) column to give 7.6 mg of pure peptide (97.5% purity). Retention time on analytical RP-HPLC was 25.4 minutes. The identity of the peptide was confirmed by MALDI-TOF mass spectrometry. Calculated for $C_{60}H_{107}N_{19}O_{17}$ [MH]+: 1366.817; observed: 1366.825. The concentration of peptide for NMR analysis was 5.6 mM.

HPDUAspLys (Ac-Arg Asp Val Thr Val ^{L}Pro Gly Orn Lys Ile Leu Gln-NH_2)

The peptide was synthesized using 208.4 mg (0.052 mmol) of NovaSyn® TGR resin. The synthesis gave 323.1 mg of resin (>99% yield). The cleavage yielded 61.5 mg of crude peptide (64.0% yield). The peptide was purified by preparative RP-HPLC using C4 (PLG05_15) and C18 (PLG15_25) columns to give 8.4 mg of pure peptide (97.2% purity). Retention time on analytical RP-HPLC was 25.7 minutes. The identity of the peptide was confirmed by MALDI-TOF mass spectrometry. Calculated for $C_{61}H_{109}N_{19}O_{17}$ [MH]+: 1380.832; observed: 1380.905. The concentration of peptide for NMR analysis was 6.1 mM.

HPDUGluDap (Ac-Arg Glu Val Thr Val ^{L}Pro Gly Orn Dap Ile Leu Gln-NH_2)

The peptide was synthesized using 205.2 mg (0.051 mmol) of NovaSyn® TGR resin. The synthesis gave 277.8 mg of resin (74.0% yield). The cleavage yielded 55.4 mg of crude peptide (87.1% yield). The peptide was purified by Sep-Pak® Plus Short tC18 cartridges (35% B solvent) and preparative RP-HPLC using a C18 (PLG15_25) column to give 10.7 mg of pure peptide (97.1% purity). Retention time on analytical RP-HPLC was 25.6 minutes. The identity of the peptide was confirmed by MALDI-TOF mass spectrometry. Calculated for $C_{59}H_{105}N_{19}O_{17}$ [MH]+: 1352.801; observed: 1352.945. The concentration of peptide for NMR analysis was 7.9 mM.

HPDUGluDab (Ac-Arg Glu Val Thr Val ^{L}Pro Gly Orn Dab Ile Leu Gln-NH_2)

The peptide was synthesized using 206.2 mg (0.052 mmol) of NovaSyn® TGR resin. The

synthesis gave 282.7 mg of resin (77.0% yield). The cleavage yielded 46.8 mg of crude peptide (69.7% yield). The peptide was purified by Sep-Pak® Plus Short tC18 cartridges (35% B solvent) and preparative RP-HPLC using a C18 (PLG15_25) column to give 6.4 mg of pure peptide (96.9% purity). Retention time on analytical RP-HPLC was 25.5 minutes. The identity of the peptide was confirmed by MALDI-TOF mass spectrometry. Calculated for $C_{60}H_{107}N_{19}O_{17}$ [MH]+: 1366.817; observed: 1366.828. The concentration of peptide for NMR analysis was 9.4 mM.

HPDUGluOrn (Ac-Arg Glu Val Thr Val ^{L}Pro Gly Orn Orn Ile Leu Gln-NH_2)

The peptide was synthesized using 202.6 mg (0.051 mmol) of NovaSyn® TGR resin. The synthesis gave 301.2 mg of resin (>99% yield). The cleavage yielded 68.7 mg of crude peptide (80.0% yield). The peptide was purified by preparative RP-HPLC using C4 (PLG03_13) and C18 (PLG14_24) columns to give 5.3 mg of pure peptide (97.3% purity). Retention time on analytical RP-HPLC was 25.3 minutes. The identity of the peptide was confirmed by MALDI-TOF mass spectrometry. Calculated for $C_{61}H_{109}N_{19}O_{17}$ [MH]+: 1380.832; observed: 1380.804. The concentration of peptide for NMR analysis was 7.7 mM.

HPDUGluLys (Ac-Arg Glu Val Thr Val ^{L}Pro Gly Orn Lys Ile Leu Gln-NH_2)

The peptide was synthesized using 265.0 mg (0.066 mmol) of NovaSyn® TGR resin. The synthesis gave 383.0 mg of resin (>99% yield). The cleavage yielded 69.3 mg of crude peptide (68.0% yield). The peptide was purified by preparative RP-HPLC using a C4 (PLG03_13) column to give 5.7 mg of pure peptide (95.3% purity). Retention time on analytical RP-HPLC was 26.2 minutes. The identity of the peptide was confirmed by MALDI-TOF mass spectrometry. Calculated for $C_{62}H_{111}N_{19}O_{17}$ [MH]+: 1394.848; observed: 1394.900. The concentration of peptide for NMR analysis was 8.2 mM.

HPDUAadDap (Ac-Arg Aad Val Thr Val ^{L}Pro Gly Orn Dap Ile Leu Gln-NH_2)

The peptide was synthesized using 205.7 mg (0.051 mmol) of NovaSyn® TGR resin. The synthesis gave 277.4 mg of resin (73.0% yield). The cleavage yielded 63.1 mg of crude peptide (>99% yield). The peptide was purified by Sep-Pak® Plus Short tC18 cartridges (35% B solvent) and preparative RP-HPLC using a C18 (PLG15_25) column to give 6.1 mg of pure peptide (97.4% purity). Retention time on analytical RP-HPLC was 25.9 minutes. The identity of the peptide was confirmed by MALDI-TOF mass spectrometry. Calculated for $C_{60}H_{107}N_{19}O_{17}$ [MH]+: 1366.817; observed: 1366.944. The concentration of peptide for NMR analysis was 8.9 mM.

HPDUAadDab (Ac-Arg Aad Val Thr Val ^{L}Pro Gly Orn Dab Ile Leu Gln-NH_2)

The peptide was synthesized using 207.0 mg (0.052 mmol) of NovaSyn® TGR resin. The

synthesis gave 279.3 mg of resin (71.7% yield). The cleavage yielded 62.0 mg of crude peptide (>99% yield). The peptide was purified by Sep-Pak® Plus Short tC18 cartridges (35% B solvent) and preparative RP-HPLC using a C18 (PLG15_25) column to give 11.0 mg of pure peptide (96.5% purity). Retention time on analytical RP-HPLC was 25.9 minutes. The identity of the peptide was confirmed by MALDI-TOF mass spectrometry. Calculated for $C_{61}H_{109}N_{19}O_{17}$ [MH]+: 1380.832; observed: 1380.902. The concentration of peptide for NMR analysis was 8.0 mM.

HPDUAadOrn (Ac-Arg Aad Val Thr Val ^{L}Pro Gly Orn Orn Ile Leu Gln-NH_2)

The peptide was synthesized using 208.7 mg (0.052 mmol) of NovaSyn® TGR resin. The synthesis gave 316.9 mg of resin (>99% yield). The cleavage yielded 52.0 mg of crude peptide (57.0% yield). The peptide was purified by Sep-Pak® Plus Short tC18 cartridges (35% B solvent) and preparative RP-HPLC using a C18 (PLG15_25) column to give 7.8 mg of pure peptide (97.2% purity). Retention time on analytical RP-HPLC was 25.7 minutes. The identity of the peptide was confirmed by MALDI-TOF mass spectrometry. Calculated for $C_{62}H_{111}N_{19}O_{17}$ [MH]+: 1394.848; observed: 1394.964. The concentration of peptide for NMR analysis was 5.6 mM.

HPDUAadLys (Ac-Arg Aad Val Thr Val ^{L}Pro Gly Orn Lys Ile Leu Gln-NH_2)

The peptide was synthesized using 208.8 mg (0.052 mmol) of NovaSyn® TGR resin. The synthesis gave 321.4 mg of resin (>99% yield). The cleavage yielded 64.8 mg of crude peptide (67.0% yield). The peptide was purified by Sep-Pak® Plus Short tC18 cartridges (35% B solvent) and preparative RP-HPLC using a C18 (PLG15_25) column to give 9.2 mg of pure peptide (97.2% purity). Retention time on analytical RP-HPLC was 26.0 minutes. The identity of the peptide was confirmed by MALDI-TOF mass spectrometry. Calculated for $C_{63}H_{113}N_{19}O_{17}$ [MH]+: 1408.863; observed: 1408.935. The concentration of peptide for NMR analysis was 6.5 mM.

HPDFAspDap (Ac-Cys Arg Asp Val Thr Val ^{D}Pro Gly Orn Dap Ile Leu Gln Cys-NH_2)

The peptide was synthesized using 207.1 mg (0.050 mmol) of NovaSyn® TGR resin. The synthesis gave 317.4 mg of resin (82.1% yield). The cleavage yielded 65.8 mg of crude peptide (88.8% yield). Retention time on analytical RP-HPLC was 29.8 minutes. The identity of the peptide was confirmed by MALDI-TOF mass spectrometry. Calculated for $C_{64}H_{113}N_{21}O_{19}S_2$ [MH]+: 1544.802; observed: 1544.825. The peptide was dissolved in 1 mM pH 8 phosphate, citrate, and borate buffer at a concentration of 0.1 mg/mL (~0.1 mM). Granulated charcoal was added to the peptide solution, using up to 10:1 (w/w) ratio of charcoal to peptide [3]. After stirring over air for 2 hours, the cyclized peptide was purified by Sep-Pak® Plus Short tC18 cartridges (35% B solvent) and preparative RP-HPLC using a C18

(PLG16_26) column to give 6.0 mg of pure peptide (95.4% purity). Retention time on analytical RP-HPLC was 26.3 minutes. The identity of the peptide was confirmed by MALDI-TOF mass spectrometry. Calculated for $C_{64}H_{111}N_{21}O_{19}S_2$ [MH]+: 1542.788; observed: 1542.786. The concentration of peptide for NMR analysis was 7.8 mM.

HPDFAspDab (Ac-Cys Arg Asp Val Thr Val ^{D}Pro Gly Orn Dab Ile Leu Gln Cys-NH_2)

The peptide was synthesized using 207.1 mg (0.052 mmol) of NovaSyn® TGR resin. The synthesis gave 315.6 mg of resin (80.4% yield). The cleavage yielded 93.2 mg of crude peptide (>99% yield). Retention time on analytical RP-HPLC was 30.4 minutes. The identity of the peptide was confirmed by MALDI-TOF mass spectrometry. Calculated for $C_{65}H_{115}N_{21}O_{19}S_2$ [MH]+: 1558.818; observed: 1558.836. The peptide was dissolved in 1 mM pH 8 phosphate, citrate, and borate buffer at a concentration of 0.1 mg/mL (~0.1 mM). Granulated charcoal was added to the peptide solution, using up to 10:1 (w/w) ratio of charcoal to peptide [3]. After stirring over air for 4 hours, the cyclized peptide was purified by Sep-Pak® Plus Short tC18 cartridges (35% B solvent) and preparative RP-HPLC using a C18 (PLG15_25) column to give 2.0 mg of pure peptide (95.2% purity). Retention time on analytical RP-HPLC was 26.7 minutes. The identity of the peptide was confirmed by MALDI-TOF mass spectrometry. Calculated for $C_{65}H_{113}N_{21}O_{19}S_2$ [MH]+: 1556.804; observed: 1556.803. The concentration of peptide for NMR analysis was 2.6 mM.

HPDFAspOrn (Ac-Cys Arg Asp Val Thr Val ^{D}Pro Gly Orn Orn Ile Leu Gln Cys-NH_2)

The peptide was synthesized using 200.9 mg (0.050 mmol) of NovaSyn® TGR resin. The synthesis gave 320.6 mg of resin (90.8% yield). The cleavage yielded 60.1 mg of crude peptide (70.0% yield). Retention time on analytical RP-HPLC was 30.6 minutes. The identity of the peptide was confirmed by MALDI-TOF mass spectrometry. Calculated for $C_{66}H_{117}N_{21}O_{19}S_2$ [MH]+: 1572.835; observed: 1572.899. The peptide was dissolved in 1 mM pH 8 phosphate, citrate, and borate buffer at a concentration of 0.1 mg/mL (~0.1 mM). Granulated charcoal was added to the peptide solution, using up to 10:1 (w/w) ratio of charcoal to peptide [3]. After stirring over air for 4 hours, the cyclized peptide was purified by preparative RP-HPLC using C4 (PLG09_19) and C18 (PLG15_25) columns to give 0.7 mg of pure peptide (95.9% purity). Retention time on analytical RP-HPLC was 27.0 minutes. The identity of the peptide was confirmed by MALDI-TOF mass spectrometry. Calculated for $C_{66}H_{115}N_{21}O_{19}S_2$ [MH]+: 1570.819; observed: 1570.873. The concentration of peptide for NMR analysis was 0.9 mM.

HPDFAspLys (Ac-Cys Arg Asp Val Thr Val ^{D}Pro Gly Orn Lys Ile Leu Gln Cys-NH_2)

The peptide was synthesized using 201.0 mg (0.050 mmol) of NovaSyn® TGR resin. The synthesis gave 319.0 mg of resin (89.0% yield). The cleavage yielded 62.3 mg of crude

peptide (74.0% yield). Retention time on analytical RP-HPLC was 30.6 minutes. The identity of the peptide was confirmed by MALDI-TOF mass spectrometry. Calculated for $C_{67}H_{119}N_{21}O_{19}S_2$ [MH]+: 1586.851; observed: 1586.682. The peptide was dissolved in 1 mM pH 8 phosphate, citrate, and borate buffer at a concentration of 0.1 mg/mL (~0.1 mM). Granulated charcoal was added to the peptide solution, using up to 10:1 (w/w) ratio of charcoal to peptide [3]. After stirring over air for 4 hours, the cyclized peptide was purified by preparative RP-HPLC using C4 (PLG08_18) and C18 (PLG16_26) columns to give 3.6 mg of pure peptide (96.7% purity). Retention time on analytical RP-HPLC was 26.8 minutes. The identity of the peptide was confirmed by MALDI-TOF mass spectrometry. Calculated for $C_{67}H_{117}N_{21}O_{19}S_2$ [MH]+: 1584.835; observed: 1584.682. The concentration of peptide for NMR analysis was 4.5 mM.

HPDFGluDap (Ac-Cys Arg Glu Val Thr Val ^{D}Pro Gly Orn Dap Ile Leu Gln Cys-NH_2)

The peptide was synthesized using 205.0 mg (0.051 mmol) of NovaSyn® TGR resin. The synthesis gave 309.1 mg of resin (87.3% yield). The cleavage yielded 84.4 mg of crude peptide (>99% yield). Retention time on analytical RP-HPLC was 29.3 minutes. The identity of the peptide was confirmed by MALDI-TOF mass spectrometry. Calculated for $C_{65}H_{115}N_{21}O_{19}S_2$ [MH]+: 1558.818; observed: 1558.929. The peptide was dissolved in 1 mM pH 8 phosphate, citrate, and borate buffer at a concentration of 0.1 mg/mL (~0.1 mM). Granulated charcoal was added to the peptide solution, using up to 10:1 (w/w) ratio of charcoal to peptide [3]. After stirring over air for 2 hours, the cyclized peptide was purified by Sep-Pak® Plus Short tC18 cartridges (35% B solvent) and preparative RP-HPLC using a C18 (PLG16_26) column to give 4.0 mg of pure peptide (95.9% purity). Retention time on analytical RP-HPLC was 26.3 minutes. The identity of the peptide was confirmed by MALDI-TOF mass spectrometry. Calculated for $C_{65}H_{113}N_{21}O_{19}S_2$ [MH]+: 1556.804; observed: 1556.917. The concentration of peptide for NMR analysis was 5.1 mM.

HPDFGluDab (Ac-Cys Arg Glu Val Thr Val ^{D}Pro Gly Orn Dab Ile Leu Gln Cys-NH_2)

The peptide was synthesized using 205.0 mg (0.051 mmol) of NovaSyn® TGR resin. The synthesis gave 273.9 mg of resin (51.2% yield). The cleavage yielded 77.0 mg of crude peptide (>99% yield). Retention time on analytical RP-HPLC was 29.7 minutes. The identity of the peptide was confirmed by MALDI-TOF mass spectrometry. Calculated for $C_{66}H_{117}N_{21}O_{19}S_2$ [MH]+: 1572.834; observed: 1572.993. The peptide was dissolved in 1 mM pH 8 phosphate, citrate, and borate buffer at a concentration of 0.1 mg/mL (~0.1 mM). Granulated charcoal was added to the peptide solution, using up to 10:1 (w/w) ratio of charcoal to peptide [3]. After stirring over air for 4 hours, the cyclized peptide was purified by preparative RP-HPLC using C4 (PLG10_20) and C18 (PLG16_26) columns to give 2.3 mg of pure peptide (96.5% purity). Retention time on analytical RP-HPLC was 26.2 minutes. The

identity of the peptide was confirmed by MALDI-TOF mass spectrometry. Calculated for $C_{66}H_{115}N_{21}O_{19}S_2$ [MH]+: 1570.819; observed: 1570.940. The concentration of peptide for NMR analysis was 3.0 mM.

HPDFGluOrn (Ac-Cys Arg Glu Val Thr Val ^{D}Pro Gly Orn Orn Ile Leu Gln Cys-NH_2)

The peptide was synthesized using 200.2 mg (0.050 mmol) of NovaSyn® TGR resin. The synthesis gave 313.6 mg of resin (85.8% yield). The cleavage yielded 86.9 mg of crude peptide (>99% yield). Retention time on analytical RP-HPLC was 29.6 minutes. The identity of the peptide was confirmed by MALDI-TOF mass spectrometry. Calculated for $C_{67}H_{119}N_{21}O_{19}S_2$ [MH]+: 1586.851; observed: 1586.997. The peptide was dissolved in 1 mM pH 8 phosphate, citrate, and borate buffer at a concentration of 0.1 mg/mL (~0.1 mM). Granulated charcoal was added to the peptide solution, using up to 10:1 (w/w) ratio of charcoal to peptide [3]. After stirring over air for 4 hours, the cyclized peptide was purified by preparative RP-HPLC using C4 (PLG08_18) and C18 (PLG16_26) columns to give 1.5 mg of pure peptide (96.2% purity). Retention time on analytical RP-HPLC was 26.7 minutes. The identity of the peptide was confirmed by MALDI-TOF mass spectrometry. Calculated for $C_{67}H_{117}N_{21}O_{19}S_2$ [MH]+: 1584.851; observed: 1584.803. The concentration of peptide for NMR analysis was 1.9 mM.

HPDFGluLys (Ac-Cys Arg Glu Val Thr Val ^{D}Pro Gly Orn Lys Ile Leu Gln Cys-NH_2)

The peptide was synthesized using 204.2 mg (0.051 mmol) of NovaSyn® TGR resin. The synthesis gave 33.1 mg of resin (99.8% yield). The cleavage yielded 84.6 mg of crude peptide (88.3% yield). Retention time on analytical RP-HPLC was 30.0 minutes. The identity of the peptide was confirmed by MALDI-TOF mass spectrometry. Calculated for $C_{68}H_{121}N_{21}O_{19}S_2$ [MH]+: 1600.866; observed: 1600.043. The peptide was dissolved in 1 mM pH 8 phosphate, citrate, and borate buffer at a concentration of 0.1 mg/mL (~0.1 mM). Granulated charcoal was added to the peptide solution, using up to 10:1 (w/w) ratio of charcoal to peptide [3]. After stirring over air for 4 hours, the cyclized peptide was purified by Sep-Pak® Plus Short tC18 cartridges (25% B solvent) and preparative RP-HPLC using a C18 (PLG15_25) column to give 3.2 mg of pure peptide (95.5% purity). Retention time on analytical RP-HPLC was 27.5 minutes. The identity of the peptide was confirmed by MALDI-TOF mass spectrometry. Calculated for $C_{68}H_{119}N_{21}O_{19}S_2$ [MH]+: 1598.851; observed: 1599.043. The concentration of peptide for NMR analysis was 3.2 mM.

HPDFAadDap (Ac-Cys Arg Aad Val Thr Val ^{D}Pro Gly Orn Dap Ile Leu Gln Cys-NH_2)

The peptide was synthesized using 205.2 mg (0.051 mmol) of NovaSyn® TGR resin. The synthesis gave 324.3 mg of resin (95.9% yield). The cleavage yielded 83.8 mg of crude peptide (>99% yield). Retention time on analytical RP-HPLC was 29.5 minutes. The identity

of the peptide was confirmed by MALDI-TOF mass spectrometry. Calculated for $C_{66}H_{117}N_{21}O_{19}S_2$ [MH]+: 1572.834; observed: 1572.906. The peptide was dissolved in 1 mM pH 8 phosphate, citrate, and borate buffer at a concentration of 0.1 mg/mL (~0.1 mM). Granulated charcoal was added to the peptide solution, using up to 10:1 (w/w) ratio of charcoal to peptide [3]. After stirring over air for 2 hours, the cyclized peptide was purified by Sep-Pak® Plus Short tC18 cartridges (35% B solvent) and preparative RP-HPLC using a C18 (PLG16_26) column to give 5.3 mg of pure peptide (95.0% purity). Retention time on analytical RP-HPLC was 27.0 minutes. The identity of the peptide was confirmed by MALDI-TOF mass spectrometry. Calculated for $C_{66}H_{115}N_{21}O_{19}S_2$ [MH]+: 1570.819; observed: 1570.902. The concentration of peptide for NMR analysis was 6.8 mM.

HPDFAadDab (Ac-Cys Arg Aad Val Thr Val ^{D}Pro Gly Orn Dab Ile Leu Gln Cys-NH_2)

The peptide was synthesized using 207.1 mg (0.052 mmol) of NovaSyn® TGR resin. The synthesis gave 360.0 mg of resin (>99% yield). The cleavage yielded 93.2 mg of crude peptide (84.7% yield). Retention time on analytical RP-HPLC was 29.8 minutes. The identity of the peptide was confirmed by MALDI-TOF mass spectrometry. Calculated for $C_{67}H_{119}N_{21}O_{19}S_2$ [MH]+: 1586.89; observed: 1586.962. The peptide was dissolved in 1 mM pH 8 phosphate, citrate, and borate buffer at a concentration of 0.1 mg/mL (~0.1 mM). Granulated charcoal was added to the peptide solution, using up to 10:1 (w/w) ratio of charcoal to peptide [3]. After stirring over air for 4 hours, the cyclized peptide was purified by Sep-Pak® Plus Short tC18 cartridges (35% B solvent) and preparative RP-HPLC using a C18 (PLG17_27) column to give 3.2 mg of pure peptide (95.4% purity). Retention time on analytical RP-HPLC was 26.9 minutes. The identity of the peptide was confirmed by MALDI-TOF mass spectrometry. Calculated for $C_{67}H_{117}N_{21}O_{19}S_2$ [MH]+: 1584.835; observed: 1584.945. The concentration of peptide for NMR analysis was 4.0 mM.

HPDFAadOrn (Ac-Cys Arg Aad Val Thr Val ^{D}Pro Gly Orn Orn Ile Leu Gln Cys-NH_2)

The peptide was synthesized using 200.8 mg (0.050 mmol) of NovaSyn® TGR resin. The synthesis gave 320.7 mg of resin (90.0% yield). The cleavage yielded 73.6 mg of crude peptide (85.0% yield). Retention time on analytical RP-HPLC was 29.8 minutes. The identity of the peptide was confirmed by MALDI-TOF mass spectrometry. Calculated for $C_{68}H_{121}N_{21}O_{19}S_2$ [MH]+: 1600.866; observed: 1600.890. The peptide was dissolved in 1 mM pH 8 phosphate, citrate, and borate buffer at a concentration of 0.1 mg/mL (~0.1 mM). Granulated charcoal was added to the peptide solution, using up to 10:1 (w/w) ratio of charcoal to peptide [3]. After stirring over air for 4 hours, the cyclized peptide was purified by preparative RP-HPLC using C4 (PLG06_16) and C18 (PLG16_26) columns to give 4.2 mg of pure peptide (95.8% purity). Retention time on analytical RP-HPLC was 27.0 minutes. The identity of the peptide was confirmed by MALDI-TOF mass spectrometry. Calculated for

$C_{68}H_{119}N_{21}O_{19}S_2$ [MH]+: 1598.852; observed: 1598.894. The concentration of peptide for NMR analysis was 2.6 mM.

HPDFAadLys (Ac-Cys Arg Aad Val Thr Val ^{D}Pro Gly Orn Lys Ile Leu Gln Cys-NH_2)

The peptide was synthesized using 200.0 mg (0.050 mmol) of NovaSyn® TGR resin. The synthesis gave 321.5 mg of resin (91.1% yield). The cleavage yielded 75.7 mg of crude peptide (87.0% yield). Retention time on analytical RP-HPLC was 29.9 minutes. The identity of the peptide was confirmed by MALDI-TOF mass spectrometry. Calculated for $C_{69}H_{123}N_{21}O_{19}S_2$ [MH]+: 1614.882; observed: 1614.882. The peptide was dissolved in 1 mM pH 8 phosphate, citrate, and borate buffer at a concentration of 0.1 mg/mL (~0.1 mM). Granulated charcoal was added to the peptide solution, using up to 10:1 (w/w) ratio of charcoal to peptide [3]. After stirring over air for 4 hours, the cyclized peptide was purified by preparative RP-HPLC using C4 (PLG07_17) and C18 (PLG16_26) columns to give 2.9 mg of pure peptide (96.6% purity). Retention time on analytical RP-HPLC was 26.8 minutes. The identity of the peptide was confirmed by MALDI-TOF mass spectrometry. Calculated for $C_{69}H_{121}N_{21}O_{19}S_2$ [MH]+: 1612.868; observed: 1612.910. The concentration of peptide for NMR analysis was 3.6 mM.

Reference

1. Atherton, E.; Fox, H.; Harkiss, D.; Logan, C. J.; Sheppard, R. C.; Williams, B. J. A mild procedure for solid phase peptide synthesis: use of fluorenylmethoxycarbonylamino-acids. *J. Chem. Soc., Chem. Commun.* **1978**, 537-539.
2. Fields, G. B.; Noble, R. L. Solid phase peptide synthesis utilizing 9-fluorenylmethoxycarbonyl amino acids. *Int. J. Pept. Protein Res.* **1990,** *35*, 161-214.
3. Volkmer-Engert, R.; Landgraf, C.; Schneider-Mergener, J. Charcoal surface-assisted catalysis of intramolecular disulfide bond formation in peptides. *J. Pept. Res.* **1998,** *51*, 365-369.
